# The mPFC-reuniens-hippocampus pathway links brain circuitry and neural plasticity in antidepressant response

**DOI:** 10.1038/s41467-026-77248-y

**Published:** 2026-09-01

**Authors:** Maxime Veleanu, Louise Schuberth, Antje Kilias, Jakob Weber, Jan Warneke, Lovis Würz, Tim Schwär, Rebecca Heck, Joelle Müller, David H. Sarrazin, Marguerite Anselin, Lukas Rutke, Guillermo Suarez Marchi, Stella Zimmermann, Zoe Borgeest, Martin Balzinger, Alina Blendinger, Anna Catarata, Samira Assaad Dib, Yaroslav Sych, Thibault Cholvin, Marlene Bartos, Katharina Domschke, Claus Normann, Stefan Vestring, Tsvetan Serchov

**Affiliations:** 1 Department of Psychiatry and Psychotherapy, Medical Center—University of Freiburg, Faculty of Medicine, University of Freiburg, Freiburg, Germany; 2 Spemann Graduate School of Biology and Medicine (SGBM), Freiburg, Germany; 3 Faculty of Biology, University of Freiburg, Freiburg, Germany; 4 Institute for Physiology I, Medical Faculty, University of Freiburg, Freiburg, Germany; 5 Centre National de la Recherche Scientifique (CNRS), Institute of Cellular and Integrative Neurosciences (INCI) UPR, University of Strasbourg, Strasbourg, 3212 France; 6 University of Strasbourg Institute for Advanced Study (USIAS), Strasbourg, France; 7 German Center for Mental Health (DZPG), Partner Site Berlin/Potsdam, Berlin, Germany

**Keywords:** Neural circuits, Depression, Synaptic plasticity, Stress and resilience

## Abstract

The pathophysiology of depression involves multiple biological processes, including circuit dysfunction and impaired neuroplasticity, yet an integrative view linking these processes remains elusive. Here, we identify a convergent circuit for antidepressant response and plasticity modulation. We demonstrate that chemogenetic activation of the infralimbic cortex (IL) exerts rapid antidepressant-like effects across multiple behavioral domains in a mouse model of stress-induced depression. IL stimulation exerts top-down control over the hippocampus, enhancing structural plasticity, restoring long-term potentiation deficits and improving state-dependent network dynamics in the ventral hippocampus (vHIPP). We identify the thalamic nucleus reuniens (RE) as a necessary mediator of these effects. Notably, direct inhibition of RE, its inputs from IL or projections to vHIPP, blocks both IL stimulation-induced antidepressant response and the therapeutic and neuroplastic effects of ketamine. Our findings demonstrate that the functional IL → RE→vHIPP circuit plays a central role in the antidepressant response, linking circuit activity, hippocampal plasticity, and depressive-like behaviors.

## Abstract (web summary)

Neural mechanisms underlying depression are not fully understood. This study identifies a prefrontal–thalamic–hippocampal circuit that links antidepressant-like behavior with restored neural plasticity and is required for ketamine’s behavioral and neuroplastic effects in mice.

## Introduction

Major depressive disorder (MDD) is a widespread and devastating condition that causes immense suffering and heavy socioeconomic burdens^1^. The treatments that are currently available have slow mechanisms of action and can increase suicidal ideation. Additionally, the high proportion of nonresponders among patients who receive these treatments highlights the urgent need for new, fast-acting antidepressants^2^.

The discovery of the rapid antidepressant effects of ketamine has challenged the monoaminergic deficit hypothesis of MDD, providing new perspectives that have led to the proposition of alternative frameworks for understanding the pathophysiology of depression and treating this condition^3,4^. For example, the neurocircuit hypothesis postulates that depression might occur due to dysfunctional information processing and connectivity patterns in specific key networks^5^, whereas the neuroplasticity hypothesis proposes that an impaired ability to modify synaptic strength and neural connections underlies depressive pathology^6,7^. Correspondingly, stimulation of selected brain circuits and regions or enhancement of synaptic plasticity promotes antidepressant response^8,9^. Despite being extensively studied in humans and animal models, the link or potential interdependence between these two frameworks remains poorly understood.

Neuroimaging studies, postmortem analyses, and preclinical research have indicated that depression is associated with structural and functional deregulations in a subset of brain regions^10–12^. The prefrontal cortex has emerged as one of the regions that is most consistently impaired in patients with depression^13^, with the most consistent volumetric and structural deficits, such as cortical atrophy or a decrease in synaptic density^14^. More recently, the largest study involving precision functional mapping to date identified a strong role of the frontostriatal salience network, comprising the anterior insula and dorsal anterior cingulate cortex in depression, even allowing for the prediction of the emergence of depressive symptoms^15^. At the preclinical level, multiple studies have demonstrated structural and functional deficits in the medial prefrontal cortex (mPFC) of rodent models of depression, and successful treatments with antidepressant therapies often involve prefrontal mechanisms^16,17^. Therapeutic interventions that impact prefrontal activity, including transcranial magnetic stimulation (TMS)^18^, deep brain stimulation (DBS)^9^, or treatment with the fast-acting antidepressant ketamine, ameliorate depressive-like phenotypes. Finally, the mPFC, especially the infralimbic (IL) region, is extensively connected to limbic brain structures, such as the nucleus accumbens (NAc), the ventral tegmental area (VTA) and the basolateral amygdala (BLA)^19^, which are implicated in the control of reward, motivation, emotion regulation, and mood^12,20,21^; these findings highlight the role of the mPFC as a central hub in depression^22^.

Neuroplasticity is the ability of the brain to undergo biological changes in response to a changing environment. Neuroplasticity is thought to play a major role in depression by modulating the brain’s response to aversive experiences or chronic stress^23^. Several paradigms that provide indirect readouts of plasticity-related processes in humans, such as paired associative stimulation and recording motor^24^ or visual^25^ evoked potentials, revealed a decrease in the correlates of stimulus-response plasticity in cortex areas of depressed patients, which was reversed by antidepressant therapy. Both structural (synaptic density) and associative plasticity, which are two mechanisms that are crucial for learning, have been extensively studied in the hippocampus (HIPP). Rodent models of depression have systematically shown impaired long-term potentiation (LTP)^26,27^, and the antidepressant effect of drugs such as ketamine has been associated with the amelioration of LTP deficits in the HIPP, suggesting a major role for associative plasticity in MDD and antidepressant response^28^. Despite the abundant evidence supporting major roles of the PFC and the HIPP in depression, an integrative investigation linking prefrontal circuits with hippocampal neuroplasticity in the context of depression treatment is lacking.

In this study, we initially confirm the potential of mPFC stimulation to elicit antidepressant effects using a chemogenetic approach (designer receptors exclusively activated by designer drugs; DREADDs). Next, we unravel the downstream circuit mechanisms and show that the mPFC exerts a top-down control on hippocampal structural plasticity, LTP inducibility and information processing. Notably, we identify the nucleus reuniens (RE) as a necessary link between the mPFC and HIPP for mediating antidepressant and neuroplastic effects of mPFC stimulation and ketamine. Overall, our findings support a central role for the mPFC–RE–HIPP circuit in the response to antidepressants, acting as a loop controlling HIPP plasticity and depressive-like behavior.

## Results

### Prolonged chemogenetic activation of IL excitatory neurons exerts antidepressant-like effects in various behavioral paradigms

**Fig. 1:**
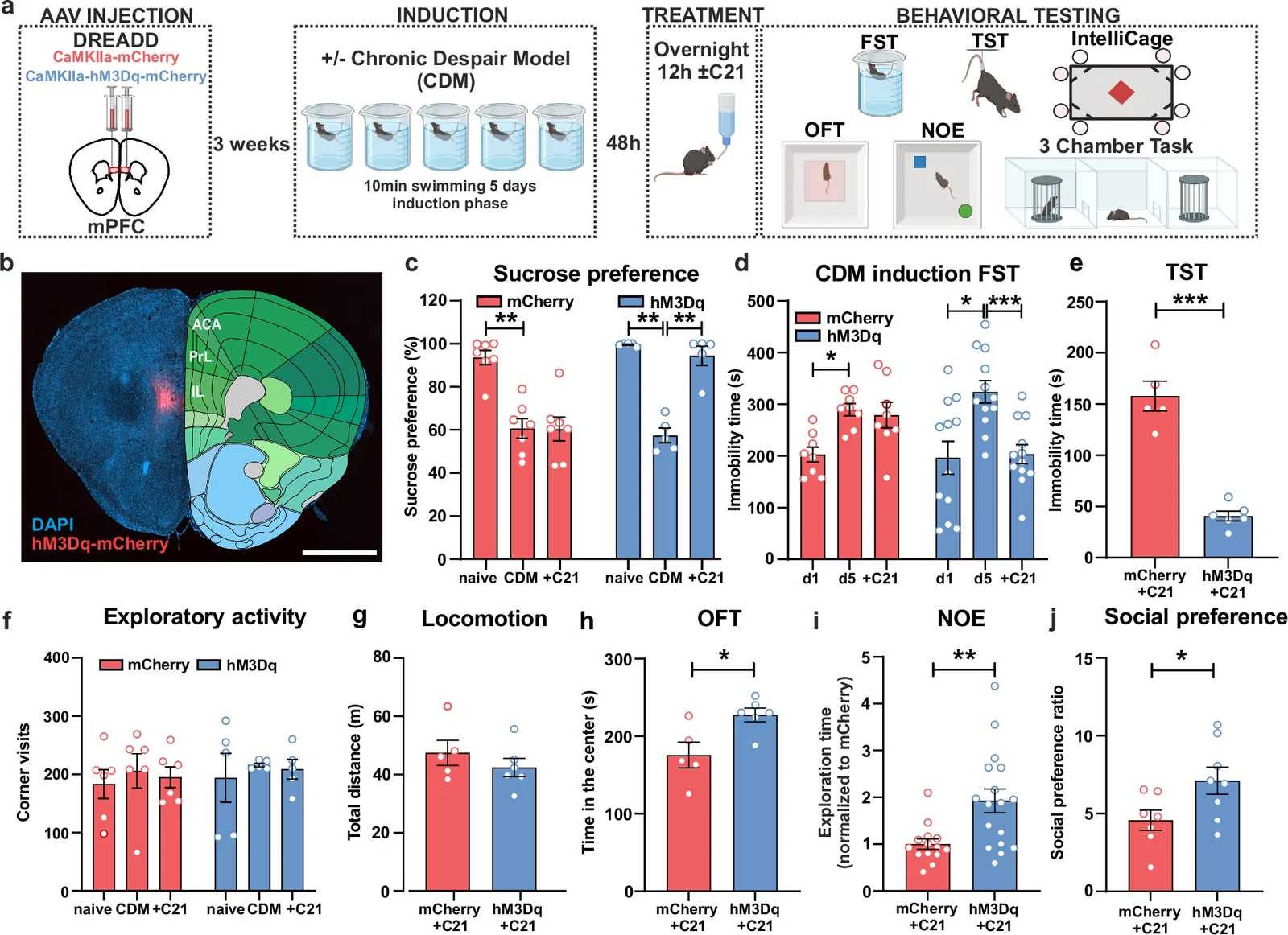
Prefrontal IL DREADD stimulation produces antidepressant-like effects in various behavioral paradigms. **a** Experimental design for viral targeting of the infralimbic cortex (IL), CDM induction, overnight C21 treatment and behavioral testing. **b** Image of AAV-CaMKIIα-hM3D(Gq)-mCherry injection in the IL, co-registered with the Allen Brain Atlas. Scale bar: 1000 μm. Representative of *n* = 9 animals, see Supplementary Fig. 1c. PrL: prelimbic; ACC: anterior cingulate; DP: dorsal peduncular; TTd: dorsal taenia tecta. **c** Sucrose preference measured in IntelliCage of naïve, CDM and overnight C21 treated mCherry (*n *= 7; CDM vs naive *P* = 0.0088; CDM vs +24 h P > 0.9999) and hM3Dq-injected (*n* = 5; CDM vs naive *P* = 0.0010; CDM vs +24 h P = 0.0068) mice. Repeated measures two-way ANOVA with Bonferroni post-hoc test. **d** Immobility time in CDM paradigm from day 1, day 5 and after overnight C21 treatment of mCherry (*n* = 8; day 1 vs day 5 *P* = 0.0118; day 5 vs +C21 *P* > 0.9999) and hM3Dq (*n* = 12; day 1 vs day 5 *P* = 0.0366; day 5 vs +C21 *P* = 0.0003) mice. Repeated measures two-way ANOVA with Bonferroni post-hoc test. **e** Immobility time in tail suspension test (TST) (hM3Dq *n* = 6; mCherry *n* = 5, *P* = 0.0007, two-sided unpaired Welch’s t-test). **f** Global exploratory activity measured by corner visits in IntelliCage (mCherry: *n* = 6, hM3Dq: *n* = 5, repeated measures ANOVA with Bonferroni post-hoc test). **g** Locomotion measured by distance traveled in open field test (OFT) of mCherry (*n* = 5) and hM3Dq mice (*n* = 6) (*P* = 0.3634, two-sided unpaired Student’s *t*-test). **h** Time spent in the central zone of OFT of hM3Dq-injected mice (*n* = 6) compared to mCherry controls (*n *= 5) (*P* = 0.0183, two-sided unpaired Student’s *t*-test). **i** Exploratory behavior measured by total time spent exploring two novel objects in novel object exploration (NOE) (mCherry *n *= 14, hM3Dq n = 17, *P* = 0.0027, two-sided Welch’s t-test). Data normalized to control group. **j** Social preference measured as time spent exploring a caged visitor mouse divided by time spent exploring a novel object in three-chamber task (mCherry *n* = 7, hM3Dq *n* = 8, *P* = 0.0415, two-sided unpaired Student’s *t*-test). Data are presented as mean ± SEM and the individual data points are depicted. *P < 0.05, **P < 0.01, ***P < 0.001. See also Supplementary Fig. 3. Source data are provided as a Source Data file. Created in BioRender. Vestring, S. (2026) https://BioRender.com/rpcq0ko.

To investigate the role of excitatory neurons in the mPFC, we stereotactically injected an adeno-associated virus expressing the chemogenetic activator hM3Dq or an inactive mCherry control under the control of the CaMKIIα promoter into the IL (Fig. 1a–b; Supplementary Fig. 1a–c). To confirm the efficacy of the chemogenetic stimulation, we conducted whole-cell recordings from IL pyramidal neurons expressing hM3Dq-mCherry; recorded cells were filled with biocytin and post hoc verified within the virally targeted IL area (Supplementary Fig. 2a–c). Bath application of the DREADD agonist Compound C21^29^ increased excitatory postsynaptic potentials (EPSP) amplitude (Supplementary Fig. 2f–h), enhanced EPSP–spike coupling (Supplementary Fig. 2i, j), and reduced spike latency during depolarizing current injections (Supplementary Fig. 2d, e), indicating increased intrinsic and synaptically driven excitability and a net excitatory effect on IL pyramidal neurons embedded in the local network.

To induce a depressive-like state, we utilized the chronic despair model (CDM), which involves subjecting animals to 10-min forced swim sessions daily for five consecutive days (Fig. 1a)^30^. This model yields a long-lasting depression-like phenotype, including increased immobility time in the classical forced swim test (FST) and tail suspension test (TST) and decreased sucrose preference (Fig. 1c-e; Supplementary Fig. 3m); moreover, this model is sensitive to both chronic monoaminergic antidepressants and acute ketamine treatment^16,28,31,32^.

We used a multimodal approach to assess various aspects of depressive-like behavior (Fig. 1a). Two days after CDM induction, we administered C21 (0.75 mg/kg) via the drinking water overnight. To evaluate motivation and reward-oriented behavior, we conducted the nose-poke sucrose preference test (NP-SPT) in an IntelliCage^16,32,33^ following a progressive ratio reinforcement schedule. Consistent with previous findings^16,32^, the CDM resulted in significant anhedonia-like behavior, and this phenotype was reversed by overnight C21 treatment in mice expressing hM3Dq receptors in principal cells but not in mice expressing the mCherry control (Fig. 1c). Similarly, the CDM increased immobility time in both the FST (Fig. 1d) and TST (Supplementary Fig. 3m), and IL-specific DREADD activation restored the normal phenotype (Fig. 1d, e). Notably, acute C21 administration (0.75 mg/kg, i.p.) did not produce significant antidepressant-like effects in the FST or TST (Supplementary Fig. 3k, l), indicating that overnight, but not acute, IL activation is required for robust behavioral effects. Neither exploratory activity in the IntelliCage (measured by corner visits) nor global locomotion in the open field test (OFT) was affected by CDM establishment (Fig. 1f and Supplementary Fig. 3o) or IL stimulation (Fig. 1f, g); these results excluded the possibility that DREADD stimulation induced hyperactivity. While not a direct feature of depression per se, anxious behavior is a common consequence of chronic stress. In the OFT, C21 treatment increased the time the hM3Dq-expressing mice spent in the center compared to control mice (Fig. 1h), indicating reduced anxiety-like behavior.

To assess the drive to investigate novelty, we conducted a novel object exploration (NOE) test in which two novel objects were presented simultaneously without prior familiarization. IL activation significantly increased the cumulative time spent exploring both objects compared to control mice (Fig. 1i, Supplementary Fig. 3e), indicating increased interest in novel stimuli, although NOE effect on CDM versus naive mice did not reach significance (Supplementary Fig. 3n). Social deficits represent a major component of depressive symptomatology and are both a consequence of and a risk factor for MDD^34,35^. Using the three-chamber task, we measured sociability as the ratio of time spent exploring a novel social stimulus versus a novel object. IL stimulation significantly increased social preference in hM3Dq-expressing mice compared to mCherry control-expressing mice (Fig. 1j, Supplementary Fig. 3f), suggesting enhanced sociability.

Additionally, to verify DREADD specificity, we compared hM3Dq-expressing mice that received either C21-supplemented water or regular drinking water as a control. Here, hM3Dq activation exerted antidepressant-like effects in the FST, TST, and NOE, although the effects in the social preference test did not reach significance (Supplementary Fig. 3a–jf); these results confirmed that the antidepressant-like effects are specific to IL stimulation rather than unspecific effects of DREADD expression.

To establish the specificity of the role of IL in the antidepressant effect of mPFC stimulation, we conducted parallel DREADD experiments targeting the prelimbic cortex (PrL), which is another key mPFC subdivision that is located dorsal to the IL (Supplementary Figs. 3g and 1e). PrL activation did not affect immobility time or anhedonia-like behavior (Supplementary Fig. 3h–j), demonstrating that antidepressant-like effects specifically require IL, but not PrL activation.

### IL stimulation rescues CDM-induced abolition of hippocampal synaptic plasticity

**Fig. 2:**
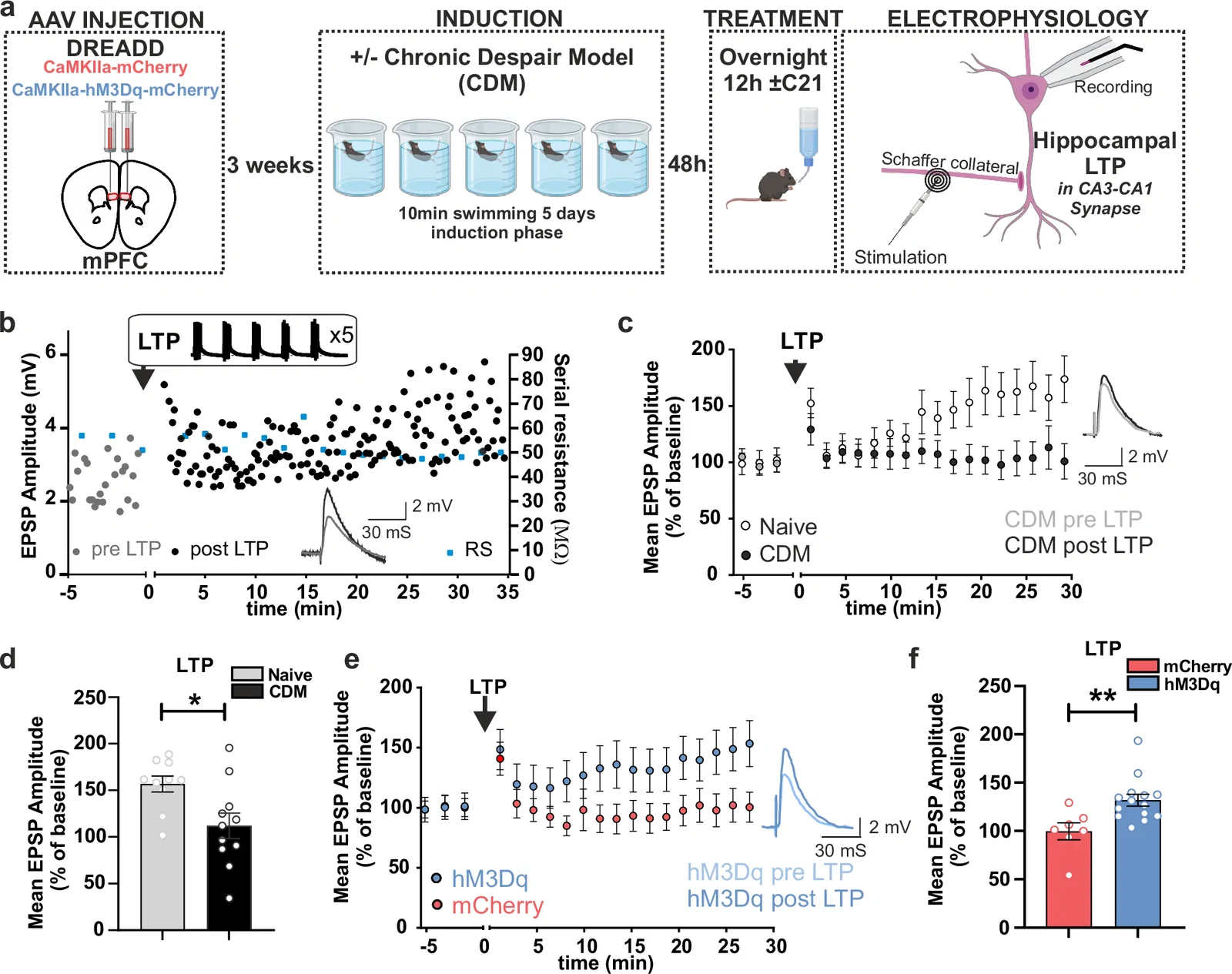
Prefrontal IL DREADDs rescues CDM-induced deficits in associative plasticity. **a** Experimental design for viral targeting of the infralimbic cortex (IL), chronic despair model (CDM) induction, overnight C21 treatment and ex vivo hippocampal CA3–CA1 LTP recordings. **b** Representative aLTP experiment. Each gray dot represents the EPSP amplitudes before an LTP induction protocol (125 EPSP → AP pairings in TBS frequency (upper inset)) and each black dot after the induction protocol. Blue squares represent the serial resistance throughout the experiment. Please note the increase of the exemplary EPSP traces from before (gray trace) to 30 min after the LTP induction protocol (black trace). **c** Time course of aLTP inducibility comparing CDM (black) and naïve (white) conditions with exemplary EPSP traces (naïve: *n* = 10, CDM: *n* = 11 cells). **d** Group analysis (naïve: *n* = 10, CDM: *n* = 11 cells, *P* = 0.0135, two-sided Student’s *t*-test). **e** Time course of aLTP inducibility after overnight DREADD activation comparing hM3Dq (blue) and mCherry (red) groups with exemplary EPSP traces from an hM3Dq injected mouse (mCherry *n* = 7, hM3Dq *n* = 14 cells). **f** Group analysis (mCherry *n* = 7, hM3Dq *n* = 14 cells, *P* = 0.0023, two-sided Mann-Whitney test). Data are presented as mean ± SEM and the individual data points are depicted. *P < 0.05, **P < 0.01, ***P < 0.001. See also Supplementary Fig. 4. Source data are provided as a Source Data file. Created in BioRender. Vestring, S. (2026) https://BioRender.com/rpcq0ko.

Chronic stress has been shown to disrupt hippocampal synaptic plasticity and this effect can be rescued by antidepressants such as ketamine or long-term fluoxetine treatment^28,36,37^. To investigate the effects of IL stimulation on hippocampal plasticity, we performed ex vivo recordings of CA1 pyramidal neurons in CDM mice. We induced associative LTP (aLTP) by pairing subthreshold EPSPs evoked by Schaffer collateral stimulation with postsynaptic action potentials (125 EPSP → AP pairings at the theta-burst frequency; Fig. 2a, b). This resulted in a stable increase in EPSPs after 20–30 min in naive mice, whereas no such increase was observed in CDM mice, demonstrating a complete abolition of LTP (Fig. 2c, d). CDM-induced hippocampal aLTP blockade could be completely reversed by in vivo overnight IL activation (Fig. 2e, f), while paired-pulse ratio and membrane properties remained unchanged (Supplementary Fig. 4a-h, n), consistent with a postsynaptic associative plasticity mechanism. Importantly, acute intraperitoneal (i.p.) C21 administration (2 h) did not restore aLTP (Supplementary Fig. 4i-m), whereas overnight administration fully rescued plasticity, suggesting that prolonged IL activation is required for hippocampal plasticity restoration. Overall, these results show that IL stimulation restored chronic-stress-induced hippocampal aLTP deficits, demonstrating that PFC activity modulates hippocampal synaptic plasticity.

### IL activation modulates hippocampal microcircuit dynamics

**Fig. 3:**
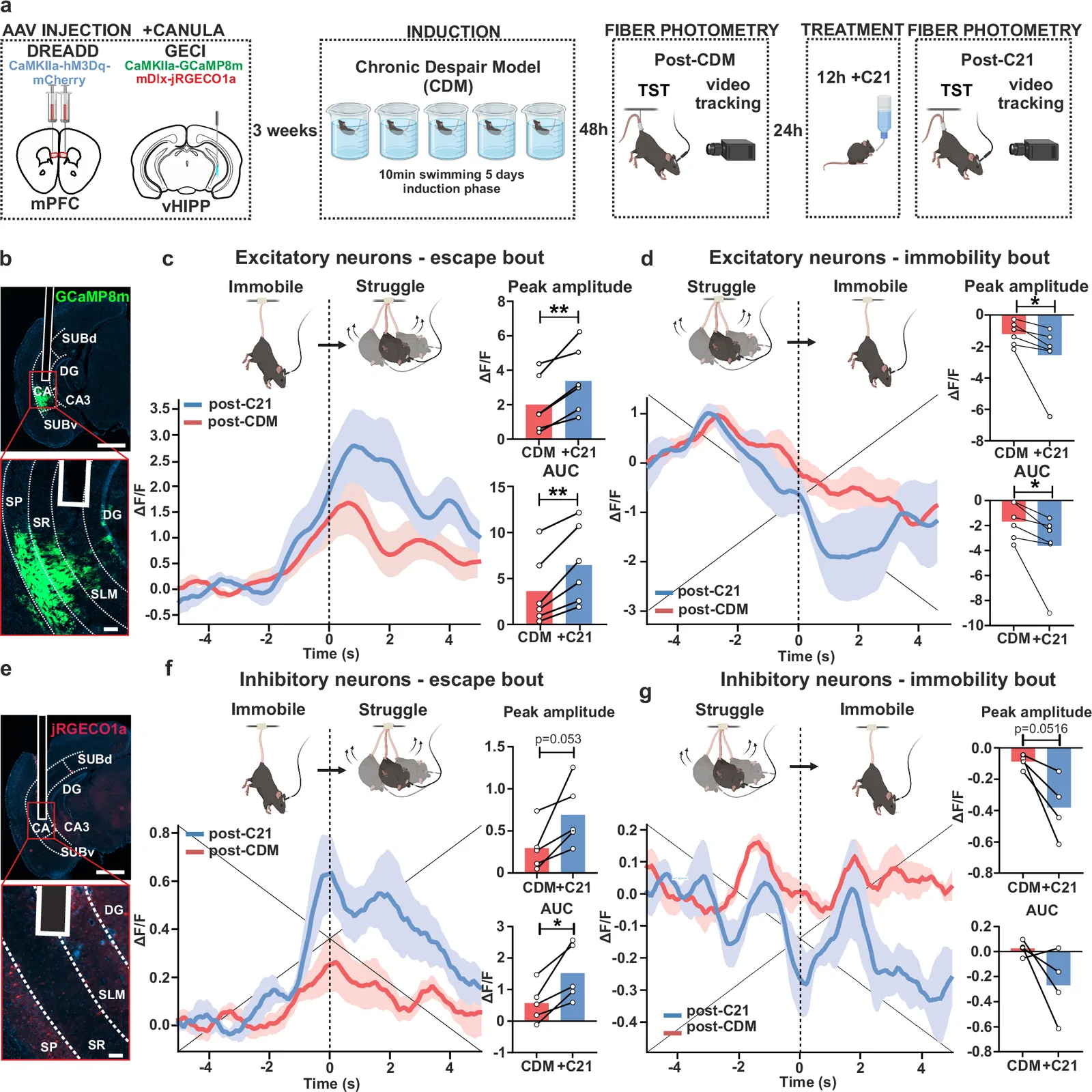
State-dependent modulation of hippocampal circuit dynamics following IL activation. **a** Experimental design for fiber photometry of vHIPP excitatory (CaMKIIα-GCaMP8m) and inhibitory (mDlx-jRGECO1a) neurons in IL-hM3Dq mice, recorded during the tail suspension test (TST) after CDM (post-CDM) and after overnight C21 (post-C21). **b** Microscopy images of GCaMP8m expression and fiber placement in the ventral hippocampus, representative of n = 6 mice. Top: hippocampal subregions (SUBd/SUBv: dorsal/ventral subiculum; CA1; CA3; DG: dentate gyrus), scale bar 1000 µm. Bottom: magnified image showing fiber tip and layers (SP: stratum pyramidale; SR: stratum radiatum; SLM: stratum lacunosum-moleculare), scale bar 100 µm. **c** Excitatory neurons during immobile-to-struggle transitions. Left: average ΔF/F aligned to the behavioral state transitions (post-CDM: red; post-C21: blue). Right: peak amplitude and area under the curve (AUC) over the transition period ( ± 5 s) (*n *= 6 mice per group; peak amplitude: *P* = 0.0044; AUC: *P* = 0.0036; two-sided paired Student’s t-test). **d** Excitatory neurons during struggle-to-immobile transitions. Left: average ΔF/F aligned to the behavioral state transitions (post-CDM: red; post-C21: blue). Right: peak amplitude and AUC over the transition period ( ± 5 s) (*n* = 6 mice per group; peak amplitude: *P* = 0.0313; AUC: *P *= 0.0313; two-sided Wilcoxon matched-pairs signed rank test). **e** Microscopy images of jRGECO1a expression and fiber placement in the ventral hippocampus, representative of *n* = 5 mice (top: hippocampal subregions, scale bar 1000 µm; bottom: magnified image showing fiber tip and hippocampal layers, scale bar 100 µm). **f** Inhibitory neurons during immobile-to-struggle transitions. Left: average ΔF/F aligned to the behavioral state transitions (post-CDM: red; post-C21: blue). Right: peak amplitude and AUC over the transition period ( ± 5 s) (*n* = 5 mice per group; peak amplitude: P = 0.0534; AUC: *P* = 0.0178; two-sided paired Student’s *t*-test). **g** Inhibitory neurons during struggle-to-immobile transitions. Left: average ΔF/F aligned to the behavioral state transitions (post-CDM: red; post-C21: blue). Right: peak amplitude and AUC over the transition period ( ± 5 s) (*n* = 4 mice per group; peak amplitude: *P* = 0.0516; AUC: *P* = 0.1569; two-sided paired Student’s *t*-test). Data are presented as mean ± SEM with individual data points shown; shaded areas indicate SEM. *P < 0.05, **P < 0.01, ***P < 0.001. See also Supplementary Fig. 5. Source data are provided as a Source Data file. Created in BioRender. Vestring, S. (2026) https://BioRender.com/rpcq0ko.

To investigate how IL stimulation affects HIPP circuit dynamics, we used in vivo fiber photometry recordings in the ventral hippocampus (vHIPP) of freely moving mice, expressing hM3Dq receptors in IL principal cells and cell type-specific genetically encoded calcium indicators in the vHIPP. We recorded both excitatory (CaMKIIα-GCaMP8m) and inhibitory (mDlx-jRGECO1a) vHIPP neurons (Fig. 3a, b, e; Supplementary Fig. 5a). We monitored Ca^2+^ signals in mice performing the TST 48 h after CDM induction and following overnight C21 treatment. Using a custom-trained DeepLabCut pose estimation model^38^, we identified transitions between active (struggle) and passive (immobility) states.

Our analyses revealed increased peak amplitudes and areas under the curve (AUCs) of excitatory neurons during bouts of struggle after C21 treatment (Fig. 3c). Conversely, transitions to immobility were associated with decreased excitatory neuron Ca^2+^ activity, and IL stimulation resulted in increased negative deflection in both the peak amplitudes and AUCs (Fig. 3d). While inhibitory neuron responses during bouts of struggle increased after C21 treatment, only the difference in the AUC was significant (Fig. 3f, g). The transient mean peak amplitudes during full periods of immobility or struggle remained unchanged after IL activation (Supplementary Fig. 5j, k). Control analyses confirmed that Ca^2+^ dynamics were not driven by motor confounds: no significant correlation was observed between movement intensity and signal amplitude for either indicator, and motor activity profiles during struggle onset were comparable between conditions despite divergent Ca^2+^ responses (Supplementary Fig. 5d-i). Furthermore, analysis of the transition from spontaneous quiet wakefulness to active exploration in the homecage revealed no significant difference in Ca^2+^ activity (Supplementary Fig. 5b, c). These findings demonstrate that IL activation bidirectionally modulates HIPP activity during behavioral transition in the TST, but not homecage exploration, increasing excitatory neuron responses during struggle and further suppressing these responses during immobility, with concurrent changes in inhibitory neuron dynamics. This increase in state-dependent dynamics suggests that IL activation improves hippocampal gain in stress-related behavior, potentially contributing to its antidepressant effects.

### Prefrontal IL stimulation induces hippocampal plasticity-related Immediate Early Genes (IEGs) in a region-specific manner

**Fig. 4:**
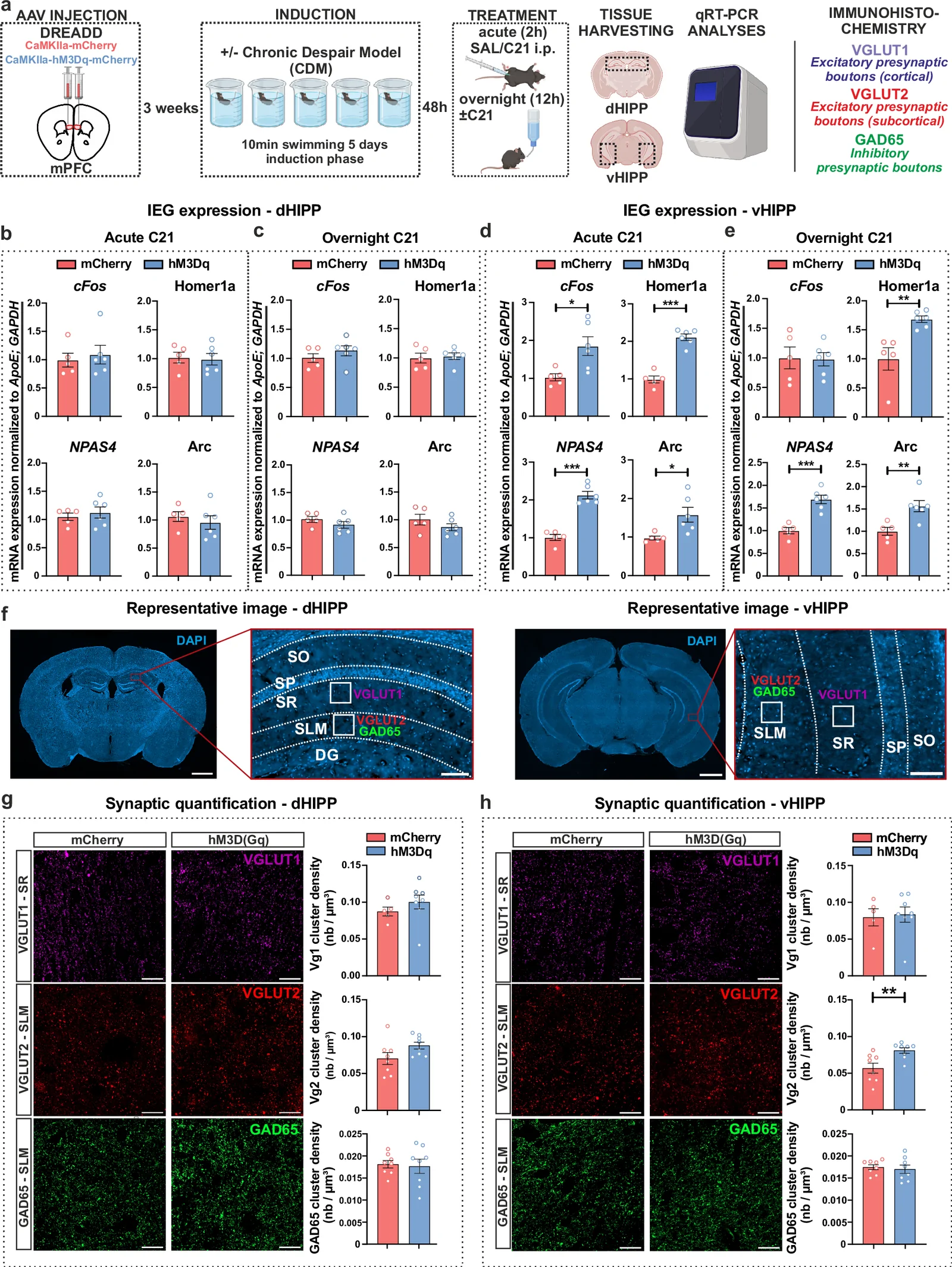
Prefrontal IL DREADD activation differentially regulates immediate early genes and structural synaptic plasticity across hippocampal subregions. **a** Experimental design for IL DREADD activation, CDM induction, acute or overnight C21 treatment, and tissue collection for qRT-PCR and immunohistochemistry. **b–e** mRNA expression of immediate early genes (normalized to ApoE and GAPDH) of hM3Dq (*n* = 6) and mCherry (*n* = 5) mice in dorsal HIPP after acute C21 treatment (*cFos P* = 0.6627, *Homer1a P* = 0.8339, *NPAS4 P* = 0.5610, *Arc P* = 0.5108) (**b**) and following overnight C21 treatment (*cFos P* = 0.3050, *Homer1a P* = 0.7624, *NPAS4 P* = 0.2536, *Arc P* = 0.2482) (**c**) and in ventral HIPP following acute C21 treatment (*cFos P* = 0.0184, *Homer1a P* < 0.0001, *NPAS4 P* < 0.0001, *Arc P* = 0.0243, two-sided Welch’s *t*-test) (**d**) and after overnight C21 treatment (*cFos P* = 0.9172, *Homer1a P* = 0.0043, two-sided Mann-Whitney test, *NPAS4 P* = 0.0003, *Arc P* = 0.0064) (**e**). **f** Representative microscopy image of dorsal (left) and ventral (right) hippocampus. Scale bar: 1000 µm. Insets show magnified images of hippocampal layers where synaptic density was analysed. VGLUT1-positive clusters were quantified in Stratum Radiatum (SR), while VGLUT2 and GAD65-positive clusters were quantified in Stratum Lacunosum-Moleculare (SLM). SO: stratum oriens; SP: stratum pyramidale; DG: Dentate Gyrus. Scale bar 100 µm. **g** Synaptic density analysis in CA1 dHIPP layers. Left: representative confocal images showing VGLUT1 (magenta), VGLUT2 (red) and GAD65 (green) immunostaining in SR and SLM of mCherry and hM3Dq mice after overnight C21 treatment. Scale bar: 20 µm. Right: Quantification of VGLUT1, VGLUT2 and GAD65 cluster density in hM3Dq and control mice (VGLUT1: mCherry *n* = 5, hM3Dq *n* = 8, *P* = 0.1362; VGLUT2: *n* = 8, *P* = 0.0809; GAD65: *n* = 8, *P* = 0.8055). **h** Synaptic density analysis in CA1 vHIPP layers. Left: representative confocal images. Scale bar: 20 μm. Right: Quantification of presynaptic markers in hM3Dq and control mice treated with C21 overnight (VGLUT2: *n* = 8, *P* = 0.0081; GAD65: n = 8, *P* = 0.6843; VGLUT1: mCherry *n* = 5, hM3Dq *n* = 8, *P* = 0.3278). Unless otherwise stated, statistical comparisons were performed using unpaired two-tailed Student’s t-test. Data are presented as mean ± SEM with individual data points shown. *P < 0.05, **P < 0.01, ***P < 0.001. See also Supplementary Figs. 6 and 7. Source data are provided as a Source Data file. Created in BioRender. Vestring, S. (2026) https://BioRender.com/rpcq0ko.

The dorsal (dHIPP) and the vHIPP regions are functionally distinct, and are classically linked to cognition and emotion, respectively^39^. To assess whether IL stimulation differentially regulates these two compartments, we measured the mRNA expression of neuronal activation and plasticity-related IEGs in the dHIPP and vHIPP after CDM induction and IL DREADD activation (Fig. 4a). Acute i.p. administration of C21 upregulated *cFos*, *Homer1a, NPAS4* and Arc in the ventral area but remained unchanged in the dorsal area (Fig. 4b, d). After a prolonged overnight stimulation, the expression of all the IEGs except *cFos* was increased in the vHIPP; however, no changes were observed in the dHIPP (Fig. 4c, e and Supplementary Fig. 6f-h).

Additionally, acute DREADD activation in the IL led to upregulated *cFos*, Homer1a and *NPAS4* expression but not *Arc* levels in the mPFC (Supplementary Fig. 6a). Prolonged stimulation using C21 (0.75 mg/kg) administered via the drinking water overnight upregulated the expression of all the measured IEGs (Supplementary Fig. 6b). In contrast, acute C21 administration to control mice without the DREADD construct had no significant off-target effects on any of the investigated genes or regions (Supplementary Fig. 6c-e). These data suggest that IL stimulation increases neuronal activity and the expression of plasticity-related genes in the mPFC and in the ventral, but not dorsal HIPP, identifying this hippocampal subregion as a key downstream target of IL activation.

### IL stimulation increases ventral, but not dorsal hippocampal CA1 structural plasticity

To assess the effects of IL stimulation on structural plasticity, which is another key feature that is altered in depression^7,14,17^, we labeled input-specific presynaptic markers by immunohistochemistry (Fig. 4a, f). Then, we conducted three-dimensional quantification of synaptic density and volume in the CA1 region of both the ventral and dorsal HIPP. We used three distinct markers: GAD65, which is a marker of inhibitory presynaptic boutons in the stratum lacunosum moleculare (SLM), and VGLUT1 and VGLUT2, which are markers of excitatory presynaptic boutons in the stratum radiatum (SR) and SLM, respectively. These latter markers distinguish between intrinsic hippocampal or cortical inputs (VGLUT1) and subcortical/thalamic inputs (VGLUT2)^40,41^. After overnight IL stimulation, the GAD65 and VGLUT1 densities remained unchanged, but the VGLUT2 puncta density significantly increased in the vHIPP but not in the dHIPP SLM (Fig. 4g, h); suggesting a selective increase of subcortical inputs. In the mPFC, VGLUT2 and GAD65 density remained unchanged after overnight IL stimulation (Supplementary Fig. 7a, b). Finally, no change in the mean volume of VGLUT1, VGLUT2, or GAD65 puncta was observed in the dorsal or ventral HIPP (Supplementary Fig. 7e-g).

Taken together, these results show that IL preferentially increases vHIPP excitatory synapses from subcortical origins.

### The RE gates IL-stimulation mediated antidepressant effects

**Fig. 5:**
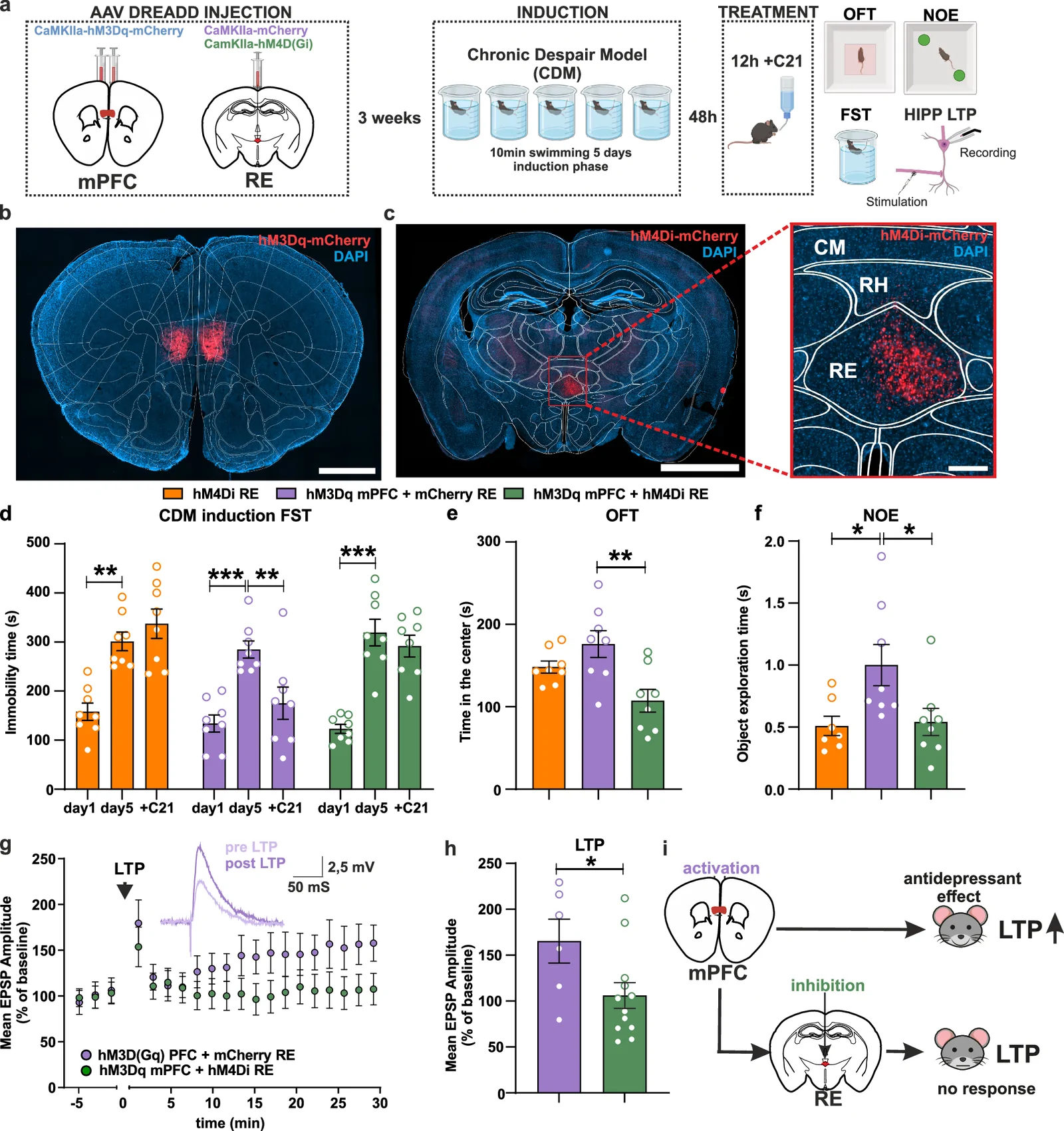
The RE gates IL-stimulation mediated antidepressant effects. **a** Experimental design of the viral strategy for IL activation and RE inhibition, CDM induction, overnight C21 treatment, and behavioral or ex vivo LTP recordings. **b** Confocal image of the IL injection (scale bar: 1000 μm; inset 100μm). Representative of n = 9 mice, see Supplementary Fig. 1c. **c** Confocal image of a RE injection (scale bar: 1000 μm; inset 100μm). Representative of *n* = 7 mice, see Supplementary Fig. 1h. **d** Immobility time in swim test on day 1, day 5 of CDM, and after C21 treatment across experimental groups. hM3Dq mPFC + mCherry RE (*n* = 8): day 1 vs day 5 *P* < 0.0001; day 5 vs +C21 P = 0.0095. hM4Di RE (*n* = 8): day 1 vs day 5 P = 0.0081; day 5 vs +C21 *P* = 0.3233. hM3Dq mPFC + hM4Di RE (*n* = 8): day 1 vs day 5 P = 0.0008; day 5 vs +C21 *P* = 0.0864. Repeated measures two-way ANOVA with Bonferroni post-hoc test. **e** Time spent in center in the OFT. hM3Dq mPFC + mCherry RE vs hM3Dq mPFC + hM4Di RE P = 0.0034; hM4Di RE vs hM3Dq mPFC + mCherry RE *P* = 0.0903; hM3Dq mPFC + hM4Di RE vs hM4Di RE *P* = 0.3077. One-way ANOVA with Tukey’s post-hoc test. **f** Novel object exploration time normalized to hM3Dq mPFC + mCherry RE group. hM3Dq mPFC + mCherry RE vs hM3Dq mPFC + hM4Di RE P = 0.0417; hM4Di RE vs hM3Dq mPFC + mCherry RE *P* = 0.0350; hM3Dq mPFC + hM4Di RE vs hM4Di RE *P* = 0.9837. One-way ANOVA with Tukey’s post-hoc test. **g** Time course of aLTP measurements (hM3Dq mPFC + mCherry RE *n* = 6 cells; hM3Dq mPFC + hM4Di RE n = 12 cells) with exemplary EPSP traces. **h** Group analysis of aLTP measurements of hM3Dq mPFC + mCherry RE (*n* = 6 cells) and hM3Dq mPFC + hM4Di RE (n = 12 cells) (*P* = 0.0372, two-sided unpaired Student’s *t*-test). **i** Schematic summary: IL activation produces an antidepressant effect and restores hippocampal LTP, both abolished by RE inhibition. Data are presented as mean ± SEM with individual data points shown. *P < 0.05, **P < 0.01, ***P < 0.001. See also Supplementary Fig. 8. Source data are provided as a Source Data file. Created in BioRender. Vestring, S. (2026) https://BioRender.com/rpcq0ko.

To investigate the circuit-related mechanisms mediating the effects of the IL on the HIPP, we focused on the RE, which is a key relay structure that coordinates PFC–HIPP communication^42,43^. Via a dual-chemogenetic approach, we simultaneously activated the IL while inhibiting the RE (Fig. 5a-c, Supplementary Fig. 1f-h). After CDM induction, IL DREADD stimulation with the control construct in the RE (CaMKIIα-hM3Dq in the IL + CaMKIIα-mCherry in the RE) resulted in antidepressant-like effects in the FST (Fig. 5d). However, concurrent RE inhibition (CaMKIIα-hM3Dq in the IL + CaMKIIα-hM4Di in the RE) abolished these effects. RE inhibition alone had no effect on immobility time, indicating that the RE plays a specific role in mediating IL-driven effects (Fig. 5d). Additional behavioral tests revealed similar patterns: while mice expressing hM3Dq in the IL and mCherry in the RE spent increased time in the center during the OFT and increased time exploring the novel object, these effects were abolished by RE inhibition (Fig. 5e, f).

To determine whether the RE also mediates the effects of the IL on associative plasticity in the HIPP, we performed patch‒clamp recordings of ex vivo HIPP slices from CDM mice expressing the same viral constructs. Following our established protocol, we induced aLTP via paired Schaffer collateral-postsynaptic stimulation. While IL stimulation (CaMKIIα-hM3Dq) with the control virus in the RE (CaMKIIα-mCherry) successfully restored aLTP, which is consistent with our results above, concurrent RE inhibition (CaMKIIα-hM4Di) prevented this restoration, maintaining the CDM-associated plasticity deficit (Fig. 5g, h), with no changes in membrane resistance or paired-pulse ratio (Supplementary Fig. 8a-d). IL stimulation did not alter VGLUT1 or GAD65 puncta density or volume within the RE itself (Supplementary Fig. 7c, d).

These results determine the RE as an essential mediator of both the behavioral and plasticity-related effects of IL activation, revealing the critical role of the IL → RE → HIPP circuit in antidepressant response (Fig. 5i).

### The RE is required for ketamine-induced antidepressant effects and plasticity restoration

**Fig. 6:**
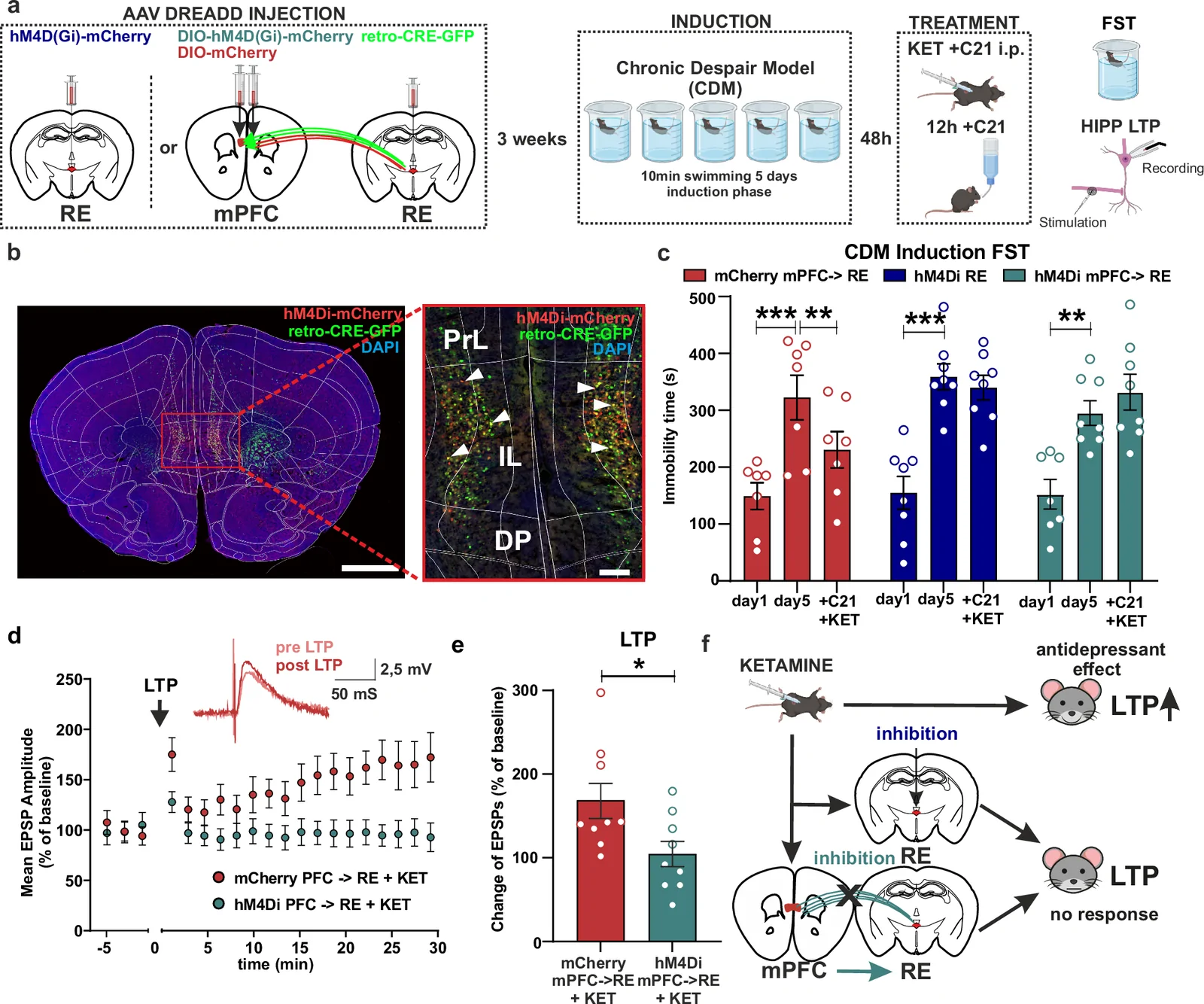
The IL → RE pathway is required for ketamine antidepressant effect and plasticity restoration. **a** Experimental design for intersectional IL → RE inhibition or RE inhibition, CDM induction, ketamine (KET) + C21 treatment, and FST or patch-clamp recordings. **b** Confocal image of the expression of retro-hSyn-Cre (green) and DIO-hM4Di-mCherry (red) in the IL. Scale bar: 1000 μm. Inset: higher magnification image showing co-labeled cells in the IL, with PrL and DP boundaries indicated. Scale bar: 100 μm. Representative of *n* = 5 mice, see Supplementary Fig. 1d. **c** Immobility time in swim test measured on day 1, day 5 of CDM, and after ketamine treatment. Ketamine + PFC → RE mCherry (*n* = 7): day 1 vs day 5 *P* = 0.0009; day 5 vs +C21 *P* = 0.0093. Ketamine + RE hM4Di (*n* = 8): day 1 vs day 5 *P* = 0.0005; day 5 vs +C21 *P* = 0.6987. Ketamine + PFC → RE hM4Di (*n* = 8): day 1 vs day 5 *P* = 0.0084; day 5 vs +C21 *P* = 0.5380. Repeated measures two-way ANOVA with Bonferroni post-hoc test. **d** Time course of aLTP inducibility of ketamine + mCherry PFC → RE and ketamine + hM4Di PFC → RE groups (*n* = 9 cells per group), with exemplary EPSP traces. **e** Group analysis (*n* = 9 cells per group, *P* = 0.0264, two-sided unpaired Student’s *t*-test). **f** Schematic summary of the results. Ketamine 10 mg/kg produces antidepressant effect and restores hippocampal aLTP. Chemogenetic blockade of the RE or PFC → RE projections abolishes both antidepressant effect and hippocampal aLTP restoration. Data are presented as mean ± SEM and the individual data points are depicted. *P < 0.05, **P < 0.01, ***P < 0.001. See also Supplementary Fig. 8. Source data are provided as a Source Data file. Created in BioRender. Vestring, S. (2026) https://BioRender.com/rpcq0ko.

To investigate the broader role of the RE in the antidepressant response, we examined its role in the therapeutic effect of ketamine. We used two approaches to inhibit RE function (Fig. 6a): direct local RE inactivation using inhibitory DREADDs (CaMKIIα-hM4Di) and selective inhibition of IL → RE projections using an intersectional strategy that combines retrograde AAV-Cre in the RE and Cre-dependent hM4Di in the IL (Fig. 6b; Supplementary Fig. 1d, f, h). Consistent with previous findings^16,28,32^, ketamine (10 mg/kg) ameliorated CDM-induced immobility in control non-AAV-injected mice (Supplementary Fig. 8i). However, both global RE inhibition (CaMKIIα-hM4Di in RE) and selective IL → RE projection inhibition (DIO-hM4Di in mPFC + retro-hSyn-CRE in RE) completely blocked this antidepressant response (Fig. 6c). Similarly, while ketamine restored associative LTP in CA1 pyramidal neurons in hippocampal slices from CDM mice (Supplementary Fig. 8j, k); IL → RE projection inhibition selectively prevented this restoration, while membrane resistance and paired-pulse ratio remained unchanged (Fig. 6d, e; Supplementary Fig. 8l-o), despite the well described local effects of ketamine on hippocampal circuits^28^.

These findings demonstrate that the RE is essential not only for IL stimulation-induced effects but also for the antidepressant and neuroplastic effects of ketamine (Fig. 6f).

### RE→vHIPP projections are required for IL-stimulation-induced and ketamine effects

**Fig. 7:**
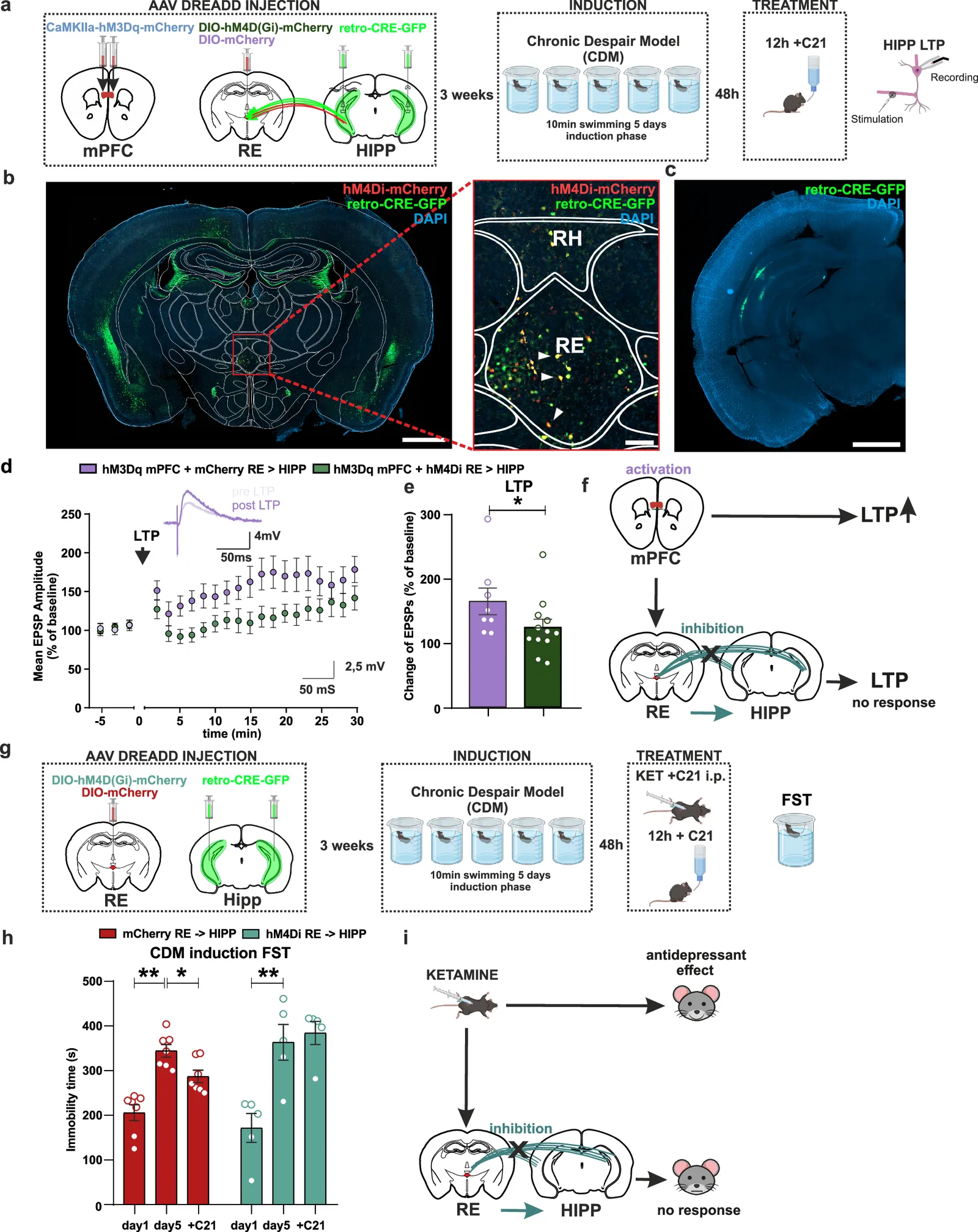
The RE→hippocampus projections are required for IL stimulation and ketamine effects. **a** Experimental design for DREADD targeting of the IL (activation) and RE→hippocampus projection (inhibition), CDM induction, overnight C21 treatment, and patch-clamp recordings. **b** Left: confocal image of retro-CRE-GFP and DIO-hM4Di expression in the RE, overlaid on the corresponding Allen Brain Atlas slice. Scale bar 1000 µm. Inset: higher magnification image showing co-labeled cell in the RE and RH. Scale bar 100 µm. Representative of *n* = 4 mice, see Supplementary Fig. 1i. **c** Confocal image of retro-CRE-GFP expression in the vHIPP. Scale bar 1000 µm. Representative of n = 4 mice. **d** Time course of aLTP measurements comparing hM3Dq mPFC + mCherry RE → HIPP (purple, n = 8 cells) and hM3Dq mPFC + hM4Di RE → HIPP (dark green, *n* = 12 cells) groups, with exemplary EPSP traces. **e** Group analysis of aLTP measurements (mCherry RE → HIPP: n = 8 cells; hM4Di RE → HIPP: *n* = 12 cells; *P* = 0.0473, two-sided Mann–Whitney test). **f** Schematic summary: IL stimulation restores hippocampal aLTP; chemogenetic inhibition of RE → HIPP projections blocks this restoration. **g** Schematic overview of the experimental design for behavioral experiments. Stereotactic delivery of retro-Cre-GFP in the ventral hippocampus and DIO-hM4D(Gi)-mCherry or DIO-mCherry in the RE. After CDM, mice received ketamine (10 mg/kg) + C21 i.p. and C21 overnight, followed by FST. **h** Immobility time in swim test measured on day 1, day 5 of CDM, and after ketamine + C21 treatment. mCherry RE → HIPP + ketamine (*n* = 7 mice): CDM induction day 1 vs day 5 *P* = 0.0045; day 5 vs post-treatment *P* = 0.0289. hM4Di RE → HIPP + ketamine (*n* = 5 mice): CDM induction day 1 vs day 5 *P* = 0.0012; day 5 vs post-treatment *P* = 0.6726. Repeated measures two-way ANOVA with Bonferroni post-hoc test. **i** Schematic summary: ketamine produces antidepressant effects; chemogenetic inhibition of RE → HIPP projections blocks the behavioral response to ketamine. Data are presented as mean ± SEM and the individual data points are depicted. *P < 0.05, **P < 0.01, ***P < 0.001. See also Supplementary Fig. 8. Source data are provided as a Source Data file. Created in BioRender. Vestring, S. (2026) https://BioRender.com/rpcq0ko.

To directly test whether the final segment of the circuit, i.e., RE projections to the vHIPP, is required for antidepressant effects, we used an intersectional approach combining retrograde AAV-Cre in the vHIPP with Cre-dependent hM4Di or mCherry in the RE (Fig. 7a-c; Supplementary Fig. 1i). In CDM mice expressing hM3Dq in the IL, overnight C21 treatment restored aLTP when RE→vHIPP neurons expressed the control construct (mCherry), but this restoration was blocked when RE→vHIPP neurons were inhibited (hM4Di; Fig. 7d, e). Membrane resistance remained unchanged, and paired-pulse ratio was unaffected (Supplementary Fig. 8e-h), consistent with a predominantly postsynaptic effect and in line with effects observed above (Fig. 5g, h; Fig. 7f).

**Fig. 8:**
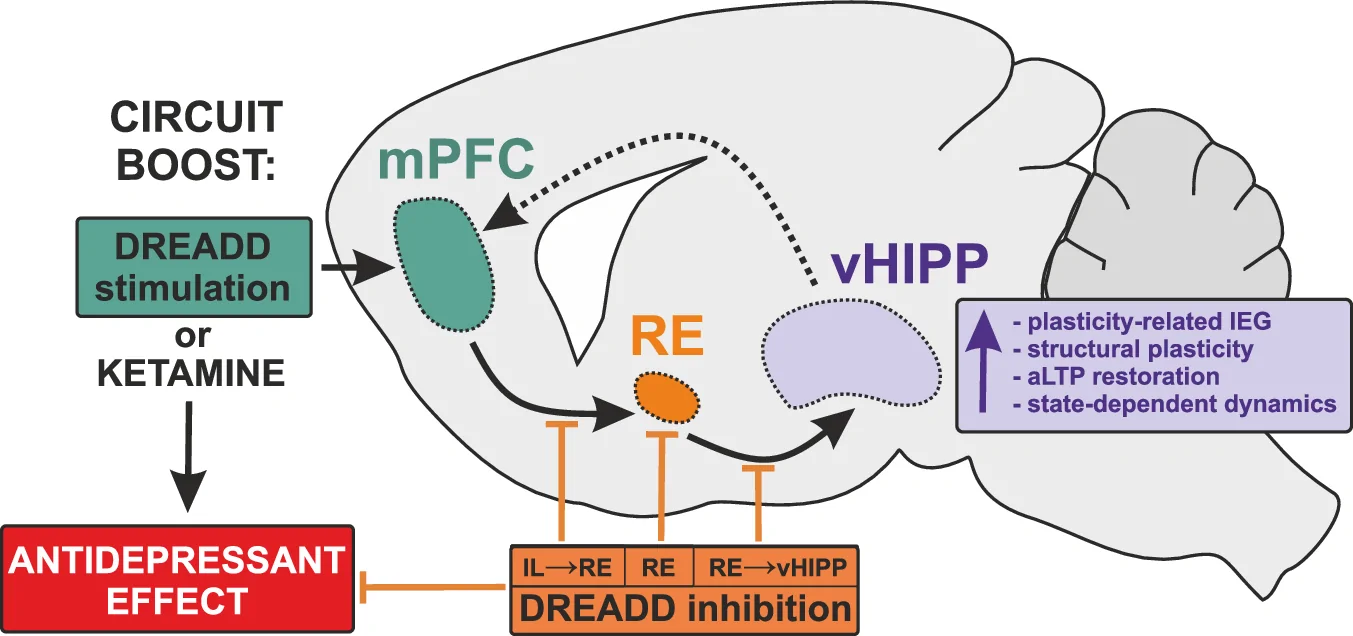
Proposed model for the role of the IL→RE→vHIPP→PFC loop in antidepressant response. In a depressed-like state, the circuit is impaired, structural plasticity is reduced, and hippocampal aLTP is abolished. Antidepressant treatment using either ketamine or IL DREADD stimulation activates the circuit, restores normal behavior, enhances structural plasticity and hippocampal state-dependent dynamics, and rescues aLTP. Conversely, disruption of this loop through the inhibition of mPFC→RE or RE→vHIPP projections prevents the restoration of the circuit, blocking the antidepressant-like effects and the rescue of hippocampal aLTP.

To evaluate if this circuit segment is also implicated in mediating antidepressant effect, we tested whether RE→vHIPP projections are also required for the behavioral effects of ketamine. While ketamine ameliorated CDM-induced immobility in mice expressing mCherry in RE→vHIPP neurons, this effect was abolished by concurrent hM4Di-mediated inhibition of RE→vHIPP projections (Fig. 7g-i). Together, these results demonstrate that RE→vHIPP projections are required for both IL stimulation-induced plasticity restoration and ketamine’s antidepressant effects, establishing each segment of the IL → RE→vHIPP circuit as necessary for antidepressant response (Fig. 8).

Finally, to assess whether the IL → RE→vHIPP circuit shows functional dysregulation in vivo, we performed longitudinal local field potential (LFP) recordings simultaneously in the mPFC, RE, and vHIPP across naïve, post-CDM, and C21-treated conditions (Supplementary Fig. 9a-l). CDM altered inter-regional oscillatory coupling across the circuit, notably reducing mPFC–vHIPP low-γ coherence during locomotion (Supplementary Fig. 9o) and disrupting mPFC–RE and vHIPP-RE theta-to-gamma phase-amplitude coupling during novel object exploration (Supplementary Fig. 9p, q). Acute IL activation partially restored these effects (Supplementary Fig. 9m-r).

## Discussion

Depression is a complex disorder that is characterized by a wide range of symptoms and the involvement of multiple, likely overlapping, biological processes, including molecular, circuit-related, and electrophysiological mechanisms. Despite this heterogeneity, studies on diverse patient cohorts and animal models have highlighted a significant degree of convergence in several pathophysiological features. Among these features, deficits in neuroplasticity, including associative plasticity deficits and excitatory synapse downregulation in the PFC and HIPP, have emerged as consistent findings across clinical and preclinical studies. These synaptic deficits are accompanied by structural changes, such as dendritic spine loss and altered synaptic density, and these changes can be targeted by treatment with fast-acting antidepressants^7,17,44,45^. Another key feature that has been consistently reported across studies involves changes in specific brain circuits, and robust evidence has indicated that PFC and HIPP abnormalities are major factors that contribute to the pathophysiology of depression^22,46^.

In this study, we identified a circuit that connects these two pivotal brain regions via the RE of the thalamus. This circuit not only drives antidepressant effects but also controls hippocampal neuroplasticity and microcircuit dynamics. Notably, activation of the IL alone was sufficient to induce antidepressant-like effects in several behavioral domains. This circuit specificity is highlighted by our finding that PrL stimulation did not elicit antidepressant-like effects, which is consistent with previous results showing opposing roles of the IL and PrL in emotional regulation^47^.

Our results demonstrate that the effects of IL stimulation extend to the HIPP. HIPP atrophy^10,48,49^, decreased cell size^50^, reduced CA1 volume^51^ and synaptic loss have been associated with different stages of the disease. IL stimulation induced region-specific molecular changes in the HIPP, leading to the upregulation of IEGs such as *cFos*, *Homer1a*, and *NPAS4* in the ventral but not the dorsal HIPP. They act as markers of neuronal activation and synaptic plasticity, and their upregulation indicated that IL stimulation increased neuronal activity in these regions. Indeed, c-Fos is a transcription factor that directly influences synaptic plasticity and has been associated with mood disorders^52^. Notably, Homer1a regulates glutamate receptor function and intracellular calcium signaling, and its expression is affected by stress and antidepressants^16,31^. NPAS4 balances excitatory and inhibitory synapses, which is crucial for emotional stability, and its dysregulation is associated with depressive-like behaviors^53^; Arc modulates synaptic strength by directly regulating AMPA receptor trafficking, and its dysfunction has been associated with cognitive deficits and mood disorders^54^.

To investigate circuit-specific synaptic changes, we studied input-specific presynaptic markers. VGLUT1 and VGLUT2 show complementary distribution patterns in the brain, with VGLUT1 being predominantly expressed in cortical regions and intrinsic hippocampal circuits whereas VGLUT2 is expressed mainly in subcortical projections, including those from the supramammillary area and the RE^40,41,55^. The distribution of VGLUT2 is consistent with its presence mainly in the SLM of the CA1 region, which is a major zone of thalamic termination^56,57^. Here, the specific increase in VGLUT2 expression in the ventral, but not dorsal CA1 SLM therefore suggests an increase in connections arising from a thalamic (likely RE) origin. Notably, VGLUT2 and GAD65 density remained unchanged in the mPFC after IL stimulation, indicating that thalamic inputs to the mPFC are not detectably remodeled and that the synaptic effects of IL stimulation are directed downstream in the IL → RE→vHIPP pathway rather than locally within the prefrontal cortex. This directionality is consistent with the known anatomy of this circuit. The mPFC does not send direct excitatory projections to the HIPP; its primary excitatory route is relayed through the RE, which preferentially innervates intermediate and ventral CA1 and the subiculum with comparatively sparse projections to the dHIPP^56,58^. Although a recent study identified a direct monosynaptic mPFC→HIPP connection, this projection is inhibitory, sparse, and was characterized only between the mPFC and the dHIPP^59^.

The RE therefore constitutes the privileged excitatory relay linking the PFC to the vHIPP, consistent with our functional data demonstrating that both inhibition of IL → RE (Fig. 6) and RE → HIPP projections (Fig. 7) abolish the effects of IL stimulation. Furthermore, the vHIPP projects back to the mPFC^60^, suggesting the presence of a functional loop (Fig. 8). This regional specificity is functionally relevant, as the vHIPP has been implicated in emotional processing and stress responses, whereas the dHIPP is more involved in spatial cognition and memory^39^. The selective engagement of the vHIPP through this circuit is therefore consistent with the behavioral profile observed in our study.

Our results directly link circuit-level analysis with neuroplasticity, which is another key mechanism that has been implicated in responses to antidepressants^7,27^. We provide compelling evidence that chronic stress-induced impairments in hippocampal LTP can be fully reversed by stimulation of the IL (Fig. 2a-f). This restoration of plasticity reproduces the effects of ketamine or long-term selective serotonin reuptake inhibitor treatment^28,61^, which is considered a crucial criterion for antidepressant response^62^.

Additionally, we used in vivo fiber photometry to directly monitor vHIPP activity during TST, and we found that IL stimulation bidirectionally increased the dynamic range of vHIPP responses between active and passive coping behavioral states^63^ (Fig. 3a-g), potentially influencing the processing of salient events. A recent theoretical framework proposes that the vHIPP is a hub that links external stimuli to internal emotional and motivational drives^64^. The vHIPP has been shown to encode the anxiogenic features of an environment to enable appropriate defensive responses^65^, and it is implicated in social encoding^66^ and susceptibility to stress and depression^67^. Furthermore, increased excitability and activity-dependent processes in the dentate gyrus have been associated with antidepressant-like effects^46^. Concurrent with increased HIPP plasticity, our findings suggest that IL stimulation increases HIPP processing and could participate in the modulation of emotional processing during antidepressant therapy.

The RE is a key node that synchronizes mPFC-HIPP activity^68^, supporting the flow of information between these structures. It receives dense excitatory projections from the IL that shape its output: both chemogenetic IL activation and manipulation of IL → RE terminals modulate RE firing patterns^63^. Functionally, the RE plays a crucial role in aversive memory specificity and extinction. RE inhibition leads to persistent memories of fear after stimulus removal^69^ and maladaptive generalization of fear to unrelated stimuli^70^ and RE neurons also show altered physiology in disease models associated with hippocampal–thalamo–cortical network dysfunction^71,72^. Despite its established roles in memory processing, the involvement of the RE in the response to antidepressants remains largely unexplored. While one study reported that RE lesions exert antidepressant-like effects^73^, the use of the FST in two sessions in that study—which cannot exclude the involvement of learning processes—raises a critical concern about the interpretation of the results. Further studies are needed to elucidate the role of the RE in resilience and the establishment of a depressive-like phenotype.

Our results following RE inhibition strongly suggest that the antidepressant effects of IL stimulation and the restoration of HIPP plasticity are mediated by the IL → RE → HIPP pathway. The RE targets the HIPP at the SLM of the CA1 region, which coincides with our observation of increased VGLUT2 synaptic density (Fig. 4a-h) and suggests that IL stimulation-induced structural plasticity is conveyed, at least in part, through increased RE synapses. The RE targets both excitatory and inhibitory neurons in the HIPP; however, the precise nature of its inhibitory targets is yet to be fully understood^57,74^.

We found that inhibition of either the RE, IL → RE, or RE→vHIPP projections abolishes both the antidepressant effects of ketamine and the restoration of hippocampal LTP (Fig. 6a–f; Fig. 7a–i). This finding is striking, given that the effect of ketamine on LTP is mediated (at least in part) through its direct effects on the HIPP, particularly through the disinhibition of pyramidal neurons^28,61,75^. One possibility is that while ketamine acts acutely to restore hippocampal LTP through local mechanisms, the maintenance of this effect requires the sustained engagement of a broader IL → RE → HIPP → mPFC loop. Indeed, this ascending pathway projects back to the PFC, forming a loop that is important for hippocampal‒prefrontal synchrony, which is critical for memory and emotional processing^76^. Alternatively, ketamine could act at multiple points and/or particularly on mPFC, to activate this loop, and this activation might be necessary to exert antidepressant effects and restore plasticity in the HIPP. For example, local infusion of ketamine into the mPFC is sufficient to produce antidepressant-like effects, and this effect can be mimicked by optogenetic IL stimulation and abolished by IL inhibition^77^. Inhibition of any element of this chain would prevent the activation of the loop and therefore disrupt the effect, abolishing both the antidepressant effects and neuroplastic benefits of mPFC stimulation or ketamine. This circuit-based interpretation is supported by previous work that showed that activation of vHIPP → mPFC projections not only reproduces but also is necessary for the antidepressant effects of ketamine^78^ and that vHIPP inputs preferentially target RE-projecting IL neurons^79^, thereby closing this loop driving antidepressant response and controlling hippocampal plasticity.

Long-range coherence and theta–gamma coupling have been linked to inter-regional information transfer, while theta phase timing is thought to define temporal windows favorable for synaptic plasticity^80^. Our in vivo LFP recordings show that CDM is associated with impaired inter-regional oscillatory coordination within the IL → RE→vHIPP circuit, and that IL activation partially reverses these abnormalities, consistent with a role for circuit-level oscillatory dynamics in the modulation of hippocampal plasticity.

This circuit may not be the only network underlying depression. Other circuits, such as those involving the lateral habenula (LHb), NAc, VTA or BLA, are thought to be implicated in depression pathophysiology and treatment^67,81–86^. The IL → RE → vHIPP circuit is strongly connected to these structures, and the mPFC plays a putative role in the top-down control of these structures^67,86^. Given that IL stimulation affects motivational, social, and anhedonia-like behaviors, which are highly dependent on limbic structures, our findings suggest that stimulation of this circuit might be a central starting point for antidepressant effects, potentially spreading to multiple downstream targets. Notably, each node of the IL → RE→vHIPP circuit sends dense projections to the BLA^19,87,88^, providing at least three distinct entry points through which the IL → RE→vHIPP circuit could influence amygdala function, suggesting a complex and interconnected involvement of the BLA in these processes.

Recent theories posit that neuroplasticity provides neural networks with the ability to adapt to intrinsic and extrinsic stimuli^27,89^. While the initial suppression of plasticity under stress conditions might be a protective mechanism that reestablishes homeostatic balance and prevents excessive impacts of a repeatedly negative environment^90^, prolonged plasticity disruption can become maladaptive and contribute to psychiatric disorders such as depression^45^. LTP measurements in hippocampal slices provide a rather binary readout of associative plasticity: present in healthy condition, it is abolished in a pathological state and restored after antidepressant treatment. Our manipulations of the IL → RE → HIPP circuit reflect this binary nature, as activating this circuit turns on hippocampal LTP while disrupting it prevents this effect, maintaining LTP in an off state. Therefore, we hypothesize that this circuit might provide a pathway to alleviate the maladaptive inhibition of LTP that is observed in depression, analogous to resetting a safety switch.

Altogether, our study unifies two prominent theoretical frameworks for understanding depression pathophysiology, namely, the circuitry hypothesis and the neuroplasticity hypothesis. We identified the IL → RE → vHIPP circuit as a central and converging network that regulates the response to antidepressant therapy and plasticity in the hippocampus. Targeted stimulation of this circuit might facilitate potent antidepressant benefits while avoiding off-target effects on undesirable circuits, which is a plausible cause of the adverse effects of pharmacotherapies.

Converging evidence from human studies supports the translational relevance of the IL → RE → vHIPP circuit. The human homolog of the rodent IL, the subcallosal cingulate cortex (SCC, BA25), is a central target in DBS for treatment-resistant depression^91^. Diffusion tensor imaging (DTI) based tractography in SCC DBS patients has identified three critical white matter bundles that are necessary for clinical antidepressant response, including descending subcortical midline fibers projecting to the anterior midline thalamus^92^. Anatomical tracing studies in non-human primates have confirmed that the RE has strong reciprocal connections with the SCC homolog and constitutes the primary thalamic input to the HIPP, with the capacity to simultaneously excite neurons in both CA1 and the mPFC^93^. PET imaging further demonstrates that SCC-DBS modulates activity not only locally but also in downstream thalamic and hippocampal nodes^91^, and effective connectivity analyses have identified path-level abnormalities within the SCC–anterior thalamus–HIPP circuit that distinguish treatment responders from non-responders^94^. Together, these findings provide human connectomic evidence for a SCC → RE → vHIPP pathway homologous to the circuit described here, and its involvement in antidepressant mechanisms.

Recent developments in brain stimulation techniques, such as DBS, TMS and transcranial focus ultrasound stimulation, have provided unique opportunities for the targeted treatment of neuropsychiatric disorders^9,18,95,96^; however, the effectiveness of these approaches is currently limited by an incomplete understanding of the brain circuits involved. Further studies of the IL → RE → vHIPP network could contribute to addressing the urgent need for novel strategies for the treatment of depression.

## Methods

### Animals and Husbandry

Wild-type C57BL/6 J mice (Mus musculus; RRID: IMSR_JAX:000664) were obtained from Charles River or Janvier (France and Germany) and maintained at the animal husbandry facilities of the Universities of Freiburg (Center for Experimental Models and Transgenic Service) and Strasbourg (Chronobiotron). The Chronobiotron facility is registered for animal experimentation (Agreement A67-2018-38). All the procedures were performed in accordance with the German animal protection law (TierSchG), FELASA, the guide for the care and use of laboratory animals of the national animal welfare body GV-SOLAS and the EU Directive 2010/63/EU for animal experiments. Moreover, the procedures were approved by animal review boards in Germany (Regierungspräsidium Freiburg, licenses G-19/44, G-20/141, G-22/116, G-24/033 and G-25/032) or France (CREMEAS, APAFIS n°2020042818477700), and all the animal care protocols followed national and international standards, with consistent documentation of procedures. Power calculations were conducted before experiments to assess animal behavior to minimize the number of animals used. Both male and female mice were included throughout the study. All the mice were at least 8 weeks old at the start of experimental procedures and were housed in a temperature- (21 ± 2 °C) and humidity- (55 ± 10%) controlled environment, with ad libitum access to food and water, under a 12-h light–dark cycle.

### Surgical procedures

Before surgery, the mice received subcutaneous injections of carprofen (4 mg/kg) and buprenorphine (0.1 mg/kg). Mice were anesthetized with isoflurane (4% for induction, 1.5% for maintenance) and positioned in a stereotaxic frame (51730UD, Stoelting, USA). Ophthalmic gel (Ocrygel, TVM, UK) was applied to prevent corneal drying, and body temperature was maintained using a heated pad. A total of 300 nL of viral suspension, namely, a suspension of ssAAV-5/2-mCaMKIIα-hM3D(Gq)_mCherry-WPRE-hGHp(A) (v96-5, Viral Vector Facility VVF, Switzerland), ssAAV-5/2-mCaMKIIα-mCherry-WPRE-hGHp(A) (v199-5, VVF, Switzerland), AAV5-hSyn-DIO-mCherry (v116-5, VVF, Switzerland) or AAV5-hsyn-DIO-hm3d(Gq)-mCherry (44361-AAV5, Addgene, USA), was bilaterally injected into the IL ( + 1.7 mm AP, +/−0.3 ML, −2.8 DV relative to Bregma) or PrL ( + 1.7 AP, +/−0.3 ML, −2.4 DV) regions of the PFC of WT mice using a 30 G syringe (Hamilton Neuros syringe) and a microinjection syringe pump (UMP3T, WPI, USA) at a rate of 200 nL/min. For the RE injections, a similar process was followed to unilaterally inject ssAAV-5/2-mCaMKIIα-hM4d(Gi)_mCherry-WPRE-hGHp(A) (v102-5, VVF, Switzerland), ssAAV-5/2-mCaMKIIα-mCherry-WPRE-hGHp(A) (v199-5, VVF, Switzerland), or AAV(retro).hSyn.HI.eGFP-CRE.WPRESV40 (105540-AAVrg, Addgene, USA) at a 15° angle ( + 1.7 AP, −1.1 ML, −4.5DV). After injection, the syringe was held in place for five minutes before being slowly withdrawn. pAAV-hSyn-DIO-hM3D(Gq)-mCherry was a gift from Bryan Roth, pENN.AAV.hSyn.HI.eGFP-Cre.WPRE.SV40 was a gift from James M. Wilson.

For fiber photometry, a genetically encoded calcium indicator (GeCI) was unilaterally injected in the vHIPP, and the left and right side were randomized. For inhibitory neurons, AAV5-mDlx-HBB-chl-NES_jRGECO1a-WPRE-SV40a (v527-5, VVF, Switzerland) was injected to record inhibitory neurons (−3.4 AP, −3 ML, −4 DV) and AAV5-mCaMKIIα-jGCaMP8m-WPRE-bGHp (v630-5, VVF, Switzerland) was injected to record excitatory neurons (−3.4 AP, −3.4 ML, −4 DV). The mice were subsequently implanted with a ferrule-coupled optical fiber (400 µm core diameter, 0.37 NA, MFC_400/470-0.37_4.5 mm_MF2.5_FLT, Doric Lenses, Canada) above the injection site. The implant was stabilized with three screws around it and secured with dental cement (Light Curing Nano Flowable Composite, Dline, Lithuania), which was solidified using a UV-light-emitting device. The mice received 0.1 mg/kg buprenorphine 6 h postsurgery and carprofen (4 mg/kg, s.c.) every 12 h for 3-5 days. The mice were allowed to recover for 3 weeks before experimental procedures.

### Behavioral procedures

Chronic despair model (CDM): The CDM model is an established mouse model of depression-like behavior and reliably predicts the effects of antidepressants^30–32,97^. To establish this model, mice were forced to swim in a glass cylinder (Ø 15 cm, 27 cm high) filled with water (21–23 °C) to a depth of 20 cm for 10 min on 5 consecutive days (induction phase). The immobility time was measured in each session. Mice were considered immobile when they floated in an upright position and made only minimal movements to keep their head above the water.

Immobility from Day 1 to Day 5 is tracked to verify successful induction of depression-like behavior at the cohort level. After swimming, the animals were gently dried and kept in a cage under a heating lamp for an additional ten minutes. Then, the mice were allowed to rest for two days in their home cages before the readout experiment was performed on the third day after the induction phase. Outcome measures (forced swim test, tail suspension test, or sucrose preference test) are then performed in distinct cohorts, in order to avoid additive confounds.

DREADD activation: Compound C21 (#5548, Tocris, United Kingdom) was dissolved in the drinking water of the mice, and this water was given to the mice overnight (6 p.m to 8 a.m). The dose was calculated considering an average consumption of 5 mL per mouse per night.

Open field test (OFT): The test was performed in a square arena (50 × 50 cm) with a virtual square area of 30 × 30 cm in the center. The open field was surrounded by a 35-cm high wall made of gray PVC. The mice were placed in the center of the field and allowed to move freely. Behavior was recorded for 10 min using IC Capture 4.0 (The Imaging Source, Germany), and total distance traveled and time spent in the center area were analyzed using Ethovision XT (Noldus, Netherlands).

Tail suspension task: For the TST, mice were attached via their tails (1–1.5 cm from the tip of the tail) to a horizontal bar located 40 cm above a table. Each trial was conducted for 6 min, and immobility time was determined.

Novel object exploration: Mice were allowed to explore an open field (50 × 50 cm) for 10 min. Then, two previously unseen objects (maximum size of 10 × 10 × 10 cm) were added two outer edges of the space. Behavior was recorded for 10 min, and then, the video was stopped and the mouse was returned to its cage. Post hoc analysis of the explorations was performed using Ethovision XT (Noldus, Netherlands).

Three chamber task (3ChT): A 3-chamber apparatus (60×25×23 cm, chambers of 20×25×23 cm) was used to record the sociability of the mice. The mice were able to explore the full apparatus for 10 min. Then, the external rooms were closed, and the mice were restricted in the central room. A previously unseen object and an unknown mouse that was restricted to a meshed cup were placed in the outer rooms. Then, the doors were opened, and interactions were recorded for 10 min. Interactions of the test mouse with the object or the social visitor were quantified using the Ethovision XT (Noldus, Netherlands) software.

Nosepoke sucrose preference test (nSPT): The IntelliCage system (TSE Systems, Germany), which allows automatic analysis of spontaneous and exploratory behavior and drinking preference of rodents, was used. Radiofrequency identification transponders were implanted in the mice. IntelliCage comprises a common space in the middle and four corners where measurements are recorded. Up to 16 group-housed mice can be given free access to food in the center, and water is supplied in the corners (two drinking bottles each) behind electronically managed doors. The corners can be visited by only one mouse at a time. Using IntelliCage Plus software (TSE Systems, Germany), the system records i) the number and duration of visits to the corners, ii) the number of times a mouse pokes a door with its nose (approaches), and iii) the number of times a mouse licked a drinking bottle. Initially, the mice were allowed to adapt to the IntelliCage for five days and provided free access to food and water in all the corners. Then, for three days, the animals were habituated to sucrose (each corner had one bottle that contained 1% sucrose solution and one bottle that contained water; the right/left positions of all the bottles were exchanged every day) and subjected to the nosepoke protocol. The mice had to poke a door with their nose to open it and had access to both water and sucrose solution. Next, an nSPT paradigm^16,32^ in which gradually increasing effort (number of times a mouse had to poke a door with its nose to open the door) was needed to access the bottles containing sucrose solution for a short period (24 h), was used to measure sucrose preference. In this protocol, each door was opened in response to a nosepoke and closed after the mouse drank from the sucrose solution-containing bottle for 5 s, but the mice were allowed to consume plain water for an unlimited amount of time. The number of nosepokes needed to access the bottle containing sucrose solution progressively increased (1, 2, 3, 4, 5, 6, and 7) after each of the 10 drinking sessions. This test was used to assess the drive to obtain a reward; a decreased sucrose preference indicated reduced reward behavior. For every side, the number of times a mouse licked a bottle was counted, and the mean sucrose preference was calculated as the percentage of times a mouse licked a bottle containing sucrose solution relative to the total number of times a mouse licked any bottle, pooled across all drinking sessions of the nose-poke paradigm.

### Quantitative real-time PCR (qRT‒PCR)

The mice were killed by cervical dislocation, and their brains were rapidly extracted and coronally sectioned. The regions of interest (dHIPP, vHIPP, or ventral medial prefrontal cortex tissue enriched for the IL) were microdissected, quickly frozen on dry ice and stored at −80°C until RNA was isolated. For the RNA extraction and expression analyses, the tissues were homogenized in guanidine thiocyanate/2-mercaptoethanol buffer, and total RNA was extracted using the sodium acetate/phenol/chloroform/isoamyl alcohol method. Then, the samples were precipitated with isopropanol and washed twice with 70% ethanol. The resulting pellets were dissolved in RNase-free Tris-HCl buffer (pH 7.0), and the RNA concentrations were measured using a spectrophotometer (BioPhotometer; Eppendorf). Reverse transcription was performed with 1 µg of total RNA using M-MLV reverse transcriptase (Promega). Quantitative real-time PCR was performed on a 7300 Real-Time PCR System with Sequence Detection Software qPCR v1.3.1 (Applied Biosystems) utilizing Fast SYBR^TM^ Green Master Mix (4385612, Applied Biosystems). All the qRT‒PCR experiments were performed in a blinded manner, as the coded cDNA samples were pipetted by a technician. The target gene mRNA levels were normalized to the levels of apolipoprotein E (*ApoE*) and glyceraldehyde-3-phosphate dehydrogenase (*GAPDH*). The sequences of the primers were described in previous studies^31,98^: *ApoE*: 5‘-CCTGAACCGCTTCTGGGATT-3‘, 5‘-GCTCTTCCTGGACCTGGTCA-3‘; *GAPDH*: 5‘-TGTCCGTCGTGGATCTGAC-3‘, 5‘-CCTGCTTCACCACCTTCTTG-3‘; Homer1a: 5‘-CAAACACTGTTTATGGACTG-3‘, 5‘-TGCTGAATTGAATGTGTACC-3‘; Arc: 5‘-TGGCCCCCAGCAGTGATTCATAC−3‘, 5‘-TGCCCTTTCCAGACATGGCAGC−3‘; c-fos: 5‘-ATCCTTGGAGCCAGTCAAGA−3‘, 5‘-ATGATGCCGGAAACAAGAAG−3‘; *Npas4:* 5‘-CAGGGACAGGTTAGGGTTCA−3‘, 5‘-TTCAGCAGATCAGCCAGTTG−3‘.

### Patch-clamp electrophysiology

The mice were preoxygenated in a 100% oxygen atmosphere for 5 min before cervical dislocation and then decapitated according to national and institutional guidelines. Three hundred-micrometer-thick transverse slices from the HIPP and coronal slices containing the prefrontal cortex (PFC) were cut with a vibratome (VT1200, Leica Biosystems, Germany). The slices were prepared in artificial cerebrospinal fluid (aCSF) containing (in mM): 125 NaCl, 25 NaHCO_3_, 1.25 NaH_2_PO_4_, 2.5 KCl, 1 MgCl_2_, 27 glucose, and 2 CaCl_2_ (bubbled with carbogen (95% O_2_, 5% CO_2_)).

After incubation for 20 min at 35 °C, the slices were maintained at room temperature in aCSF, transferred to the recording chamber (volume of approx. 2-3 ml) and continuously superfused with aCSF (rate of approx. 5–10 ml/min-1). Differential interference contrast video microscopy was used to assess the location and morphology of CA1 pyramidal neurons and layer II/III pyramidal neurons of the frontal cortex (Zeiss Axioskop 2 FS plus, Zeiss Microscopy, Germany). In addition to optical identification, neurons are classified according to the adaptation of their characteristic firing frequency to long depolarizing current pulses. Borosilicate glass tubes (2.0 mm outer diameter, 0.5 mm wall thickness; Hilgenberg, Germany) were used to pull the patch pipettes. The pipettes had an open resistance of 5–10 MΩ, and the series resistance (Rs) of 10–50 MΩ was compensated by a bridge balance. The patch pipettes were filled with an internal solution that contained (in mM) 132 K-gluconate, 20 KCl, 2 MgCl_2_, 10 HEPES, 0.1 EGTA, 4 Na_2_ATP, and 0.3 NaGTP (pH adjusted to 7.2 with KOH). For filling cells with Biocytin, the substance was dissolved in the internal solution by vortexing (10 mg/2 ml; Sigma, #B4261). Osmolarity (300-310 mosmol/L) was controlled at the beginning of each experimental day with an osmometer (Osmomat, Gonotec, Germany).

A stimulation pipette (a patch pipette with an open resistance of 1–3 MΩ when filled with internal solution) was placed superficially in the stratum radiatum of the CA1 region approximately 30–50 µm from the PC layer. For PFC recordings, the stimulation pipette was placed approximately 100 µm from the patched neuron to stimulate nearby collateral fibers. Subthreshold EPSPs of 2–7 mV were evoked by Schaffer collateral stimulation or collateral fiber stimulation in the PFC with voltage pulses of 10–80 V (frequency of 0.1 Hz, duration of 200 µs) using a stimulus isolator (Model 2100 isolated pulse stimulator, A-M Systems, USA). The resting membrane potentials were between −75 and −65 mV, and the holding potential was −70 mV. Hyperpolarizing voltage test pulses (50 ms/−5 mV) were applied to assess the input (Rm) and series (Rs) resistance after every 10th EPSP. EPSPs were combined with postsynaptic action potentials (APs) triggered by short (3 ms) 700 pA current injections via the patch-clamp electrode. Five EPSPs and five postsynaptic APs were paired at 100 Hz with a 5 ms delay (AP after EPSP). Five of these bursts of synchronized EPSP → AP pairs were applied at the theta frequency (5 Hz), followed by an interval of 10 s and 4 more theta blocks, resulting in 125 EPSP/AP pairings.

To assess intrinsic excitability of PFC neurons, a ramp current injection protocol was applied in current-clamp mode, in which the injected current was linearly increased from 0 to 600 pA over 100 ms. The latency to the first action potential was measured as the primary readout of intrinsic excitability. This protocol was performed at baseline and following wash-in of the DREADD agonist Compound 21 (C21; 100 nM) in two experimental groups: (i) control mCherry neurons, and (ii) neurons from animals expressing the excitatory hM3Dq DREADD.

To assess EPSP-to-action potential conversion in PFC neurons, stimulation intensity was set to 95 V. Only neurons exhibiting subthreshold EPSPs without action potential generation at this intensity prior to C21 application were included in this analysis. Following C21 wash-in, the proportion of neurons in which the same 95 V stimulus evoked an action potential was determined and compared between groups. Neurons were injected with biocytin during the recording. To preserve cellular morphology, electrodes were carefully withdrawn after the recording. Following recordings, slices were fixed in 4% paraformaldehyde (PFA) in phosphate-buffered saline (PBS) for 24 h at 4 °C under gentle agitation.

EPC-10 amplifiers (HEKA, Germany) were used, and the signals were filtered at 5 kHz. Patchmaster NEXT software (Version 1.2, HEKA, Germany) was used for data acquisition and initial analysis. All the experiments were performed at room temperature.

The experiments were discarded if (i) the Rs changed by more than 30% during the experiment, (ii) evidence of ictal discharges was observed, (iii) the membrane potential between the start and end of the experiment differed by more than 5 mV, or (iv) the neurons did not respond to a firing pattern control pulse at the beginning and end of the experiments. A maximum of two hippocampal slices per animal were used. All the groups included at least 6 animals.

Analysis of electrophysiological recordings: In the LTP figures, each dot represents ten consecutive averaged maximum EPSP amplitudes ± SEM. The mean amplitude of 30 consecutive EPSPs was calculated before the induction protocol to determine the baseline amplitude. Twenty minutes after LTP induction, the mean amplitude of 60 consecutive EPSPs was calculated and compared to the baseline value from the same experiment with a two-tailed *t* test. EPSP amplitudes in PFC recordings were likewise compared before and after C21 wash-in using a two-tailed paired t test, and intrinsic excitability measures were compared between groups using a two-tailed unpaired *t* test. The proportion of neurons converting from subthreshold EPSPs to action potential firing following C21 wash-in was compared between groups using Fisher’s exact test. Changes in EPSP amplitudes are expressed as percentages of the baseline measurements and were analyzed for each experimental group to quantify the degree of LTP.

### Alexa-Streptavidin labeling and mounting of biocytin filled cells

Blocking solution was prepared by dissolving 5 g sucrose and 2 g bovine serum albumin (BSA; Sigma) in 90 mL PBS, followed by the addition of 9 mL 10% Triton X-100. The solution was warmed to dissolve all components, aliquoted in 2 mL portions, and stored at −20 °C. Fixed slices from ex-vivo electrophysiological recordings from the PFC were incubated in a blocking solution containing Alexa Fluor 488-conjugated streptavidin (1:500) for 48 h at 4 °C or over two days, with gentle agitation. Slices were then washed thoroughly in PBS at room temperature and mounted in ProLong Antifade Gold (Invitrogen).

### In vivo Local Field Potential (LFP) recordings

For chronic LFP recordings, five wire electrodes (90% platinum / 10% iridium oxide, Teflon-coated, 125 µm diameter, World Precision Instruments, USA) were implanted bilaterally in the mPFC ( + 1.67 AP, ±0.3 ML, −2.8 DV relative to bregma), in the ventral hippocampus ( − 3.35 AP, ±3.65 ML, −4.0 DV), and a single electrode in the nucleus reuniens ( − 0.85 AP, 1.17 ML with 15° angle, −4.55 DV). Two stainless-steel screws above the cerebellum served as ground and reference electrode. The implant was secured with dental cement. After surgery, mice were allowed to recover for three weeks before the start of recordings. Electrode placement was verified post hoc by histological examination; electrodes located outside the target region were excluded from analysis, and inter-regional connectivity metrics were computed only from ipsilateral electrode pairs.

LFP signals were acquired using an Open Ephys Acquisition Board (Open Ephys, USA) connected to a 32-channel headstage (SPI Headstage 32ch, Intan) via a custom 8-pin-to-Omnetics adapter. Recordings were synchronized to video acquisition (Basler acA 1300-60 gm camera) via TTL pulses. Each recording session consisted of a 10-min habituation period in an open field (40 × 30 ×35 cm), immediately followed by a 10-min novel object exploration (NOE) period during which a single previously unseen object was introduced into the arena. The paradigm was conducted on two consecutive days at baseline (naive), repeated after CDM induction (with two intervening rest days), and performed once more 10 min after acute C21 administration (0.75 mg/kg, i.p.). A different novel object was used for each session. The position of the animal’s nose was tracked using DeepLabCut with a custom-trained model. For habituation analysis, running epochs were defined as periods with speed ≥ 5 cm/s and minimum duration of 0.5 s. For the NOE analysis, exploration bouts were identified as periods of at least 0.5 s during which the nose was located within a region of interest spreading 25px around the object.

LFP signals were downsampled to 1000 Hz using a polyphase anti-aliasing filter and mean-detrended. The following frequency bands were defined: delta (1–4 Hz), theta (4–12 Hz), beta (13–30 Hz), low gamma (30–60 Hz), and high gamma (60–100 Hz). LFP data were segmented into 0.5-s windows with 50% overlap. Windows in which the peak amplitude exceeded the median plus 4 times the robust standard deviation were rejected as artifacts. Power spectral density was estimated using Welch’s method (0.5-s segments). Band power was computed as the mean squared amplitude of the bandpass-filtered signal (3rd-order Butterworth) within each window. Coherence was estimated using a multitaper method (time-bandwidth product NW = 2, 3 Slepian tapers), and averaged within each frequency band. Phase-amplitude coupling between theta phase and low-gamma amplitude was quantified using the modulation index (MI) with 18 phase bins^99^. For the NOE analysis, each metric was computed separately for exploration windows (event) and for a speed-matched baseline. Baseline windows were sampled from non-exploration, non-object-zone periods in which the locomotion speed fell within ± 1 SD of the mean exploration speed. The change in each metric (Δ = event − baseline) was used for statistical comparison. For each metric, values from repeated sessions within the same phase were averaged. Values were z-score normalized within each subject across the three conditions.

### Immunohistochemistry

After intracardiac perfusion, the brains of the mice were extracted with 20 mL of ice-cold phosphate-buffered saline (PBS; 8.1 mM Na_2_HPO_4_, 138 mM NaCl, 2.7 mM KCl and 1.47 mM KH_2_PO_4_ [pH 7.4]), followed by 15 mL of 4% paraformaldehyde (PFA) in PBS. The brains were subsequently fixed in 4% PFA/PBS for 6 h at 4 °C and then cryoprotected for 48 hours in a 30% sucrose/PBS solution at 4 °C. After being sectioned with a cryostat, 30-μm-thick sections were washed and blocked with 4% normal goat serum in PBS for 1 h at room temperature. The primary antibodies were diluted in PBS, 0.3% Triton X-100, and 1% goat serum, added to the sections, and incubated overnight at 4 °C with agitation. The sections were then washed three times for five minutes each with PBS supplemented with 1% Triton X-100, followed by incubation for one hour at room temperature with secondary antibodies diluted in PBS supplemented with 1% Triton X-100 and 1% goat serum. The sections were then incubated for 15 min at room temperature with the nuclear stain 4,6-diamidino-2-phenylindole (DAPI; Thermo Fisher Scientific, USA; 1:1000) in PBS supplemented with 0.2% Triton. After washing, the sections were mounted with Mowiol-DABCO (803456, Merck, Germany).

The following primary antibodies were used: guinea pig polyclonal anti-VGLUT1 (AB5905, 1:1000; Millipore, USA, lot #4141952), anti-VGLUT2 (AB2251-l, 1:1000; Millipore, USA, lot # 4137617) and mouse anti-GAD65 (ab26113, 1:500; Abcam, United Kingdom, lot #GR3308495-2). The following secondary antibodies were used: goat polyclonal anti-guinea pig Alexa Fluor 594 (Invitrogen, USA; A-11076, 2 μg/mL; lot #2540867) and goat polyclonal anti-mouse Alexa Fluor 488 (A-32723, 2 μg/mL; Invitrogen, USA; lot #XA336883). All the images were captured using a Zeiss Celldiscoverer 7 microscope equipped with a confocal LSM 900 with AiryScan 2, and acquired with ZEN Blue 3.4. The objective used was a Plan-Apochromat 50 ×/1.2 W, N.A. 1.2, H_2_O immersion. The laser intensity and gain were set to occupy the full dynamic range of the detector.

### Morphological analysis

All presynaptic quantifications were performed by a blinded experimenter. For each animal, one field of view per region was acquired in each of two sections, and values were averaged. Confocal z-stacks were acquired in the following regions: for the hippocampus, VGLUT2 and GAD65 puncta were imaged in the stratum lacunosum-moleculare (SLM), and VGLUT1 puncta in the stratum radiatum (SR), in both dorsal and ventral CA1. For the mPFC, images were acquired in the deep layers of the IL. For the RE, images were acquired in the medial portion of the nucleus. After Airyscan processing, a consistent z-depth was selected per marker to ensure uniform sampling across all images within each antibody channel.

Puncta were segmented and quantified using the supervised machine learning software Ilastik^100^. The pixel classification workflow was applied: positive and negative pixels were manually labeled on a representative subset of images from both experimental groups, all features were selected, and a segmentation classifier was trained and batch-applied to all images for each marker independently (VGLUT1, VGLUT2, and GAD65 were analyzed separately), generating pixel prediction maps. These maps were then processed using the object classification workflow: raw images and corresponding pixel prediction maps were loaded, hysteresis thresholding was applied to segment individual puncta, all features except location were selected, and an object classifier was trained and batch-applied. This yielded for each punctum its identity and volume, from which puncta density and mean puncta volume were calculated.

### Fiber photometry: data acquisition and analysis

The fiber photometry setup consisted of a Doric fiber photometry console (FPC, Doric Lenses, Canada), a 6-port fluorescent minicube with an integrated LED and detector (ilFMC6-G2, Doric Lenses, Canada), and a USB DAQ board (USB-6210, National Instruments, USA). The minicube was connected to the implanted ferrule via a low-autofluorescence patch cord (400 µm core diameter, 0.37 NA, MFP_400/440/1100-0.37_5m_FCM-MF2.5_LAF, Doric Lenses, Canada) and interconnection sleeve (SLEEVE_BR_2.5-BK, Doric, Canada). Excitation light was delivered from LEDs at 470 nm (for the GCaMP signal) or 520 nm (for RGECO1a). An isosbestic channel (405 nm LED) was used for both the green and red sensors to control for motion artifacts and photobleaching. The emission light was collected through the same optical fiber, passed through a GFP emission filter (525/50 nm) for the GCaMP signals or an RFP emission filter (630/60 nm) for the RGECO1a signals, and reached the built-in photodetector set up in lock-in detection mode. Videos of the mice were simultaneously recorded with the IR-illuminated camera at a 30 Hz frame rate (DMK 33GX290, Imaging Source). The data were collected by the DAQ at a 2-kHz sampling rate using a custom LabVIEW script (National Instruments, USA).

Fiber photometry data were first demodulated using a custom MATLAB script, resulting in an effective 20-Hz rate for fluorescent recordings. The signal analysis pipeline was then implemented following a custom adaptation of the GuPPy python toolbox^101^. Briefly, the signal was first filtered using a zero-phase moving average linear digital filter, an isosbestic control was fitted to the signal (least square linear regression), and ∆F/F was calculated by subtracting and dividing the control data from the signal data. Mouse behavior was recorded during a session and automatically analyzed using DeepLabCut^38^ for pose estimation. Episodes of immobility and struggle were filtered using two temporal criteria: each bout was required to last a minimum duration of 3 s and had to be preceded by at least 3 s of the opposite behavior. The resulting timestamps were analyzed together with the ∆F/F signal using a custom Python script. To calculate the global mean peak amplitude, individual Ca²⁺ transients were detected across the full cumulative periods of struggle or immobility using the find_peaks function from the scipy library. The amplitudes of all detected transients within each behavioral state were then averaged to yield a single mean peak amplitude value per animal per condition.

### Histological verification of viral expression and fiber photometry implant localization

To verify the spatial distribution of viral expression across all experimental groups, widefield fluorescence images of slices containing the injection site were acquired and stained with DAPI. The images were registered to the Allen Brain Atlas using BigWarp^102^, a landmark-based deformable registration plugin for ImageJ/Fiji. Briefly, anatomical landmarks were placed on the DAPI-stained section and their corresponding positions on the appropriate Allen Brain Atlas coronal reference plate. The resulting transformation was applied to generate a warped atlas overlay on the tissue section. Regions of interest (ROIs) were then manually delineated on the registered image according to atlas boundaries. For mPFC injection sites, the following regions were delineated: infralimbic cortex (IL), prelimbic cortex (PrL), anterior cingulate cortex (ACC), dorsal peduncular cortex (DP), and taenia tecta dorsal (TTd). For thalamic injection sites, the following regions were delineated: nucleus reuniens (RE), rhomboid nucleus (RH), central medial nucleus (CM), submedial nucleus (SMT), ventromedial nucleus (VM), and hypothalamus (HY).

The mCherry fluorescence channel was binarized in ImageJ/Fiji. The raw integrated density (RawIntDen) of binarized fluorescence was measured within each ROI. The proportion of viral expression within each region was calculated as the ratio of the RawIntDen in that region to the sum of RawIntDen across all delineated regions (RawIntDen_region / ΣRawIntDen_all regions), expressed as a percentage. Statistical comparisons between the target region and each surrounding region were performed using one-way ANOVA with Dunnett’s post-hoc test. This quantification was performed for all 10 experimental groups spanning the three nodes of the circuit.

For the intersectional strategies (IL → RE and RE → HIPP), the Cre-dependent DIO constructs require Cre-mediated recombination for expression. The distribution of DIO-hM4Di-mCherry or DIO-mCherry fluorescence therefore directly reports the spatial distribution of functionally manipulated neurons, and was quantified using the same pipeline as described above.

For fiber photometry experiments, implant positions were verified by identifying the fiber tract and tip location in DAPI-stained coronal sections. The fiber tip position for each animal was manually mapped onto coronal atlas templates at three anteroposterior levels (Bregma −3.28, −3.38, and −3.64 mm). Animals whose fiber tip was located outside of the CA1 region were excluded from the analyses.

### Statistics and reproducibility

All values are presented as the means, and the error bars represent the SEMs in the figures. For the statistical analyses, GraphPad Prism version 10.0 (GraphPad Software, USA) was used. To test for significance within experimental groups, we used a two-tailed paired Student’s t test or a Wilcoxon signed rank test. To compare two experimental groups, either an unpaired Student’s *t* test or the Mann–Whitney test was performed. Welch’s correction was applied to *t* tests when variances were significantly different. To analyze differences among several treatment groups, one or two-way analysis of variance (ANOVA) followed by Bonferroni’s or Tukey’s post hoc tests was used. The outliers were systematically excluded using ROUT methods set to 1%. All the statistical tests were two-tailed if applicable. Significance levels are indicated by asterisks in the figures: *p < 0.05, **p < 0.01, ***p < 0.001. The exact *P* and F values as well as the tests that were used to analyze each group are listed in Source Data files. Statistical analysis summaries are mentioned in the figure legends. For all the molecular and behavioral studies, the mice were randomly assigned to groups. Most behavioral and molecular results were confirmed via independent replication of the experiments, as shown in Source Data files. Additionally, the investigators were blinded to the treatment groups until the data were collected. Sample sizes were determined on the basis of extensive laboratory experience^16,28,31,32^ and were verified via power analysis. The micrographs show representative images of viral expression (Fig. 1b; 5b, c; 6b; 7b, c), which was verified and quantified across animals in Supplementary Fig. 1a–i. Fiber-optic placements for the fiber photometry experiments (Fig. 3b, e) are mapped in Supplementary Fig. 5a.

### Sex aspects

All experiments were performed with an equal distribution of male and female mice. For all experiments, a separate exploratory analysis focusing on the potential effects of sex on the experiments was performed, but no relevant differences were observed. However, our experiments were not adequately powered to reliably detect sex differences.

### Reporting summary

Further information on research design is available in the Nature Portfolio Reporting Summary linked to this article.

## Supplementary information


Supplementary Information
Peer Review file
Reporting Summary


## Source data


Source Data


## Data Availability

The data generated in this study are provided in the Supplementary Information and the Source Data file. Source data are provided with this paper.

## Code availability

Custom Python scripts used for analyzing fiber photometry and LFP data are available in our GitHub repository (https://github.com/mveleanu/veleanu_et_al) and archived on Zenodo (https://doi.org/10.5281/zenodo.21111701)^103^.
