# Supplementary material for: The mPFC-reuniens-hippocampus pathway links brain circuitry and neural plasticity in antidepressant response
Source: Nat Commun. 2026 Sep 1;17:9361. doi: 10.1038/s41467-026-77248-y (PMC13534506; doi:10.1038/s41467-026-77248-y)
Supplement: Supplementary file 1 [file 41467_2026_77248_MOESM1_ESM.pdf]

Supplementary Information for

The mPFC-Reuniens-Hippocampus Pathway Links Brain Circuitry and Neural Plasticity in Antidepressant Response

Veleanu et al.

Includes:

Supplementary Figures 1 – 9

Supplementary Figure 1

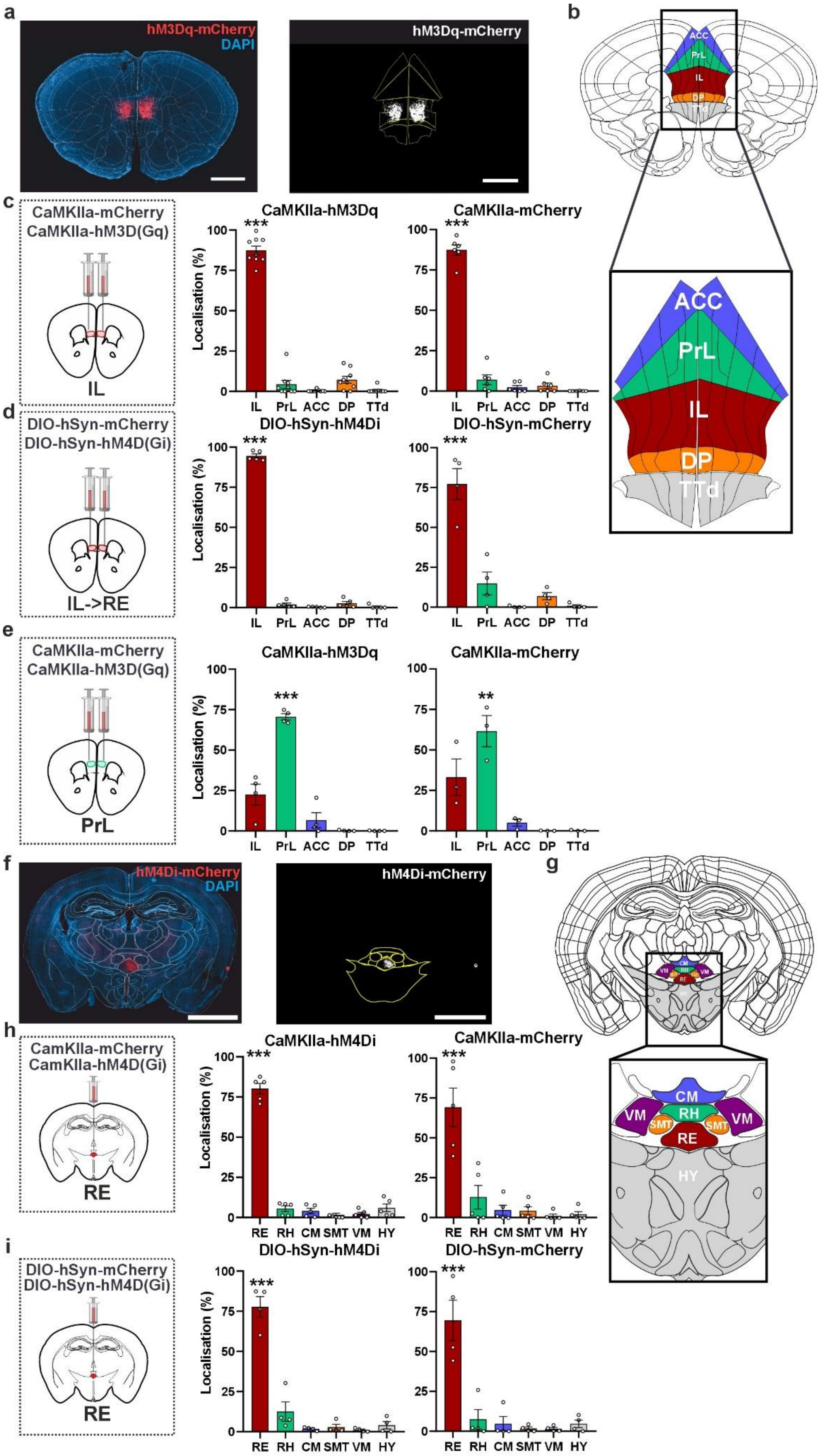

Supplementary Fig. 1 | a Representative confocal image of hM3Dq-mCherry expression in the IL (left) and corresponding binarized fluorescence map registered to the Allen Brain Atlas (right). Scale bar 1000µm. **b** Schematic atlas of the medial prefrontal cortex showing delineation of IL (infralimbic) and the surrounding regions PrL (prelimbic), ACC (anterior cingulate), DP (dorsal peduncular) and TTd (dorsal taenia tecta) used for quantification. **c** Quantification of viral expression distribution for hM3Dq-IL (left, n=9, P<0.0001) and mCherry-IL (right, n=6, P<0.0001) groups across IL compared to surrounding regions. **d** Quantification of viral expression distribution for IL→RE intersectional strategy: DIO-hM4Di (left, n=5, P<0.0001) and DIO-mCherry (right, n=4, P<0.0001) groups. **e** Quantification of viral expression distribution for hM3Dq-PrL (left, n=4, P<0.0001) and mCherry-PrL (right, n=3, P=0.0014) groups across PrL compared to surrounding regions. **f** Representative confocal image of hM4Di expression in the RE (left) and corresponding binarized fluorescence map registered to the Allen Brain Atlas (right). Scale bar 1000µm. **g** Schematic atlas of the thalamic region showing delineation of RE (nucleus reuniens), RH (rhomboid), CM (central medial), SMT (submedial nucleus of the thalamus), VM (ventromedial nucleus) and HY (hypothalamus) subregions used for quantification. **h** Quantification of viral expression distribution for RE inhibition experiment: hM4Di (left, n=5, P<0.0001) and mCherry (right, n=5, P<0.0001) groups. **i** Quantification of viral expression distribution for RE→HIPP intersectional strategy: DIO-hM4Di (left, n=4, P<0.0001) and DIO-mCherry (right, n=4, P<0.0001) groups. Statistical comparisons were performed using RM-one-way ANOVA with Dunnett's post-hoc test. Data are presented as mean ±SEM and the individual data points are depicted. *P<0.05, ***P<0.001. Source data are provided as a Source Data file. Created in BioRender. Vestring, S. (2026) https://BioRender.com/rpcq0ko.

Supplementary Figure 2

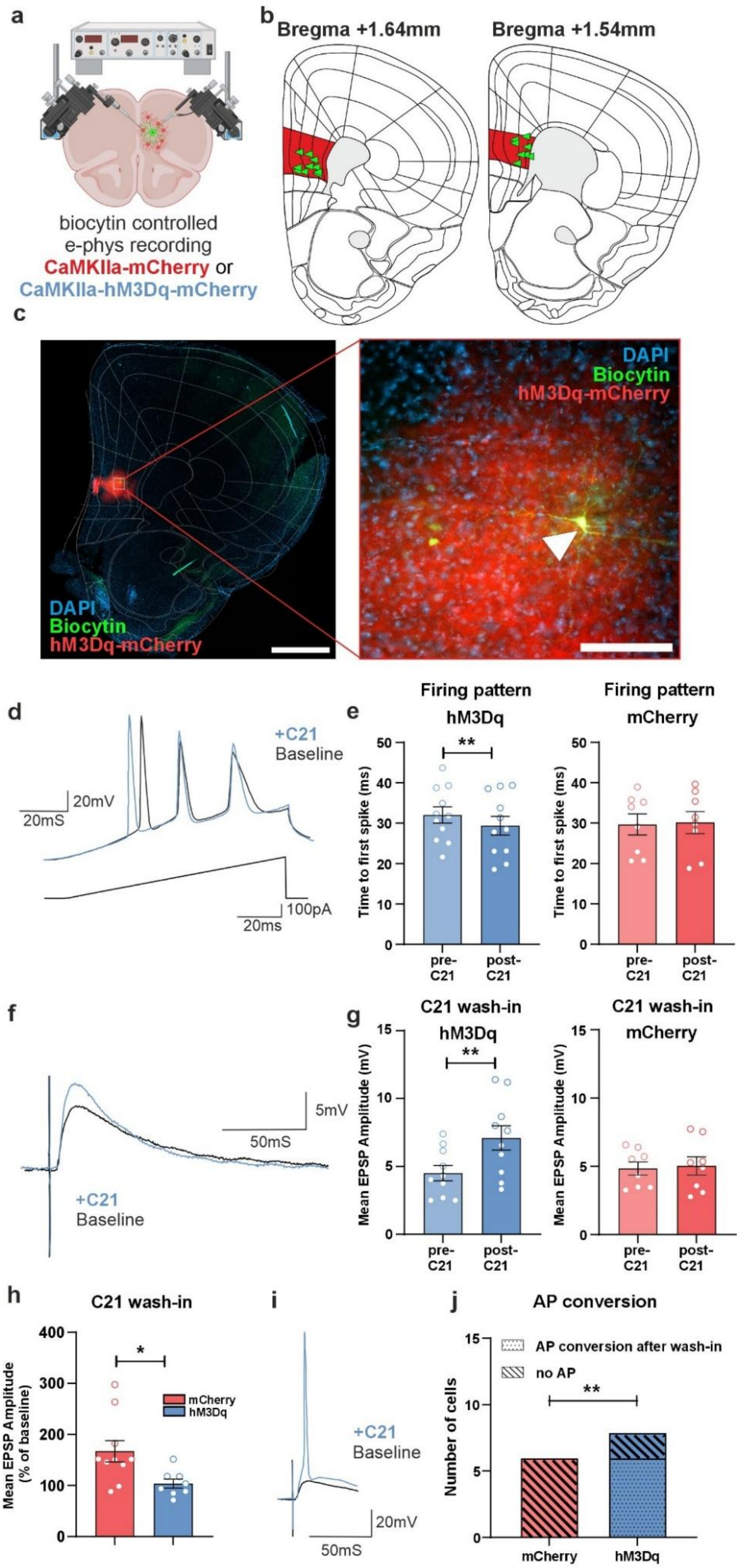

Supplementary Fig. 2 | a Schematic of the experimental setup: whole-cell patch-clamp recordings were performed from biocytin-filled, CaMKIIa-mCherry or CaMKIIa-hM3Dq-mCherry positive pyramidal neurons in acute brain slices containing the IL cortex. **b** Localization of patched neurons within the IL, registered to a standard coronal reference atlas (Paxinos and Franklin). **c** Microscopy images of a recorded neuron showing DAPI (blue), hM3Dq-mCherry (red), and biocytin (green) labelling, representative of n=11 cells. Left: low magnification overview; scale bar: 1000 µm. Right: high magnification showing co-localization; scale bar: 100 µm. White arrowhead indicates the dual-labelled neuron. **d** Upper: representative current-clamp traces showing action potential firing in response to a ramp current injection protocol at baseline (black) and after C21 bath application (blue) in an hM3Dq-expressing neuron. Lower: corresponding ramp current injection protocol. **e** Latency to first action potential evoked by ramp current injection at baseline (pre-C21) and after C21 wash-in. Left: hM3Dq-expressing neurons (n=11 cells, P=0.0094, two-tailed paired t-test). Right: mCherry control neurons (n=8 cells, P=0.6710, two-tailed paired t-test). **f** Representative EPSP traces at baseline (black) and after C21 wash-in (blue). **g** Mean EPSP amplitude at baseline and after C21 wash-in. Left: hM3Dq-expressing neurons (n=10 cells, P=0.0062, two-tailed paired t-test). Right: mCherry control neurons (n=8 cells, P=0.6308, two-tailed paired t-test). **h** Between-group comparison of mean EPSP amplitude change after C21 wash-in, expressed as percentage of baseline (mCherry n=8 cells, hM3Dq n=10 cells, P=0.0210, two-tailed unpaired t-test). **i** Representative traces illustrating subthreshold-to-suprathreshold conversion following C21 wash-in in an hM3Dq-expressing neuron (stimulation intensity: 95 V). Black: baseline; blue: after C21. **j** Contingency analysis of action potential conversion after C21 wash-in. Only neurons exhibiting subthreshold EPSPs at 95 V stimulation prior to C21 application were included. The proportion of neurons converting to suprathreshold firing following C21 wash-in was compared between groups (mCherry: 0/6; hM3Dq: 6/8; P=0.0097, Fisher's exact test). Data are presented as mean ±SEM and the individual data points are depicted. *P<0.05, **P<0.01, ***P<0.001. Source data are provided as a Source Data file. Created in BioRender. Vestring, S. (2026) https://BioRender.com/rpcq0ko.

Supplementary Figure 3

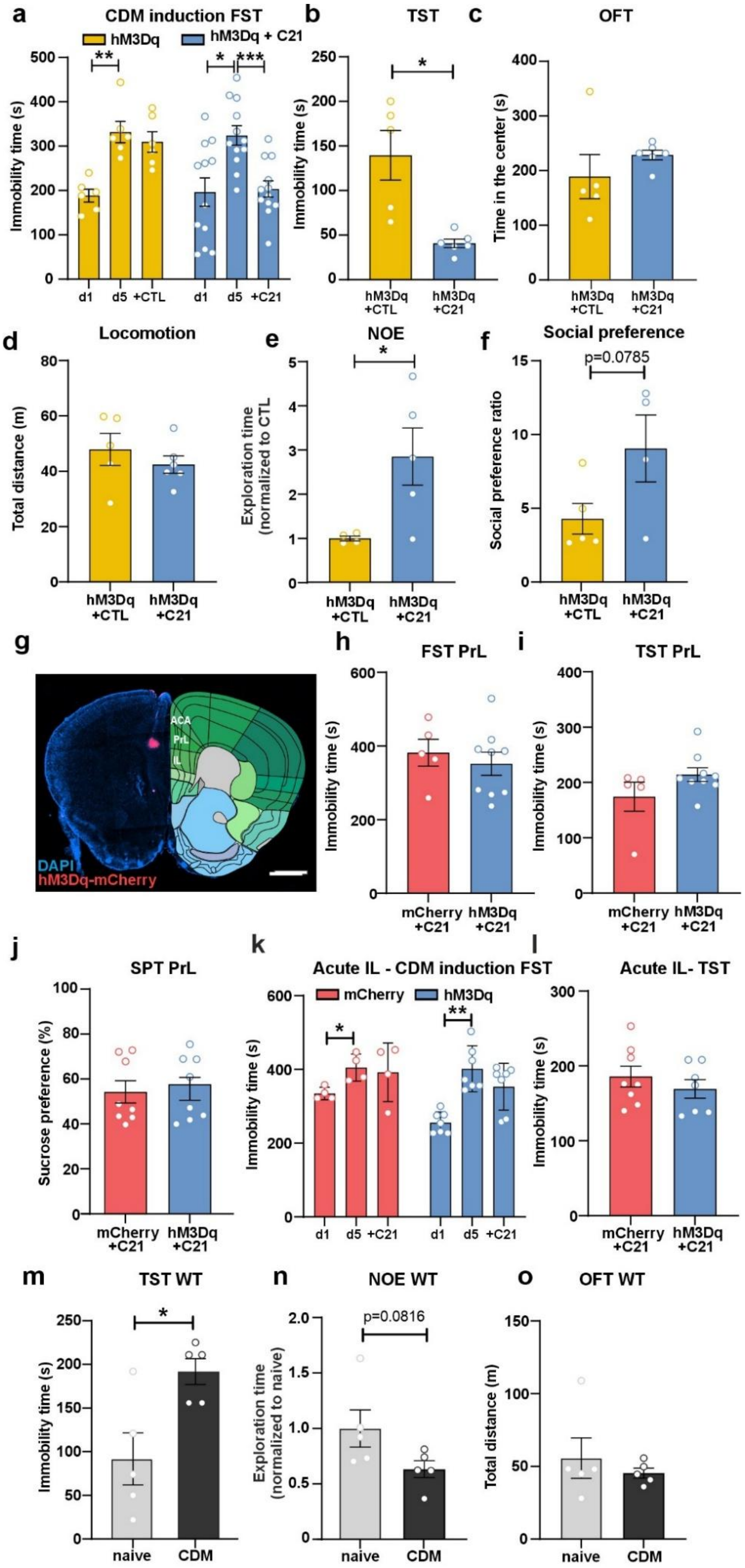

Supplementary Fig. 3 | a Immobility time in CDM of hM3Dq-injected mice receiving either overnight C21 in drinking water or regular drinking water as control. Statistical comparisons: day 1 vs day 5 in control (n=6, *P*=0.0034) and C21-treated groups (n=12, *P*=0.0244); day 5 vs post-treatment in C21-treated (*P*=0.0002) and control groups (*P*>0.9999), repeated measures two-way ANOVA with Bonferroni post-hoc test. **b** Immobility time in TST between hM3Dq-injected mice overnight treated with C21 (n=6) and controls (n=5), *P*=0.0225, Welch's t-test. **c** Time spent in center of Open Field Test (OFT) between hM3Dq-injected mice treated with C21 (n=6) and untreated controls (n=5), *P*=0.3862, Welch's t-test. **d** Locomotion measured by distance travelled in OFT between hM3Dq-injected mice treated with C21 (n=6) and untreated controls (n=5), *P*=0.4074, unpaired Student's t-test. **e** Total time spent exploring two novel objects between hM3Dq-injected mice treated with C21 (n=5) and untreated controls (n=4), *P*=0.0454, Welch's t-test. **f** Social interaction measured in three-chamber task between hM3Dq-injected mice treated with C21 (n=5) and untreated controls (n=4), *P*=0.0785, unpaired Student's t-test. **g** Representative image of AAV-CaMKIIα-hM3D(Gq)-mCherry injection in lateral prelimbic cortex (PrL), co-registered with the Allen Brain Atlas. Scale bar: 1000 µm. **h** Immobility time in FST between mice PrL-injected with hM3Dq receiving C21 treatment (n=9) and mCherry with C21 treatment (n=5), *P*=0.5641, unpaired Student's t test. **i** Immobility time in TST between mice PrL-injected with hM3Dq receiving C21 treatment (n=9) and mCherry with C21 treatment (n=5), *P*=0.1410, unpaired Student's t test. **j** Sucrose preference between C21-treated hM3Dq and mCherry mice (n=8, *P*=0.8785, Mann-Whitney test). **k** Immobility time in CDM paradigm after acute C21 treatment (0.75 mg/kg, i.p., 2h before testing) of mCherry (n=4 mice, day 1 vs day 5 *P*=0.0363; day 5 vs C21 *P*=0.9649) and hM3Dq (n=7 mice, day 1 vs day 5 P=0.0014; day 5 vs C21 *P*=0.2921), repeated measures two-way ANOVA with Bonferroni post-hoc test. **l** Immobility time in TST after acute C21 treatment (mCherry + C21: n=8; hM3Dq + C21: n=7; *P*=0.3930, unpaired Student's t-test). **m** Immobility time in TST comparing wild-type CDM and naïve mice (n=5, *P*=0.0171, unpaired Student's t-test). **n** Total time spent exploring two novel objects in NOE between CDM and naïve WT mice. Data normalized to naïve group (n=5, *P*=0.0816, unpaired Student's t-test). **o** Locomotion measured by distance travelled in OFT between CDM and naïve mice (n=5, *P*=0.5057, Welch's t-test). Data are presented as mean ±SEM and the individual data points are depicted. **P*<0.05, ***P*<0.01, ****P*<0.001. Source data are provided as a Source Data file.

Supplementary Figure 4

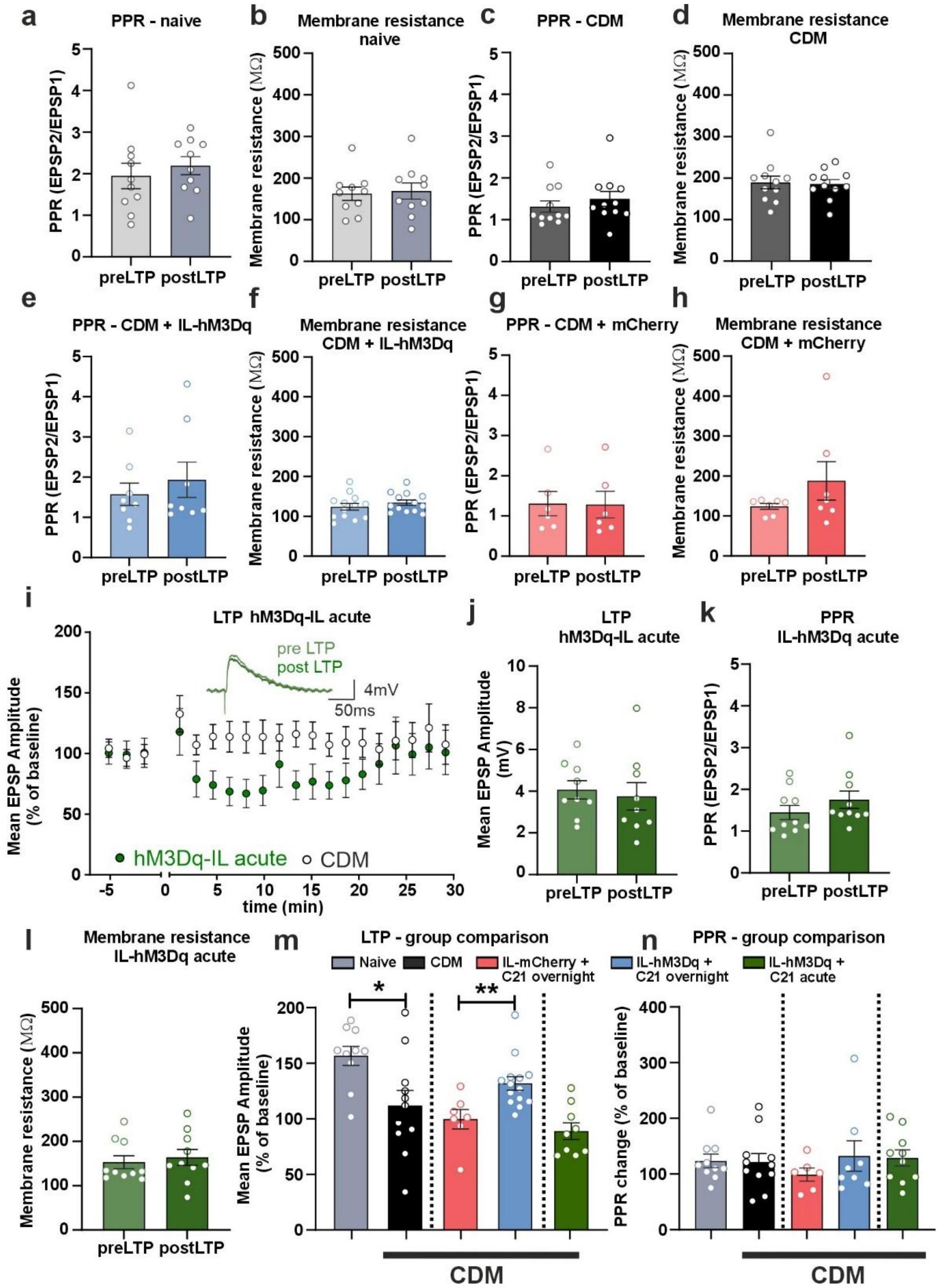

Supplementary Fig. 4 | a PPR pre- vs. post-aLTP induction in naïve mice (n=10 cells, *P*=0.1864, paired t-test). **b** Input resistance (Rm) before and after aLTP induction in naïve mice (n=10 cells, *P*=0.5068, paired t-test). **c** PPR pre- vs. post-aLTP induction in CDM mice (n=11 cells, *P*=0.2783, Wilcoxon matched-pairs signed rank test). **d** Input resistance (Rm) before and after aLTP induction in CDM mice (n=11 cells, *P*=0.8024, paired t-test). **e** Analysis of PPR pre- vs. post-aLTP induction in hM3Dq mice treated with C21 overnight (n=8 cells, *P*=0.6406, Wilcoxon matched-pairs signed rank test). **f** Input resistance (Rm) before and after aLTP induction in hM3Dq mice treated with C21 overnight (n=13 cells, *P*=0.0743, paired Student's t-test). **g** PPR pre- vs. post-aLTP induction in mCherry mice treated with C21 overnight (n=6 cells, *P*=0.8515, paired t-test). **h** Input resistance (Rm) before and after aLTP induction in

mCherry mice (n=7 cells, P= 0.2188, Wilcoxon matched-pairs signed rank test). **i** Time course of aLTP inducibility after acute C21 treatment (0.75 mg/kg i.p., 2h) in hM3Dq-IL mice (green) compared to CDM (white). **j** Analysis of aLTP after acute C21 treatment pre and post LTP induction (n=9 cells, *P*=0.3775, paired t-test). LTP is not changed by acute C21 treatment. **k** PPR pre- vs. post-aLTP induction in acute C21-treated hM3Dq mice (n=10 cells, *P*=0.1934, Wilcoxon matched-pairs signed rank test). **l** Input resistance (Rm) before and after aLTP induction in acute C21-treated hM3Dq mice (n=10 cells, *P*=0.4316, Wilcoxon matched-pairs signed rank test). **m** Overview of mean EPSP amplitude (% of baseline) after aLTP induction across conditions: naïve, CDM, IL-mCherry + C21 overnight, IL-hM3Dq + C21 overnight, and IL-hM3Dq + C21 acute. Statistical comparisons within each experimental design are reported in Fig. 2d, f. **n** Overview of mean change of PPR (% of baseline) after aLTP induction across conditions: naïve, CDM, IL-mCherry + C21 overnight, IL-hM3Dq + C21 overnight, and IL-hM3Dq + C21 acute. Statistical comparisons are reported in Supplementary Fig. 4a,c,e,g,k. Data are presented as mean ±SEM and the individual data points are depicted. **P*<0.05, ***P*<0.01, ****P*<0.001. Source data are provided as a Source Data file.

Supplementary Figure 5

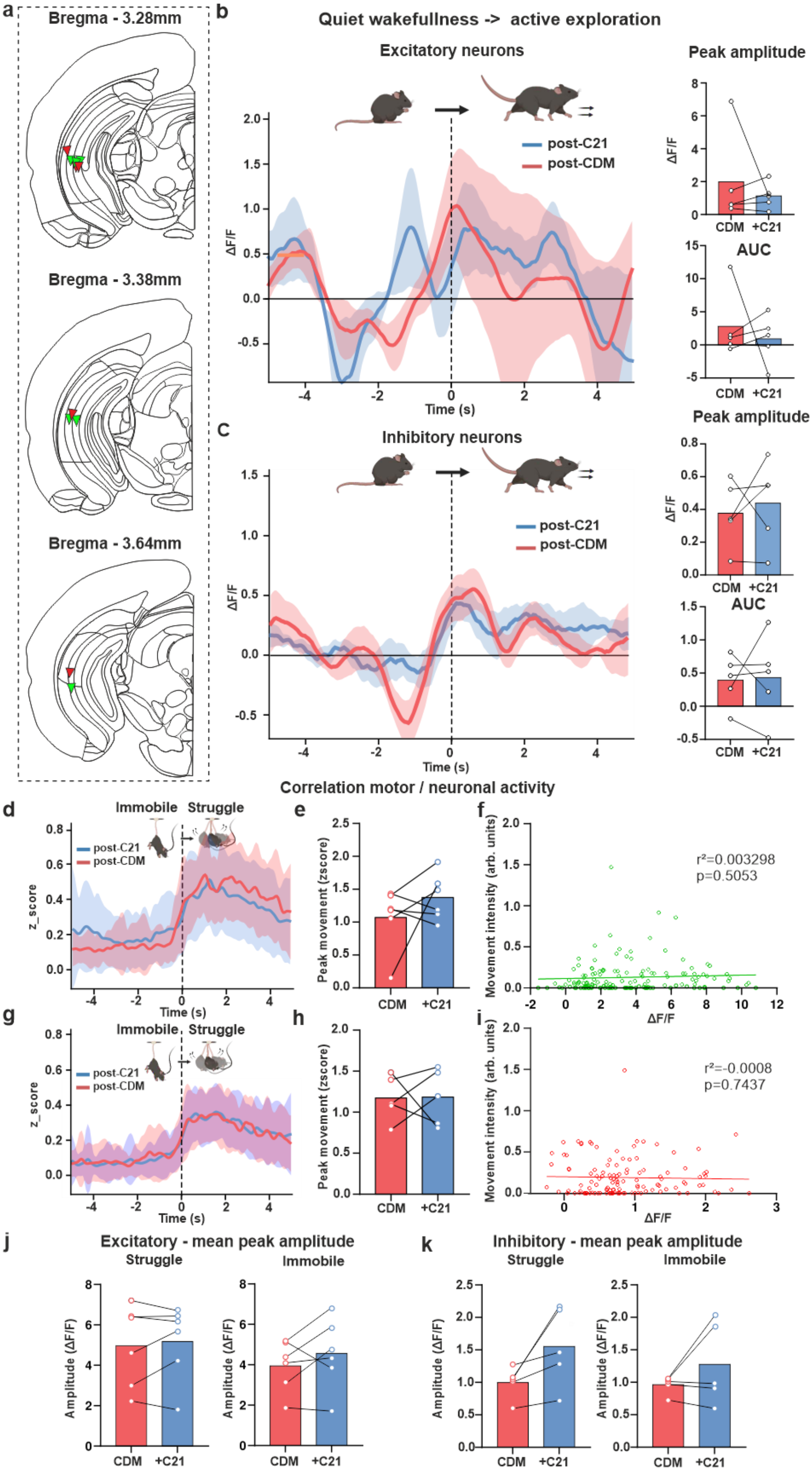

Supplementary Fig. 5 | a Schematic representation of fiber optic cannula placement positions mapped onto coronal atlas sections at three anteroposterior levels (Bregma −3.28, −3.38, −3.64 mm). All fiber tip positions in excitatory (green) and inhibitory (red) fiber photometry experiment were located within ventral CA1. **b** Calcium dynamics of hippocampal excitatory neurons (CaMKIIα-GCaMP8m) during spontaneous quiet wakefulness to active exploration transitions in home cage. Left: Average ΔF/F traces after CDM (post-CDM, red) and following C21 treatment (post-C21, blue). Right: Quantification of peak amplitude (top, n=5, *P*>0.99, Wilcoxon matched-pairs signed rank test) and AUC (bottom, n=5, *P*=0.8125, Wilcoxon matched-pairs signed rank test). **c** Calcium dynamics of hippocampal inhibitory neurons (mDlx-jRGECO1a) during quiet wakefulness to active exploration transitions in home cage. Left: Average ΔF/F traces. Right: Quantification of peak amplitude (top, n=5, *P*=0.6365) and AUC (bottom, n=5, *P*=0.8929). **d** Ethovision PSTH analysis of motor activity (z-scored) aligned to struggle onset during TST for excitatory neuron recordings post-CDM (red) and post-C21 (blue). **e** Peak movement intensity at struggle onset for excitatory recordings (n=6, *P*=0.4375, Wilcoxon matched-pairs signed rank test). **f** Scatter plot of fiber Ca^{2+} peak amplitude vs. Ethovision movement intensity for individual struggle events in GCaMP8m recordings (n=138 events from 6 animals, r^2=0.003298, *P*=0.5035). arb. units: arbitrary units. **g** Ethovision PSTH analysis of motor activity aligned to struggle onset during TST for inhibitory neuron recordings. **h** Peak movement intensity at struggle onset for inhibitory recordings (n=5, *P*=0.955). **i** Scatter plot of fiber Ca^{2+} peak amplitude vs. Ethovision movement intensity for jRGECO1a recordings (n=123 events from 5 animals, r^2=0.0008, *P*=0.7437). **j** Mean peak amplitude of hippocampal excitatory neurons during struggle (left, n=6; *P*=0.5480) and immobile (right, n=6, *P*=0.2766) periods between post-CDM (red) and post-C21 (blue) conditions. arb. units: arbitrary units. **k** Mean peak amplitude of hippocampal inhibitory neurons during struggle (left, n=5, *P*=0.0631) and immobile (right, n=5, *P*=0.26) periods between post-CDM (red) and post-C21 (blue) conditions. Statistical comparisons were performed using two-tailed paired Student's t-test unless otherwise stated. Data are presented as mean ±SEM and the individual data points are depicted. Lines show mean ΔF/F and shaded areas indicate SEM. **P*<0.05, ***P*<0.01, ****P*<0.001. Source data are provided as a Source Data file. Created in BioRender. Vestring, S. (2026) https://BioRender.com/rpcq0ko.

Supplementary Figure 6

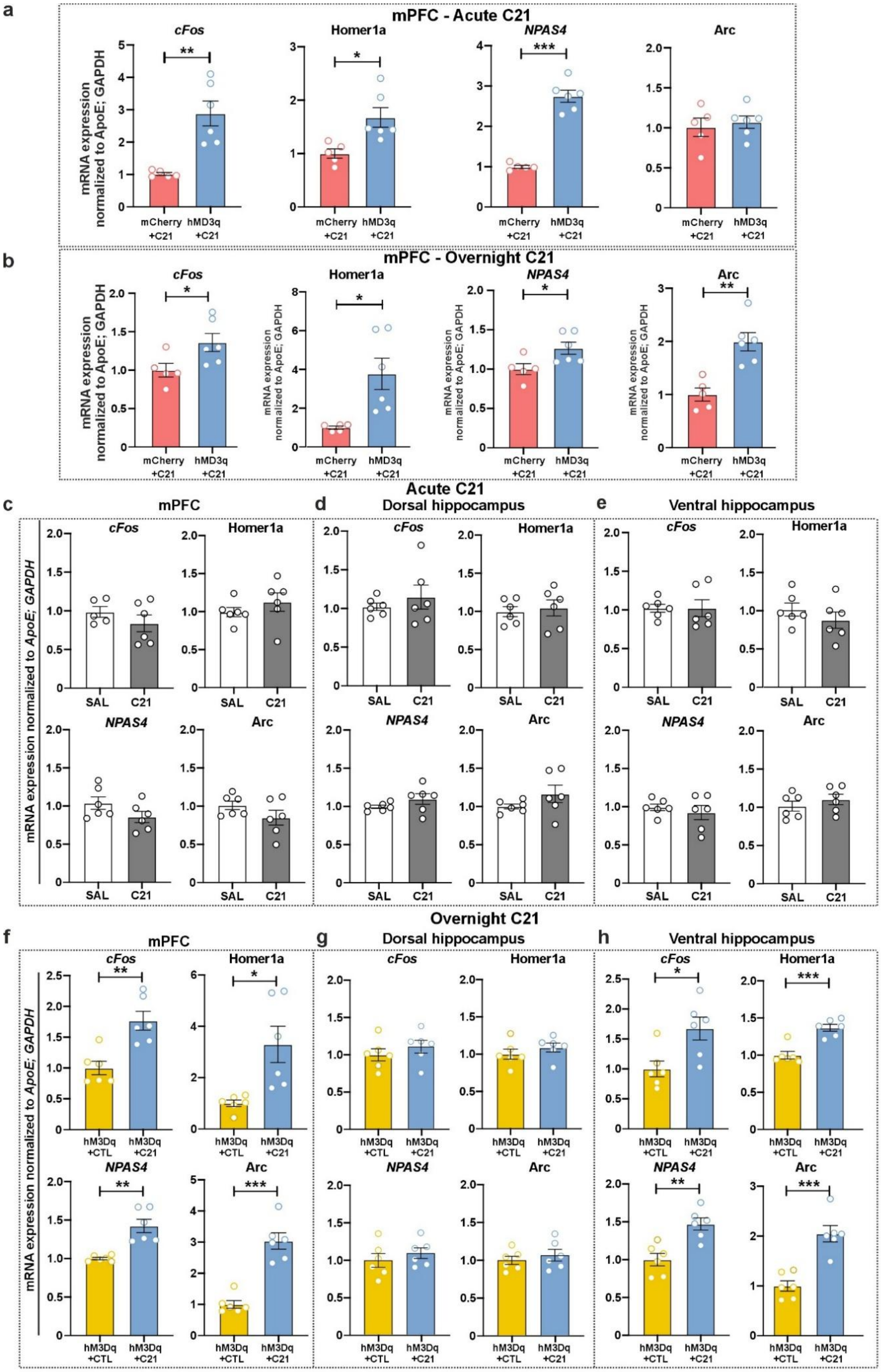

Supplementary Fig. 6 | a mRNA expression of immediate early genes (normalized to ApoE and GAPDH) in acute C21 treatment condition. mPFC comparisons between hM3Dq (n=6) and mCherry (n=5) groups for cFos (*P*=0.0044, Welch's t-test), Homer1a (*P*=0.0127), NPAS4 (*P*<0.0001), and Arc (*P*=0.6465). **b** mRNA expression following overnight C21 treatment: mPFC shows sustained elevation of cFos (*P*=0.0426), Homer1a (*P*=0.0185, Welch's t-test), NPAS4 (*P*=0.0320) and Arc (*P*=0.0015). mRNA expression of immediate early genes (normalized to ApoE and GAPDH). **c-e** mRNA expression following overnight C21 treatment in wild-type mice. **c** mPFC comparisons between C21-treated and saline controls for cFos (saline n=5, C21 n=6, *P*=0.3028), Homer1a (n=6, *P*=0.3514), NPAS4 (n=6, *P*=0.1322), and Arc (n=6, *P*=0.1835). **d** Dorsal hippocampus comparisons between C21-treated and saline controls for cFos (*P*=0.4661, Welch's t-test), Homer1a (*P*=0.7027), NPAS4 (*P*=0.2164, Welch's t-test), and Arc (*P*=0.2179, Welch's t-test); n=6 per group. **e** Ventral hippocampus comparisons between C21-treated and saline controls for cFos (*P*=0.9989), Homer1a (*P*=0.3380), NPAS4 (*P*=0.5117), and Arc (*P*=0.3805); n=6 per group. **f-h** mRNA expression following overnight C21 or regular drinking water as control in hM3Dq injected mice. **f** mPFC comparisons between overnight C21-treated and untreated controls for cFos (*P*=0.0021), Homer1a (*P*=0.0221, Welch's t-test), NPAS4 (*P*=0.0040, Welch's t-test), and Arc (*P*<0.0001). **g** Dorsal hippocampus comparisons between treatments for cFos (*P*=0.1565), Homer1a (*P*=0.3442), NPAS4 (*P*=0.4262), and Arc (*P*=0.4874). **h** Ventral hippocampus comparisons between C21-treated and untreated controls for cFos (*P*=0.0160), Homer1a (*P*=0.0003), NPAS4 (*P*=0.0022), and Arc (*P*=0.0003); n=6 per group. Unless otherwise stated, statistical comparisons were performed using unpaired two-tailed Student's t-test. Data are presented as mean ±SEM and the individual data points are depicted. **P*<0.05, ***P*<0.01, ****P*<0.001. Source data are provided as a Source Data file.

Supplementary Figure 7

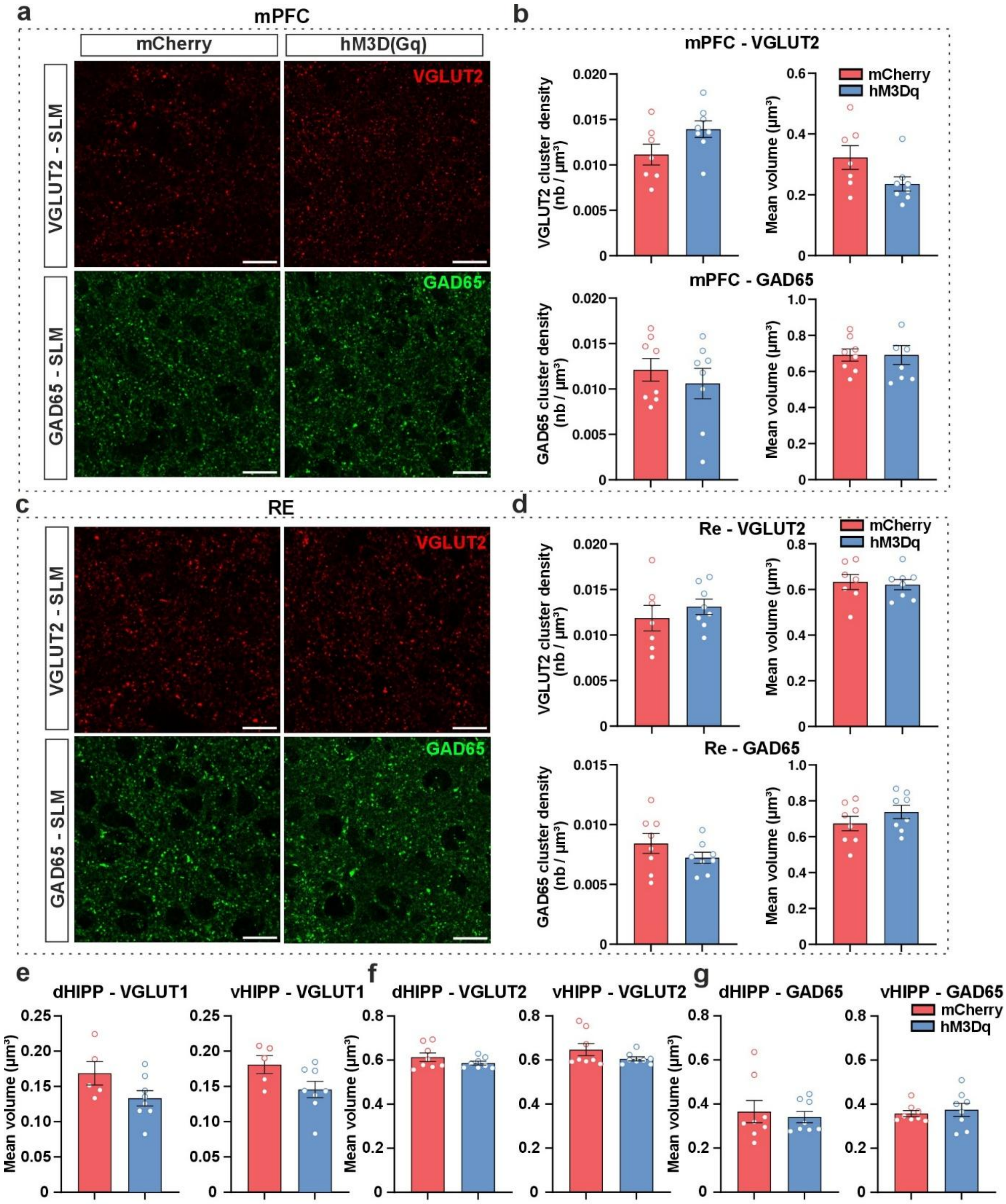

Supplementary Fig. 7 | a Confocal images showing VGLUT2 and GAD65 immunostaining in IL layer V/VI of mCherry and hM3Dq mice after overnight C21 treatment, representative of n=8 mice. Scale bar: 20 µm. **b** Left: quantification of VGLUT2 and GAD65 cluster density in the mPFC comparing hM3Dq and mCherry mice treated with C21 (VGLUT2: mCherry n=7, hM3Dq n=8, *P*=0.0766; GAD65: n=8, *P*=0.4825). Right: quantification of VGLUT2 and GAD65 cluster mean volume (VGLUT2: mCherry n=7, hM3Dq n=8, *P*=0.0712; GAD65: n=8, *P*=0.4987). **c** Representative confocal images showing VGLUT2 and GAD65 immunostaining in the RE of mCherry and hM3Dq mice after overnight C21 treatment. Scale bar: 20 µm. **d** Left: quantification of VGLUT2 and GAD65 cluster density in the RE comparing hM3Dq and

mCherry mice treated with C21 (VGLUT2: mCherry n=7, hM3Dq n=8, *P*=0.4439; GAD65: n=8, *P*=0.2331). Right: quantification of VGLUT2 and GAD65 cluster mean volume (VGLUT2: mCherry n=7, hM3Dq n=8, *P*=0.7737; GAD65: n=8, *P*=0.2553). **e** VGLUT1 presynaptic clusters mean volume comparisons between hM3Dq and mCherry mice treated with C21 in the dorsal (mCherry n=5, hM3Dq n=8, *P*=0.0903) and ventral (mCherry n=5, hM3Dq n=8, *P*=0.0711) hippocampus. **f** VGLUT2 presynaptic clusters mean volume comparisons in the dorsal (left, *P*=0.2312, Welch's t-test) and ventral (right, *P*=0.3953, Mann-Whitney test) hippocampus (n=8). **g** GAD65 presynaptic clusters mean volume comparisons in the dorsal (left, *P*=0.8785, Mann-Whitney test) and ventral (right, *P*=0.6454, Mann-Whitney test) hippocampus (n=8). Unless otherwise stated, statistical comparisons were performed using unpaired two-tailed Student's t-test. Data are presented as mean ±SEM and the individual data points are depicted. **P*<0.05, ***P*<0.01, ****P*<0.001. Source data are provided as a Source Data file.

Supplementary Figure 8

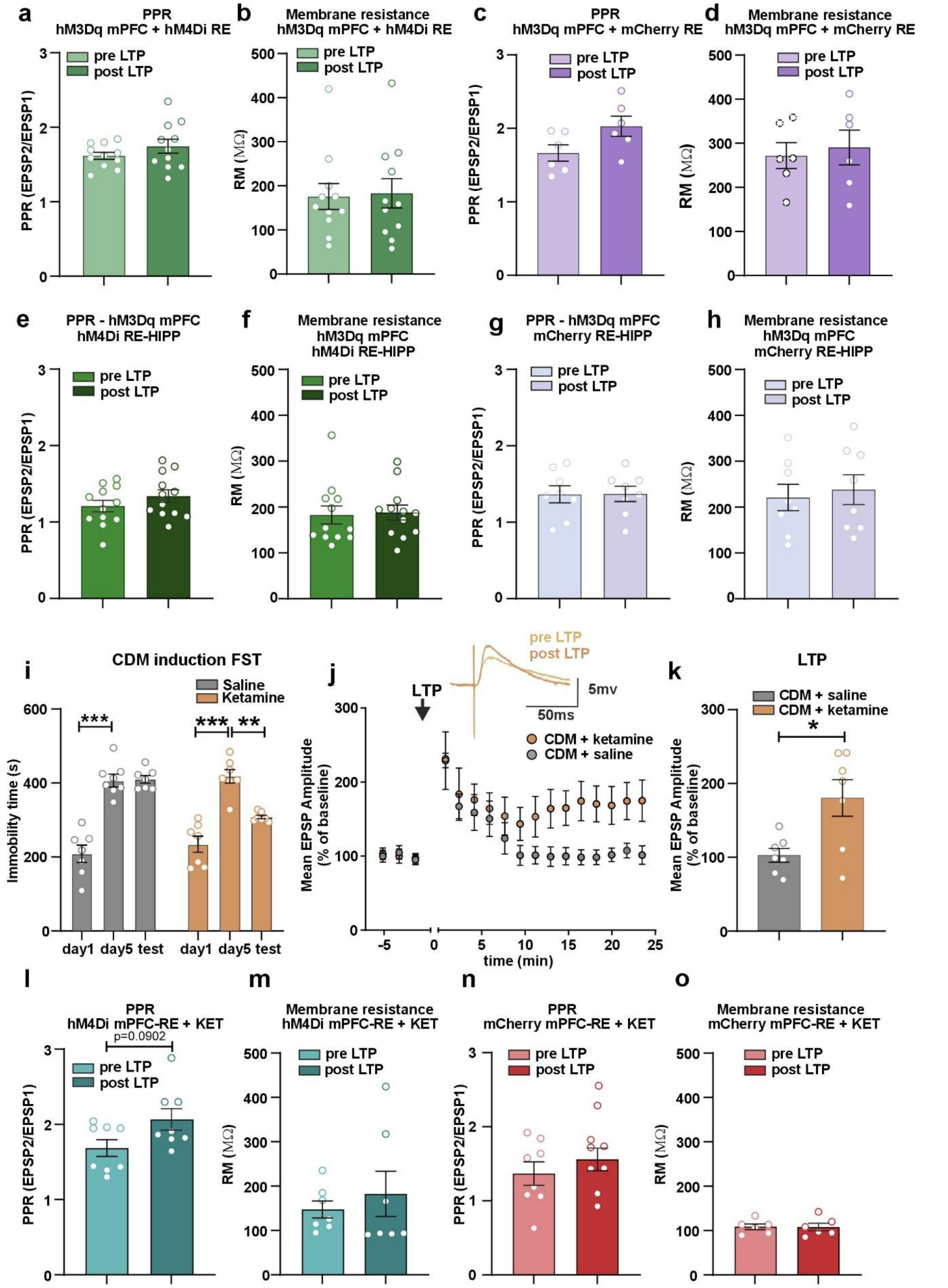

Supplementary Fig. 8 | a PPR comparison pre- vs. post-aLTP induction in hM3Dq mPFC + hM4Di RE mice treated with C21 overnight (n=11 cells, *P*=0.2317, paired Student's t-test). **b**

Input resistance (Rm) before and after aLTP in hM3Dq mPFC + hM4Di RE mice (n=11 cells, *P*=0.8355, paired Student's t-test). **c** PPR comparison pre- vs. post-aLTP in hM3Dq mPFC + mCherry RE mice treated with C21 overnight (n=6 cells, *P*=0.1760, paired Student's t-test). **d** Input resistance (Rm) before and after aLTP in hM3Dq mPFC + mCherry RE mice (n=6 cells, *P*=0.5879, paired Student's t-test). **e** PPR comparison pre- vs. post-aLTP in hM3Dq mPFC + hM4Di RE→HIPP mice (n=12 cells, *P*=0.1874, paired t-test). **f** Input resistance (Rm) before and after aLTP in hM3Dq mPFC + hM4Di RE→HIPP mice (n=12 cells, *P*=0.8501, Wilcoxon matched-pairs signed rank test). **g** PPR comparison pre- vs. post-aLTP in hM3Dq mPFC + mCherry RE→HIPP mice (n=8 cells, *P*=0.9474, paired t-test). **h** Input resistance (Rm) before and after aLTP in hM3Dq mPFC + mCherry RE→HIPP mice (n=8 cells, *P*=0.3227, paired t-test). **i** Swim test immobility time in CDM mice. Statistical comparisons: day 1 vs day 5 in saline (*P*=0.0003) and ketamine (*P*=0.0002) groups; day 5 vs post-treatment in ketamine i.p. (10 mg/kg) (*P*=0.0019) and saline (*P*=0.9873) groups; n=7 per group, repeated measure two-way ANOVA. **j** Time course of aLTP inducibility of ketamine (10 mg/kg) and saline i.p. treated groups. **k** Group analysis (n=7 cells, *P*=0.02, unpaired Student's t-test). **l** Paired-pulse ratio (PPR) comparison before and after LTP induction in Ketamine + hM4Di mPFC-RE (n=8 cells, *P*=0.0902). **m** Input resistance (Rm) comparison before and after LTP induction in Ketamine + hM4Di mPFC-RE (n=7 cells, *P*=0.36). **n** Paired-pulse ratio (PPR) comparison before and after LTP induction in Ketamine + mCherry mPFC-RE (n=8 cells, *P*=0.2831, paired Student's t-test) groups. **o** Input resistance comparison before and after LTP induction in Ketamine + mCherry mPFC-RE (right, n=6 cells, *P*=0.8708), paired Student's t-test. Data are presented as mean ±SEM and the individual data points are depicted. **P*<0.05, ***P*<0.01, ****P*<0.001. Source data are provided as a Source Data file.

Supplementary Figure 9

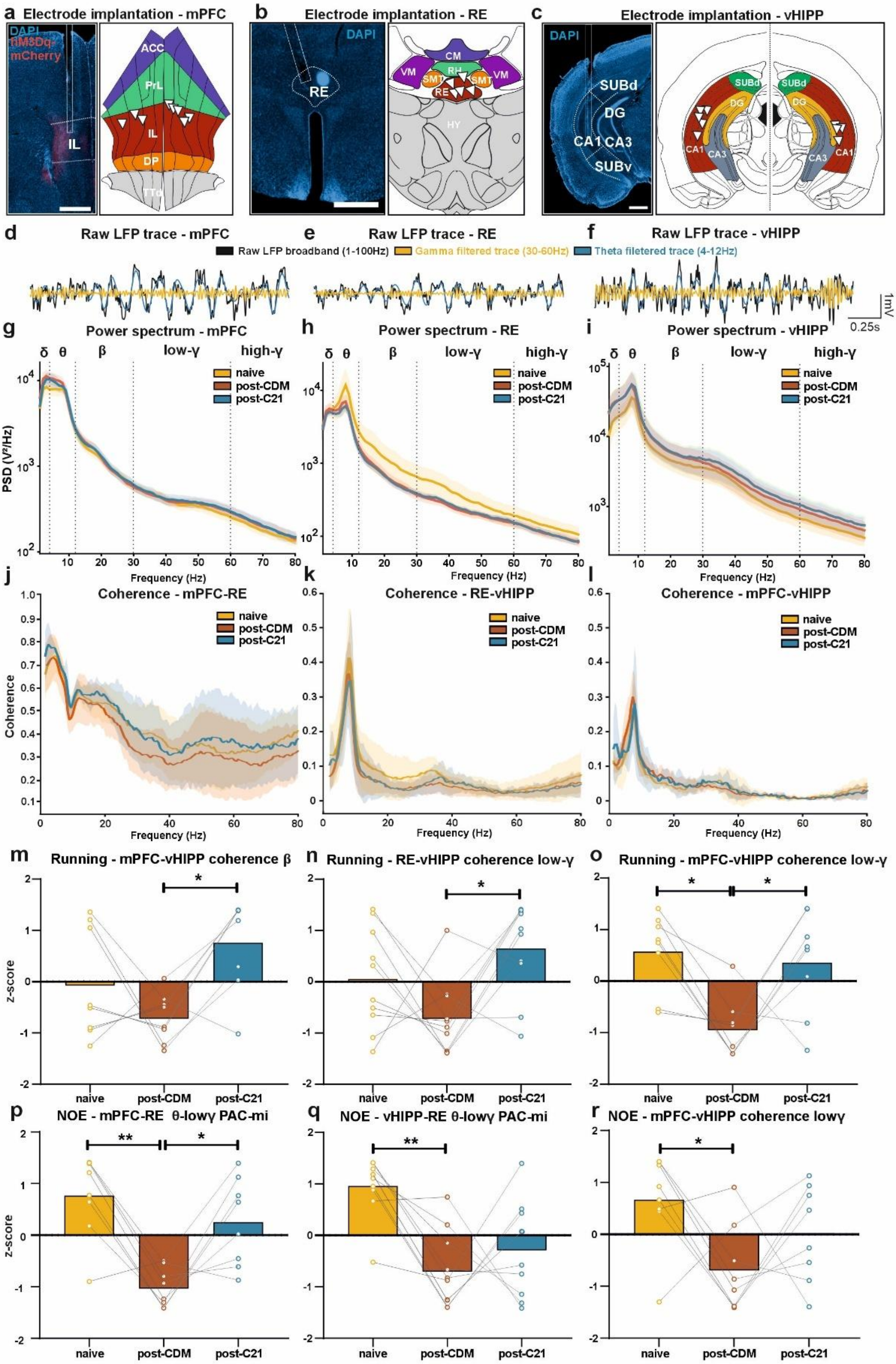

Supplementary Fig. 9 | a Left: representative image of an electrode placement and AAV injection in the mPFC; right: schematic representation of all electrodes implanted showing IL and surrounding areas. Scale bar: 500 µm. **b** Left: representative image of electrode placement in the RE; right: schematic representation of all electrodes implanted with surrounding thalamic nuclei indicated. Scale bar: 500 µm. **c** Left: representative image of electrode placement in the vHIPP; right: schematic representation of all electrodes implanted with hippocampal subregions indicated. Scale bar: 500 µm. **d-f** Representative raw LFP traces showing broadband signal (1–80 Hz, black), gamma-filtered trace (30–60 Hz, yellow) and theta-filtered trace (4–12 Hz, blue) recorded from the mPFC (**d**), RE (**e**), and vHIPP (**f**). **g–i** Power spectral density (PSD) across frequency bands for each recording period in the mPFC (**g**), RE (**h**) and vHIPP (**i**). **j–l** Coherence spectrum across conditions between mPFC and RE (**j**), RE and vHIPP (**k**) and mPFC and vHIPP (**l**). **m** mPFC–vHIPP β coherence during running (n=8 ipsilateral pair; CDM vs. naive: *P*=0.3854; CDM vs. C21: *P*=0.0269). **n** RE–vHIPP low-γ coherence during running (n=10 pair; CDM vs. naive: *P*=0.2288; CDM vs. C21: *P*=0.0219). **o** mPFC–vHIPP low-γ coherence during running (n=8 ipsilateral pair; CDM vs. naive: *P*=0.0152; CDM vs. C21: *P*=0.0352). **p** mPFC–RE θ–low-γ phase-amplitude coupling modulation index (PAC-MI) during NOE (n=8 pair; CDM vs. naïve: *P*=0.0021; CDM vs. C21: *P*=0.0208). **q** vHIPP-RE θ–low-γ PAC-MI during NOE (n=10 pair; CDM vs. naïve: *P*=0.0017; CDM vs. C21: *P*=0.5114). **r** mPFC–vHIPP low-γ coherence during NOE (n=8 ipsilateral pair; CDM vs. naive: *P*=0.0456; CDM vs. C21: *P*=0.3398). Statistical comparisons were performed using repeated measures one-way ANOVA with Dunnett's post-hoc test. Data are presented as mean ±SEM and the individual data points are depicted. **P*<0.05, ***P*<0.01, ****P*<0.001. Source data are provided as a Source Data file.
