# Supplementary material for: The mPFC-reuniens-hippocampus pathway links brain circuitry and neural plasticity in antidepressant response
Source: Nat Commun. 2026 Sep 1;17:9361. doi: 10.1038/s41467-026-77248-y (PMC13534506; doi:10.1038/s41467-026-77248-y)
Supplement: Supplementary file 2 [file 41467_2026_77248_MOESM2_ESM.pdf]

nature portfolio

Peer Review File

The mPFC-Reuniens-Hippocampus Pathway Links Brain Circuitry and Neural Plasticity in Antidepressant Response.

Corresponding Author: Dr Stefan Vestring

This file contains all reviewer reports in order by version, followed by all author rebuttals in order by version.

Version 0:

Reviewer comments:

Reviewer #1

(Remarks to the Author)
This compelling study aims to investigate the role of the infralimbic cortex (IL) in modulating synaptic plasticity within the hippocampus (HIP) in the context of stress-induced depression. Utilizing modern neurobiology techniques, the authors discovered that IL activation induces behaviorally associated neuroplasticity in the ventral hippocampus. Intriguingly, they further unraveled that the nucleus reuniens (RE) acts as a gate, forming a crucial link between the IL and the vHIP. This circuit, they suggest, might be implicated in depression and is potentially fundamental to the fast-acting antidepressant effects of ketamine. While the experiments appear well-executed and the manuscript is clearly written, certain assumptions warrant careful consideration.

1) Line 140 “To confirm the efficacy of the chemogenetic stimulation, we measured the mRNA expression of neuronal activation and plasticity-related immediate early genes (IEGs) using qRT–PCR after the administration of Compound C21 (0.75 mg/kg)…”

From which brain region was the mRNA expression measured?
In Figure 1c, d, and e, the progression of CDM is only shown in the FST. Why didn't the authors show the days in the sucrose preference test and the TST?
2) Line 176 “Using the three-chamber task, we measured sociability as the ratio of time spent exploring a novel social stimulus versus a novel object. IL stimulation significantly increased social preference in hM3Dq-expressing mice compared to mCherry control-expressing mice (Fig. 1j), suggesting enhanced sociability.”

Was the novel object presented in the Social Preference test the same novel object used in the NOE test?

3) Line 171 “To assess exploratory behavior, we conducted a novel object exploration (NOE) test”

What exactly does this behavioral assay measure? Is it memory?
Figure 1i, does this test quantify an know object vs a new object?
4) Line 173 “significantly increased the time spent exploring both objects compared to control mice”

5) Line 180-185 “Additionally, to verify DREADD specificity, we compared hM3Dq-expressing mice that received either C21-supplemented water or regular drinking water as a control these….”

These results are confusing in their current organization (supplementary figures 1 and 2). I recommend reorganizing and including them before the behavioral test data presented in Figure 1.

6) Supplementary figure 2 Does "+CTL" refer to drinking water?
7) Line 186 “To establish the specificity of the role of IL in the antidepressant effect of mPFC stimulation, we conducted parallel DREADD experiments targeting the prelimbic cortex (PrL),”

What evidence would indicate that the IL or the PrL are specifically activated?
Perhaps the naïve and CDM groups could also be included in the time/potential plots, in addition to the hM3Dq and mCherry

groups.

Additionally, it would be interesting (at least for supplementary data) to compare the LTP across the naïve, CDM, hM3Dq, and mCherry groups. For example, a comparison between the naïve and hM3Dq groups could reveal whether neuronal activation reverses or even overrides the depressive condition.

8) Supplementary figure 3, It would be interesting to show the PPR and the Rm in CDM mice compared to naïve mice. Furthermore, the implications of these results in the context of CDM and hM4Dq's effects on the presynaptic-postsynaptic mechanisms of LTP warrant discussion. It would also be valuable to discuss this assumption in light of published data (e.g., on CDM, depression, antidepressants, etc.).
9) Figure 3b, As the three mice represent movement, it would be fine to add a curved arrow below them to symbolize this.
10) Line 223 "The global peak amplitudes during full periods of immobility"
What is the global peak between -4s and 4s?
11) Line 244 "These data suggest that IL stimulation increases neuronal activity and the expression of plasticity related genes specifically in the vHIP, identifying this hippocampal subregion as a key downstream target of IL activation."
Please discuss these observations. For example, what is known about the projections between the IL, vHIP, and dHIP? Also, what connections are relevant in the context of specific behavioral responses? And consider using "HIPP" instead of "HIP"

12) Figure 4g, h. Since the anatomical location of the signal cannot be distinguished, including less zoomed microphotographs as shown in Figure 4f would be very useful.
13) It is not clear how the three-dimensional quantification of synaptic density was conducted.
14) Line 260 "In the mPFC, VGLUT2 and GAD65 density remained unchanged after overnight IL stimulation"
For this discussion, please provide more detail, including known data about the efferents from the mPFC to the vHIP.

15) Given that CaMKIIα-mCherry was not injected into the mPFC, why is a red signal present in Figure 5b?
16) Figure 5a (right) should indicate that the HIPP was analyzed using patch-clamp electrophysiology.
Line 296 "Consistent with previous findings16,31, ketamine (10 mg/kg) reversed CDM-induced immobility in control non-AAV-injected mice (Supplementary Fig. 6a)." while Supplementary figure 6 "Supplementary Fig. 6 | a PPR comparison pre- vs. post aLTP induction. Left: hM3Dq mPFC 134 + hM4Di RE mice treated with C21 overnight" Thus, there is a discrepancy between Supplementary Fig. 6 and the text.

17) Figure 6. The specific viral infections and their corresponding signal colors are indeed confusing. For instance, do the font colors of the construct names actually correspond with the expected signal colors? Including detailed information or a clear schema of the expected signals in a supplementary figure would be particularly useful. For example, illustrating a single retro-hSyn-Cre construct with two bifurcating arrows showing the anticipated color signal in the mPFC, and labeling the CamKIIa-Hm4d(Gi) construct in its corresponding signal color, could significantly improve understanding. Perhaps simply coloring the name of the fluorescent molecule within the full construct name would also enhance clarity. Furthermore, the presence of a DIO-hM4Di signal in the RE, given its injection solely in the mPFC, warrants explanation. Finally, what exactly does "Retro-cre" refer to in Figure 5b and 5c?"
18) Line 301 "ketamine restored hippocampal aLTP in control mice"
In which cell type or brain area were these experiments conducted?
19) Supplementary figure 7
It would be beneficial to further compare the PPR effect between groups and discuss the results.
20) For a more complete understanding of the IL-RE-vHIPP circuit, the authors should conduct retrograde experiments investigating the RE-vHIP and potentially the LI-vHIP communication.
21) Line 419 "Given that IL stimulation affects motivational, social, and anhedonia-like behaviors, which are highly dependent on limbic structures, our findings suggest that stimulation of this circuit might be a central "starting point" for antidepressant effects, potentially spreading to multiple downstream targets."
Despite this interesting point, ketamine's action involves multiple brain regions concurrently. Therefore, what might be observed if ketamine were directly administered to the IL?
22) Most of the ketamine studies had not used the CDM model, therefore, what the authors would expect if they added a naïve control?
23) Given that most ketamine studies did not employ the CDM model, what expectations would the authors have if a naïve control were included?
24) Line 225 "These findings demonstrate that IL activation bidirectionally modulates HIP activity during behavioral transitions, increasing excitatory neuron responses during struggle and further suppressing these responses during immobility, with concurrent changes in inhibitory neuron dynamics. This increased state-dependent dynamics suggests that IL activation improves hippocampal "gain", potentially contributing to its antidepressant effects"
Line 428 "LTP measurements in hippocampal slices provide a rather "binary" readout of associative plasticity: present in healthy condition, it is abolished in a pathological state and restored after antidepressant treatment. Our manipulations of the IL → RE → HIP circuit reflect this binary nature, as activating this circuit "turns on" hippocampal LTP while disrupting it prevents this effect, maintaining LTP in an "off" state.

Regarding these observations, it appears that the activation of the IL induces higher neuronal flexibility in the vHIPP. However, the evaluation of neuronal dynamics in the RE is missing; including this experiment would be beneficial.

25) Line 435 “Altogether, our study unifies two prominent theoretical frameworks for understanding depression pathophysiology, namely, the circuitry hypothesis and the neuroplasticity hypothesis. We identified the IL → RE → vHIP circuit as a central and converging network that regulates the response to antidepressant therapy and plasticity in the hippocampus”

Have neuroimaging studies provided evidence of a specific connectome pathway supporting this circuit?

(Remarks on code availability)

Reviewer #2

(Remarks to the Author)
Summary: This is an excellent series of experiments that isolates the IL>RE circuitry primarily using a hM3Dq DREADDs, sometimes in combination with retro-Cre. Their goal was to analyze the properties of an IL> RE> vHip circuit as related to antidepressants. Specifically, they measure how modulations of IL or IL>RE affect depression-like behaviors, hippocampal LTP, hippocampal synaptic activity during behavioral transitions, hippocampal IEGs, hippocampal synapses, and ketamine outcomes. Generally, they find that IL stimulation improves or recovers depression-like outcomes, and that RE inhibition blocks some of those improvements. Overall, this is a solid paper that is well-written. However, I have some significant concerns about the data presentation and a modest concern about the interpretation of the results.

Major Concern: The histological analysis of the distribution of hM3Dq, hM4Di, mCherry, GCaMP8m, jRGECO1a, and the dual-labeled retro-CRE/hM4D in the IL, PrL, and RE is lacking and critical to the interpretation of the experiments. Additionally, the fiber photometry implants should be localized histologically in all animals. This is particularly critical as C21 is always administered systemically and IL targets a very large number of structures, including those near the vHC, such as the amygdala and the entorhinal cortex, that likely play roles in depression. As it stands, only some representative slices are shown for some experimental groups, often not the DREADD groups nor the GCaMP8m group. For example, Fig6, shows the best images to critically evaluate the circuit isolation. The photomicrograph shows retro-CRE cells throughout the entire mPFC, including PrL, which projects strongly to RE, and this labeling circles around all deep cortical layers. The distribution of the dual-labeled cells should be shown to demonstrate that these cells are only in IL in the experiment. Likewise, in Fig. 4d, there is an example slice for RE in which the hM4Di cells appear to concentrate in RE, but there are also many cells outside of RE. Therefore, it’s unclear whether the mouse had hM4Di restricted to RE, and there is no information available about the other mice included in the experiment. The histological issue is present in all experiments and there is no description of the histology in the methods.

Specific Questions/Comments:

1. Please identify the regions that distinguish the salience network. Line 98

2. “…reverse depressive-like phenotypes…” is much too strong a characterization of the effect of all the listed treatments and is not appropriate for a general audience. All these treatments have modest effects on depression scales, and all are temporary. This should be described carefully so no one acts on the line as stated. Lines 102-104

3. The basolateral amygdala (BLA) and intercalated cells are key targets of long-range infralimbic cortex (IL) projections that are thought to play a role in the expression of fear memories in rodents (circuit methods), and potentially in human depression (imaging methods). Was the IL BLA considered for any of the outcomes? This is relevant in both the introduction and discussion to properly frame the interpretation of circuit manipulation outcomes and their alternatives. Lines 104-107

4. “…the correlates of synaptic plasticity…” These studies do not establish a correlation between the evoked potentials and synaptic plasticity. Instead, the evoked potential outcomes could be attributed to synaptic plasticity. Still, there are physiological alternatives to long-term potentiation (LTP) in these paradigms, and there is no clear evidence of hippocampal involvement. Line 114

5. Several IL circuits may contribute to reduced anxiety and increased sociability. In this regard, it’s great that the authors added the PL control in the DREADD experiments, but the amygdala is also an obvious target for these experiments; it’d be beneficial to know what's happening there throughout the experiments.

6. Is there a quantification of the distribution of AAV-hM3d+ cells for PL vs. IL surgical targets?

7. What do the sample sizes mean in the LTP experiments? Cells, slices or mice?

8. Do IL neurons synapse on inhibitory neurons, excitatory neurons, or both? Is there any more specificity to which types of cells IL targets to help explain the behavior-related calcium dynamics?

9. There is text throughout Fig4 that is too small.

10. It would be important to look at the BLA and central amygdala regions for changes in IEGs.

11. How were images selected for the IEG and synaptic quantification in Fig4?

12. Line 288-289, Do these experiments reveal anything about the IL>vHC or RE vHC component of the proposed IL RE vHC circuit in antidepressant-like effects? It appears to demonstrate only the IL > RE component and IL modulation of vHC.

13. Lines 304-306. I don't follow the logic that ketamine provides insight into the vHC circuitry. While it seems plausible, and maybe even likely, that the NMDA receptors in the vHC play a role in the antidepressive effects of ketamine. While these experiments isolate IL>RE pathways (although RE projecting neurons located in IL likely target many structures), there is no isolation of the RE>vHC pathway.

14. What is the evidence for vHC pathways in the circuit manipulations? The authors show several functional correlations (e.g., IEGs), but not the functional role for the RE>vHC pathway. Instead, I would say the IL-RE pathway modulates vHC. Line 321, also Line 430, 437

15. The description of the morphological analysis needs more information about the acquisition hardware (e.g., confocal microscope), how slices were selected, how ROIs were chosen, etc.

16. The histology is not described in the methods.

(Remarks on code availability)

Reviewer #3

(Remarks to the Author)
In this manuscript, Veleanu et al. present novel evidence implicating the mPFC–RE–CA1 circuit in the antidepressant response to chronic stress in mice. Specifically, they demonstrate that overnight chemogenetic activation of hM3Dq-expressing excitatory neurons in the infralimbic cortex (IL) of the mPFC induces antidepressant-like behavioral effects, rescues stress-induced deficits in CA3–CA1 LTP in hippocampal slices, alters calcium dynamics in the ventral CA1 during the tail suspension test (TST), and modifies gene expression and Vglut2-positive puncta density in the ventral hippocampus. Importantly, they show that the nucleus reuniens (RE) is required for the antidepressant effect of IL stimulation and that selective chemogenetic inhibition of the mPFC–RE pathway blocks the antidepressant effect of ketamine.

While this study offers a compelling circuit-level framework connecting prefrontal modulation to hippocampal plasticity in depression, several conceptual and technical issues need to be addressed to clarify the mechanistic basis of the findings:

1. Unclear nature of the IL perturbation: The authors claim that activation of IL excitatory neurons produces antidepressant effects, yet the effect of this perturbation on network activity is uncharacterized. Previous theoretical and experimental studies demonstrate that cortical circuits represent inhibition-stabilized networks, whereas cortical excitation can result in paradoxical decreases in activity, depending on the extent and nature of the perturbation (eg PMID: 32598278). Without direct measurements of IL spiking in response to the perturbation, the interpretation of downstream effects remains speculative.

2. Time course of chemogenetic effects: The manuscript lacks a detailed assessment of the acute versus overnight effects of C21. Acute intraperitoneal injections and chronic delivery via drinking water may differ significantly in their impact. It is unclear whether the behavioral and plasticity effects are due to the direct impact of IL activation or compensatory mechanisms arising from prolonged stimulation.

3. Fiber photometry results are difficult to interpret: State-dependent modulation of hippocampal Ca^{2+} signals (increased during struggle, decreased during immobility) could reflect changes in motor behavior rather than circuit engagement per se. Without monitoring other behavioral states (e.g., quiet wakefulness, active exploration in the home cage), and ideally without single-unit resolution, it's challenging to attribute the observed changes to antidepressant effects rather than confounds like arousal or movement.

4. Lack of direct measurements from IL→CA1 or RE→CA1 pathways: The authors propose that IL stimulation modulates hippocampal LTP via the RE. However, the synaptic and cellular mechanisms remain poorly defined. Electrophysiological recordings from IL–CA1 and RE–CA1 projections would greatly strengthen the mechanistic claims. Recordings from these pathways have been preveriously performed in vivo (e.g. PMID: 9204945, 37919286) and in brain slices (PMID: 32065917). Furthermore, it s unclear whether the chemogenetic manipulation that rescued LTP was performed in vivo (i.e., prior to slice preparation) or ex vivo (i.e., in the slice).

5. Effect of mPFC–RE inhibition on functional connectivity: The strong behavioral effects of mPFC–RE inhibition are compelling. However, the physiological consequence of this manipulation is not addressed. fEPSP recordings from the mPFC–RE pathway could reveal whether synaptic strength or short-term plasticity is altered by this intervention, and how it relates to behavior.

6. Circuit dysfunction in depression is not well characterized: The authors argue that the IL–RE–vHIP circuit is involved in antidepressant responses, yet provide limited data on how this circuit is dysregulated in the CDM depression model.

Although hippocampal LTP is shown to be abolished by chronic stress, baseline activity or synchrony in the IL, RE, or CA1 is not assessed. This gap limits the interpretability of how the circuit is pathologically altered and how IL stimulation restores function.

7. Broader role of RE in cognitive function and disease: Chemogenetic and electrical manipulations of the RE have been shown to affect working memory and contribute to hyperexcitability in Alzheimer's models. It would be valuable for the authors to briefly discuss these findings in light of their results, particularly regarding potential off-target or pleiotropic effects of RE inhibition.

(Remarks on code availability)

Version 1:

Reviewer comments:

Reviewer #1

(Remarks to the Author)

The authors have addressed the concerns satisfactorily. I have a few remaining minor observations for your consideration

1) Line 602 “Behavior was recorded for 10 min, and total distance traveled and time spent in the center area were analyzed.” How was it analyzed?
2) Line 639-645 Which session included the quantification of sucrose licking behavior?
3) Line 140 (Fig. 1a-b; Supplementary Fig. 7a-c).
Perhaps you should reorder the figures according to their order of appearance
4) Line 656-7 “Reverse transcription was performed with 1 mg of total RNA using M-MLV reverse transcriptase (Promega).” Is 1 mg correct? Or should it be 1 μg (microgram)
5) In Figure 3D, shouldn't the results at -4 s and -2 s be the same as those shown in Figure 3C at 2 s and 4 s (specifically the higher struggle/delta F/F levels post-C21)?
6) Lines 239-243 “These findings demonstrate that IL activation bidirectionally modulates HIPP activity during behavioral transition in the TST, but not homecage exploration, increasing excitatory neuron responses during struggle and further suppressing these responses during immobility, with concurrent changes in inhibitory neuron dynamics. This increased state-dependent dynamics suggests that IL activation improves hippocampal “gain” in stress-related behaviour, potentially contributing to its antidepressant effects.”

This is an interesting finding; how might this translate to a human social context?
7) Line 250 Could you please define 'IEGs' ?
8) Figure 5e, What is the interpretation for the null effect of mPFC activation on OFT performance? One would expect the results to be consistent with those shown in Figure 1h

9) Figure 6f, In this experiment, the RE inhibits the IL, which in turn prevents the IL from activating the RE (rather than the mPFC-mediated inhibition of the RE). Wouldn't it be more precise for Figure 6f to explicitly show this—perhaps by indicating the experimental inhibition of the mPFC and the resulting reduction in RE activity?

10) Figure 7f A similar observation was made: despite mPFC activation, when the RE is inhibited, there is no Hippocampal activation (LTP). Instead of an inhibition of the Hippocampus. This follows the same concept seen in Figure 7i: RE inhibition does not directly inhibit the HIPP; rather, RE activity is required to activate the HIPP.
11) Why was LTP analyzed in one experiment, while the FST was used as the output in the second?"
12) Line 402 “The mPFC does not send direct excitatory projections to the HIPP; its primary excitatory route is relayed through the RE, which innervates preferentially intermediate and ventral CA1 and the subiculum with comparatively sparse projections to the dHIPP”

This sentence is a little confusing I suggest as follows “The mPFC does not send direct excitatory projections to the HIPP; its primary excitatory route is relayed through the RE, which preferentially innervates the intermediate and ventral CA1 and the subiculum, with comparatively sparse projections to the dHIPP”

13) Figure 8: While the model is functional, would it be more elegant to show sagittal sections instead of coronal ones?
14) Line 417 “We provide compelling evidence that chronic stress-induced impairments in hippocampal LTP can be fully reversed by stimulation of the IL”
Please refer to the figures
15) Line 424 “between active and passive coping behavioral states”
Please refer to the figures
16) Line 436 “modulate RE firing patterns”
Please refer to the figures
17) Line 511 “DTI-based tractography in SCC DBS patients has identified three critical white matter bundles”
18) Diffusion tensor imaging (DTI)

(Remarks on code availability)

Reviewer #2

(Remarks to the Author)
The authors have addressed all my concerns.

(Remarks on code availability)

Reviewer #3

(Remarks to the Author)
The authors have comprehensively addressed my main previous concerns. The additional experiments and data analyses significantly strengthen the manuscript's primary claims. From my perspective, the manuscript is now ready for publication.

(Remarks on code availability)

Open Access This Peer Review File is licensed under a Creative Commons Attribution 4.0 International License, which permits use, sharing, adaptation, distribution and reproduction in any medium or format, as long as you give appropriate credit to the original author(s) and the source, provide a link to the Creative Commons license, and indicate if changes were made.
In cases where reviewers are anonymous, credit should be given to 'Anonymous Referee' and the source.
The images or other third party material in this Peer Review File are included in the article's Creative Commons license, unless indicated otherwise in a credit line to the material. If material is not included in the article's Creative Commons license and your intended use is not permitted by statutory regulation or exceeds the permitted use, you will need to obtain permission directly from the copyright holder.
To view a copy of this license, visit https://creativecommons.org/licenses/by/4.0/

REVIEWER COMMENTS

Reviewer #1 (Remarks to the Author):

This compelling study aims to investigate the role of the infralimbic cortex (IL) in modulating synaptic plasticity within the hippocampus (HIP) in the context of stress-induced depression. Utilizing modern neurobiology techniques, the authors discovered that IL activation induces behaviorally associated neuroplasticity in the ventral hippocampus. Intriguingly, they further unraveled that the nucleus reuniens (RE) acts as a gate, forming a crucial link between the IL and the vHIP. This circuit, they suggest, might be implicated in depression and is potentially fundamental to the fast-acting antidepressant effects of ketamine. While the experiments appear well-executed and the manuscript is clearly written, certain assumptions warrant careful consideration.

Author's Response: We thank Reviewer 1 for the thorough evaluation of our manuscript and for the constructive and thoughtful comments. We appreciate the time and effort invested in the detailed review, which has helped us to improve the clarity and presentation of the manuscript. Below we respond to each point raised by the reviewer.

1) Line 140 "To confirm the efficacy of the chemogenetic stimulation, we measured the mRNA expression of neuronal activation and plasticity-related immediate early genes (IEGs) using qRT–PCR after the administration of Compound C21 (0.75 mg/kg)…"

From which brain region was the mRNA expression measured?

Author's Response: We apologize for the lack of clarity. The IEG mRNA expression was measured from microdissected ventral medial prefrontal cortex tissue enriched for the infralimbic cortex. This information has now been clarified in the revised manuscript (line 647-648)

In Figure 1c, d, and e, the progression of CDM is only shown in the FST. Why didn't the authors show the days in the sucrose preference test and the TST?

Author's Response: In the CDM protocol, the five days of swimming constitute the stress induction of depression-like phenotype. Immobility from Day 1 to Day 5 is tracked to verify successful induction of depression-like behaviour at the cohort level. Outcome measures (forced swim test, tail suspension test, or sucrose preference test) are then performed in separate cohorts. The SPT was measured at baseline (naïve state, before CDM), after CDM induction (post-rest), and after overnight C21

treatment, but longitudinal day-by-day tracking during CDM is not feasible with this assay. In order to avoid exposing the mice to the same test multiple times, TST was performed only after C21 treatment. TST data showing CDM progression before CDM (naïve) and after CDM induction is presented in the Supplementary Fig S2m. We have clarified this procedure in the Methods section of the revised manuscript (lines 583–594).

2) Line 176 "Using the three-chamber task, we measured sociability as the ratio of time spent exploring a novel social stimulus versus a novel object. IL stimulation significantly increased social preference in hM3Dq-expressing mice compared to mCherry control-expressing mice (Fig. 1j), suggesting enhanced sociability."
Was the novel object presented in the Social Preference test the same novel object used in the NOE test?

Author's Response: We agree that this point may not have been sufficiently clear in the original manuscript. The objects used in the social preference test and the NOE test were different, and the experiments were conducted in separate cohorts of mice to ensure exposure only to previously unseen objects. This has now been clarified in the revised manuscript (line 614).

3) Line 171 "To assess exploratory behavior, we conducted a novel object exploration (NOE) test"
What exactly does this behavioral assay measure? Is it memory?
Figure 1i, does this test quantify an know object vs a new object?

Author's Response: This comment of the reviewer made clear that due to space constraints, we had not sufficiently described this experiment. The novel object exploration (NOE) test used in this study does not assess memory. In contrast to the novel object recognition (NOR) paradigm, which compares exploration of a familiar and a novel object after a familiarization phase, the NOE presents two novel, previously unseen objects simultaneously. This paradigm therefore measures exploratory drive and motivation to investigate novelty rather than memory performance. The reference to "both objects" refers to the cumulative exploration time of these two novel objects. The manuscript has been revised accordingly to clearly distinguish this paradigm from memory-related tasks (lines 174-177).

4) Line 173 "significantly increased the time spent exploring both objects compared to control mice"
5) Line 180-185 "Additionally, to verify DREADD specificity, we compared hM3Dq-expressing mice that received either C21-supplemented water or regular drinking water as a control these...."

These results are confusing in their current organization (supplementary figures 1 and 2). I recommend reorganizing and including them before the behavioral test data presented in Figure 1.

Author's Response: We thank the reviewer for the suggestion and agree that the original organization was suboptimal. We have reorganized the supplementary figures so that DREADD validation is presented first: Supplementary Fig. S1 now provides direct electrophysiological evidence of hM3Dq efficacy via patch-clamp recordings in IL neurons, Supplementary Fig. S2 presents the DREADD specificity behavioral controls (hM3Dq +C21 vs. hM3Dq without C21) along with additional behavioral characterization, and IEG expression data have been moved to Supplementary Fig. S5.

6) Supplementary figure 2 Does "+CTL" refer to drinking water?

Author's Response: We apologize again for the lack of clarity. Yes, "+CTL" referred to regular drinking water without C21. We acknowledge the ambiguity and have relabeled this condition as "+Vehicle (water)" in the revised Supplementary Fig. S2 legend for clarity.

7) Line 186 "To establish the specificity of the role of IL in the antidepressant effect of mPFC stimulation, we conducted parallel DREADD experiments targeting the prelimbic cortex (PrL),"

What evidence would indicate that the IL or the PrL are specifically activated?

Author's Response: We now provide comprehensive histological validation of viral expression specificity for all experimental groups (new Supplementary Fig. S7). Viral expression was quantified by registering widefield images to the Allen Brain Atlas using BigWarp and measuring the proportion of fluorescence across defined mPFC subregions (IL, PrL, ACC, DP, TTd).

Perhaps the naïve and CDM groups could also be included in the time/potential plots, in addition to the hM3Dq and mCherry groups.

Additionally, it would be interesting (at least for supplementary data) to compare the LTP across the naïve, CDM, hM3Dq, and mCherry groups. For example, a comparison between the naïve and hM3Dq groups could reveal whether neuronal activation reverses or even overrides the depressive condition.

Author's Response: The naïve and CDM groups and the hM3Dq and mCherry groups are presented in separate plots (Fig. 2c, d and Fig. 2e, f) for more clarity. To facilitate direct comparison across all conditions as suggested by the reviewer, we have added a multi-condition overlay in Supplementary Fig. 3m, showing aLTP levels across naïve, CDM, IL-mCherry + C21 overnight, IL-hM3Dq + C21 overnight, and IL-hM3Dq + C21

acute groups. These data suggest that overnight IL stimulation partially restores aLTP rather than overriding the depressive-like condition, although direct comparison between these groups should be interpreted with caution as naïve mice did not undergo stereotactic surgery, whereas hM3Dq mice received bilateral viral injections.

8) Supplementary figure 3, It would be interesting to show the PPR and the Rm in CDM mice compared to naïve mice. Furthermore, the implications of these results in the context of CDM and hM4Dq's effects on the presynaptic-postsynaptic mechanisms of LTP warrant discussion. It would also be valuable to discuss this assumption in light of published data (e.g., on CDM, depression, antidepressants, etc.).

Author's Response: We thank the reviewer for pointing this out. The corresponding analyses have now been included in Supplementary Fig. S3, and the implications are addressed in the revised manuscript (lines 208-209).

9) Figure 3b, As the three mice represent movement, it would be fine to add a curved arrow below them to symbolize this.

Author's Response: We thank the reviewer for the suggestion. We have added a curved arrow around the mice in Fig. 3 to indicate movement as suggested.

10) Line 223 "The global peak amplitudes during full periods of immobility"
What is the global peak between -4s and 4s?

Author's Response: We apologize for the lack of clarity. The "global peak amplitude" refers to the mean amplitude of Ca^{2+} transients detected across the entire cumulative period of immobility or struggle. Transients were identified using the find_peaks function from the scipy library, and their amplitudes were averaged per behavioral state for each animal. We have clarified this definition in the revised Methods section (lines 870-873).

11) Line 244 "These data suggest that IL stimulation increases neuronal activity and the expression of plasticity related genes specifically in the vHIP, identifying this hippocampal subregion as a key downstream target of IL activation."
Please discuss these observations. For example, what is known about the projections between the IL, vHIP, and dHIP? Also, what connections are relevant in the context of specific behavioral responses? And consider using "HIPP" instead of "HIP"

Author's Response: We appreciate the reviewer's attention to this detail. We have expanded the Discussion to address this point and now outline the known projections between the infralimbic cortex, nucleus reuniens, and ventral versus dorsal

hippocampus, and how these circuit relationships may relate to the behavioural responses observed in our study (lines 402–415). In addition, HIP has been corrected to HIPP throughout the manuscript.

12) Figure 4g, h. Since the anatomical location of the signal cannot be distinguished, including less zoomed microphotographs as shown in Figure 4f would be very useful.

Author's Response: We thank the reviewer for the suggestion. As requested, we have added lower-magnification microphotographs to Figure 4g and 4h, consistent with the format used in Figure 4f, allowing clear identification of the anatomical location of the signal.

13) It is not clear how the three-dimensional quantification of synaptic density was conducted.

Author's Response: We apologize for the lack of clarity. A detailed description of this procedure has been added to the Methods section of the revised manuscript (lines 824–842).

14) Line 260 "In the mPFC, VGLUT2 and GAD65 density remained unchanged after overnight IL stimulation"
For this discussion, please provide more detail, including known data about the efferents from the mPFC to the vHIP.

Author's Response: We thank the reviewer for the suggestion. We have expanded the Discussion to address these points (lines 398–407).

15) Given that CaMKIIα-mCherry was not injected into the mPFC, why is a red signal present in Figure 5b?

Author's Response: We apologize for the misleading scheme in the original figure. The red signal visible in Figure 5b corresponds to the hM3Dq–mCherry fusion protein expressed under the CaMKIIα promoter in the IL, where the mCherry fluorophore allows visualization of DREADD-expressing neurons. This has now been clarified in the revised figure.

16) Figure 5a (right) should indicate that the HIPP was analyzed using patch-clamp electrophysiology.

Author's Response: We thank the reviewer for pointing this out. We have added a patch-clamp electrophysiology symbol to the schematic in Figure 5a to indicate that hippocampal slices were used for electrophysiological recordings.

Line 296 “Consistent with previous findings16,31, ketamine (10 mg/kg) reversed CDM-induced immobility in control non-AAV-injected mice (Supplementary Fig. 6a).” while Supplementary figure 6 “Supplementary Fig. 6 | a PPR comparison pre- vs. post aLTP induction. Left: hM3Dq mPFC 134 + hM4Di RE mice treated with C21 overnight” Thus, there is a discrepancy between Supplementary Fig. 6 and the text.

Author’s Response: We thank the reviewer for noticing this apparent mistake in supplementary figure numbering and apologize for it; The supplementary figures have been comprehensively renumbered in the revised manuscript to ensure consistency, and the ketamine behavioral data are now referenced as Supplementary Fig. 8i.

17) Figure 6. The specific viral infections and their corresponding signal colors are indeed confusing. For instance, do the font colors of the construct names actually correspond with the expected signal colors? Including detailed information or a clear schema of the expected signals in a supplementary figure would be particularly useful. For example, illustrating a single retro-hSyn-Cre construct with two bifurcating arrows showing the anticipated color signal in the mPFC, and labeling the CamKIIa-Hm4d(Gi) construct in its corresponding signal color, could significantly improve understanding. Perhaps simply coloring the name of the fluorescent molecule within the full construct name would also enhance clarity.

Author’s Response: We acknowledge that the intersectional viral strategy was insufficiently explained in the original submission, and we apologize for the presentation issues. We have substantially revised Figures 5 and 6 to address this. Construct labels now explicitly include the fluorophore name (e.g., "DIO-hM4Di-mCherry", "retro-Cre-GFP") and are color-coded to match the corresponding group more clearly. Although matching the labeling of the construct with the fluorophore would be ideal, it is indeed difficult to apply here, since most of the constructs contain mCherry. We have separated the RE inhibition and PFC→RE inhibition schemes in Figure 6a for improved clarity.

Furthermore, the presence of a DIO-hM4Di signal in the RE, given its injection solely in the mPFC, warrants explanation.

Author’s Response: We agree that this point is confusing without explanation. In the original submission, the red fluorescent signal present in the RE despite the DIO-hM4Di construct being injected exclusively in the mPFC corresponded to the axonal projections of recombined IL→RE neurons. The Cre-mediated recombination occurs in the mPFC soma of RE-projecting neurons, and the resulting hM4Di-mCherry fusion protein is transported anterogradely to axonal projections in the RE. In the revised Figure 6, the histology panel now focuses on the mPFC injection site, showing the

critical site of recombination and the co-localization of DIO-hM4Di-mCherry and retro-CRE-GFP in IL neurons (Fig. 6b), which is the most informative view for evaluating the specificity of the intersectional approach. The RE injection site histology is presented in Figure 5c, and the comprehensive histological quantification of viral expression across all experimental groups, including this condition, is provided in Supplementary Fig. 7.

Finally, what exactly does “Retro-cre” refer to in Figure 5b and 5c?"

Author’s Response: We apologize for the apparent mistake. "Retro-Cre" label that appeared in Figure 5b and 5c was erroneous; this construct is specific to the intersectional experiment presented in Figure 6. This has been corrected in the revised figures.

18) Line 301 “ketamine restored hippocampal aLTP in control mice”
In which cell type or brain area were these experiments conducted?

Author’s Response: We thank the reviewer for highlighting this. These experiments were conducted in CA1 pyramidal neurons in acute hippocampal slices (CA3–CA1 synapse), which has now been clarified in the revised manuscript (lines 322–326).

19) Supplementary figure 7
It would be beneficial to further compare the PPR effect between groups and discuss the results.
Author’s Response: We thank the reviewer for the suggestion. We have now included the requested comparison of the PPR between groups (Supplementary Fig. 3n) and addressed the implications of these findings in the revised manuscript (lines 208–209).

20) For a more complete understanding of the IL-RE-vHIPP circuit, the authors should conduct retrograde experiments investigating the RE-vHIP and potentially the LI-vHIP communication.

Author’s Response: We fully agree with the reviewer that functional demonstration of the RE→HIPP segment was essential to support the three-node circuit model. We have conducted new series of experiments with selective chemogenetic inhibition of RE→HIPP projections, that blocked mPFC stimulation-induced restoration of hippocampal aLTP and abolished the antidepressant effects of ketamine. These data have been presented in a new main Figure 7 and in the manuscript (lines 330-347).

21) Line 419 “Given that IL stimulation affects motivational, social, and anhedonia-like behaviors, which are highly dependent on limbic structures, our findings suggest that stimulation of this circuit might be a central "starting point" for antidepressant effects,

potentially spreading to multiple downstream targets."
Despite this interesting point, ketamine's action involves multiple brain regions concurrently. Therefore, what might be observed if ketamine were directly administered to the IL?

Author's Response: We agree with the reviewer that ketamine likely acts across multiple brain regions; however, previous work has shown that local ketamine infusion into the mPFC is sufficient to produce antidepressant-like effects (PMID: 26056286). We have added a brief discussion of this point in the revised manuscript (lines 463–465).

22) Most of the ketamine studies had not used the CDM model, therefore, what the authors would expect if they added a naïve control?
23) Given that most ketamine studies did not employ the CDM model, what expectations would the authors have if a naïve control were included?

Author's Response: We appreciate this interesting and thoughtful point. Our study was designed to examine the restoration of hippocampal plasticity and behaviour in a pathological state induced by the CDM, which abolishes aLTP and induces depressive-like behaviours. In naïve mice, where plasticity and behaviour are intact, ketamine would instead test enhancement of an already functional system; while this is certainly an interesting question, it represents a different experimental context and is beyond the scope of the present study.

24) Line 225 "These findings demonstrate that IL activation bidirectionally modulates HIP activity during behavioral transitions, increasing excitatory neuron responses during struggle and further suppressing these responses during immobility, with concurrent changes in inhibitory neuron dynamics. This increased state-dependent dynamics suggests that IL activation improves hippocampal"gain", potentially contributing to its antidepressant effects"
Line 428 "LTP measurements in hippocampal slices provide a rather"binary" readout of associative plasticity: present in healthy condition, it is abolished in a pathological state and restored after antidepressant treatment. Our manipulations of the IL → RE → HIP circuit reflect this binary nature, as activating this circuit "turns on" hippocampal LTP while disrupting it prevents this effect, maintaining LTP in an "off" state.

Regarding these observations, it appears that the activation of the IL induces higher neuronal flexibility in the vHIPP. However, the evaluation of neuronal dynamics in the RE is missing; including this experiment would be beneficial.

Author's Response: We agree with the reviewer that assessing RE dynamics is important and have now addressed this experimentally through longitudinal multi-site LFP recordings from the mPFC, RE, and vHIPP. Theta–gamma phase-amplitude coupling between the mPFC and RE was disrupted by CDM and restored by IL activation during novel object exploration, showing that the RE is functionally engaged within the circuit and that its coupling with the PFC is sensitive to both the depressive-like state and treatment. The corresponding data are presented in the new Supplementary Figure 9 (lines 348-355).

25) Line 435 "Altogether, our study unifies two prominent theoretical frameworks for understanding depression pathophysiology, namely, the circuitry hypothesis and the neuroplasticity hypothesis. We identified the IL → RE → vHIP circuit as a central and converging network that regulates the response to antidepressant therapy and plasticity in the hippocampus"
Have neuroimaging studies provided evidence of a specific connectome pathway supporting this circuit?
Author's Response: We appreciate this important translational point. Indeed, multiple lines of human evidence support the existence of a connectome pathway homologous to the IL → RE → vHC circuit described in our manuscript. We have now addressed this point in the Discussion, where we outline available neuroimaging and connectome evidence supporting our model in humans (lines 509–523).

Reviewer #2 (Remarks to the Author):

Summary: This is an excellent series of experiments that isolates the IL>RE circuitry primarily using a hM3Dq DREADDs, sometimes in combination with retro-Cre. Their goal was to analyze the properties of an IL> RE> vHip circuit as related to antidepressants. Specifically, they measure how modulations of IL or IL>RE affect depression-like behaviors, hippocampal LTP, hippocampal synaptic activity during behavioral transitions, hippocampal IEGs, hippocampal synapses, and ketamine outcomes. Generally, they find that IL stimulation improves or recovers depression-like outcomes, and that RE inhibition blocks some of those improvements. Overall, this is a solid paper that is well-written. However, I have some significant concerns about the data presentation and a modest concern about the interpretation of the results.

Author's Response: We thank the reviewer for the positive evaluation of our work and for the constructive and detailed feedback regarding the presentation and interpretation of the data. In particular, we agree that the comments concerning the histological validation were very valuable, and we believe that incorporating these

suggestions has substantially improved the clarity and rigor of the manuscript. The specific concerns are addressed below.

Major Concern: The histological analysis of the distribution of hM3Dq, hM4Di, mCherry, GCaMP8m, jRGECO1a, and the dual-labeled retro-CRE/hM4D in the IL, PrL, and RE is lacking and critical to the interpretation of the experiments. Additionally, the fiber photometry implants should be localized histologically in all animals. This is particularly critical as C21 is always administered systemically and IL targets a very large number of structures, including those near the vHC, such as the amygdala and the entorhinal cortex, that likely play roles in depression. As it stands, only some representative slices are shown for some experimental groups, often not the DREADD groups nor the GCaMP8m group. For example, Fig6, shows the best images to critically evaluate the circuit isolation. The photomicrograph shows retro-CRE cells throughout the entire mPFC, including PrL, which projects strongly to RE, and this labeling circles around all deep cortical layers. The distribution of the dual-labeled cells should be shown to demonstrate that these cells are only in IL in the experiment. Likewise, in Fig. 4d, there is an example slice for RE in which the hM4Di cells appear to concentrate in RE, but there are also many cells outside of RE. Therefore, it's unclear whether the mouse had hM4Di restricted to RE, and there is no information available about the other mice included in the experiment. The histological issue is present in all experiments and there is no description of the histology in the methods.

Author's Response: We agree that the presentation of the histological validation in the original version was not sufficiently clear. We have now addressed this by adding comprehensive histological analyses across all experimental groups, including quantification of viral expression and atlas-based mapping of fiber photometry implant locations. These data are now shown in Supplementary Figure 7 and Supplementary Figure 4a, and the full procedure is described in the Methods section of the revised manuscript (lines 874–904).

Specific Questions/Comments:
1. Please identify the regions that distinguish the salience network. Line 98
Author's Response: We thank the reviewer for the suggestion. The text has been revised to specify: the salience network, which includes the *anterior insula and dorsal anterior cingulate cortex*. This has been added to the introduction (lines 97-98).

2. "...reverse depressive-like phenotypes..." is much too strong a characterization of the effect of all the listed treatments and is not appropriate for a general audience. All these treatments have modest effects on depression scales, and all are temporary. This should be described carefully so no one acts on the line as stated. Lines 102-104

Author's Response: We agree with the reviewer that "reverse" overstates the clinical evidence. We have replaced this with "ameliorate depressive-like phenotypes" (line 105).

3. The basolateral amygdala (BLA) and intercalated cells are key targets of long-range infralimbic cortex (IL) projections that are thought to play a role in the expression of fear memories in rodents (circuit methods), and potentially in human depression (imaging methods). Was the ILàBLA considered for any of the outcomes? This is relevant in both the introduction and discussion to properly frame the interpretation of circuit manipulation outcomes and their alternatives. Lines 104-107

Author's Response: We appreciate this important point. The IL→BLA projection is well established and may indeed contribute to some of the behavioral effects associated with IL stimulation. While our study focuses on the necessity of the IL→RE→vHIPP pathway, we agree that interactions with other limbic structures such as the amygdala are likely relevant. We have therefore added text in the Introduction and Discussion acknowledging the potential involvement of the IL→BLA pathway and its possible role in the interpretation of circuit manipulation outcomes (lines 106–109 and 479–489).

4. "…the correlates of synaptic plasticity…" These studies do not establish a correlation between the evoked potentials and synaptic plasticity. Instead, the evoked potential outcomes could be attributed to synaptic plasticity. Still, there are physiological alternatives to long-term potentiation (LTP) in these paradigms, and there is no clear evidence of hippocampal involvement. Line 114

Author's Response: We agree with this point of the reviewer and we have revised the wording in the manuscript to avoid implying a direct correlation between evoked potentials and synaptic plasticity or hippocampal LTP (lines 112–116).

5. Several IL circuits may contribute to reduced anxiety and increased sociability. In this regard, it's great that the authors added the PL control in the DREADD experiments, but the amygdala is also an obvious target for these experiments; it'd be beneficial to know what's happening there throughout the experiments.

Author's Response: The reviewer is indeed right that amygdala might also contribute to the observed effects. As suggested above (point 3), we have added text in both the introduction and discussion to acknowledge the BLA involvement. (lines 106–109 and 479–489)

6. Is there a quantification of the distribution of AAV-hM3d+ cells for PL vs. IL surgical targets?

Author's Response: We thank the reviewer for highlighting this important point. This quantification is now provided as part of the comprehensive histological analysis presented in new Supplementary Figure 7.

7. What do the sample sizes mean in the LTP experiments? Cells, slices or mice?
Author's Response: We apologize for the lack of clarity. In LTP experiments, sample size corresponds to the number of measured cells. This is now clarified in the figure legend.

8. Do IL neurons synapse on inhibitory neurons, excitatory neurons, or both? Is there any more specificity to which types of cells IL targets to help explain the behavior-related calcium dynamics?

Author's Response: We appreciate the reviewer's attention to this detail. In the circuit examined here, IL activates excitatory glutamatergic neurons in the nucleus reuniens, which project to the ventral hippocampus. These projections are thought to influence hippocampal pyramidal neurons and local interneurons, although the precise cellular interactions remain incompletely understood (PMID: 33766671). We have clarified this point in the revised manuscript (lines 450–452).

9. There is text throughout Fig4 that is too small.
Author's Response: We thank the reviewer for noticing this apparent mistake and apologize for it. We increased the font size in the revised version of the figure.

10. It would be important to look at the BLA and central amygdala regions for changes in IEGs.
Author's Response: We thank the reviewer for pointing out this important point. We agree that elucidating the implication of the amygdala in these processes would greatly expand the understanding of the role of the IL→RE→vHIPP circuit and its interaction with other crucial circuits implicated in depression, and we aim to implement this component in our follow-up studies. As mentioned above (Points 3 and 5), we believe that this question would require a proper and in-depth investigation of amygdala involvement, as it could involve multiple, complementary, and potentially compensatory mechanisms. Although IEG analysis — in particular spatial quantification through immunohistochemistry — would provide interesting initial insights, the results could be difficult to interpret without subsequently silencing the three (or more) major entry points of the IL→RE→vHIPP circuit into the amygdala, each

of which may exert distinct and potentially opposing effects on amygdala engagement following IL stimulation.

11. How were images selected for the IEG and synaptic quantification in Fig4?
Author's Response: We agree that these experiments were not clearly presented. For the synaptic quantification (VGLUT1, VGLUT2, GAD65), coronal sections were selected at matched anteroposterior levels and imaging fields were positioned within defined CA1 layers (SLM for VGLUT2/GAD65 and SR for VGLUT1). This procedure has now been clarified in the revised Methods section (lines 824–842) (IEG expression was measured by qRT-PCR from microdissected tissue and therefore did not involve image selection).

12. Line 288-289, Do these experiments reveal anything about the IL>vHC or REàvHC component of the proposed ILàREàvHC circuit in antidepressant-like effects? It appears to demonstrate only the IL > RE component and IL modulation of vHC.
Author's Response: We agree that this point is critical to properly interpret the complete involvement of this circuit in the processes we are studying. We have conducted new series of experiments with selective chemogenetic inhibition of RE→HIPP projections, that blocked mPFC stimulation-induced restoration of hippocampal aLTP and mitigated the antidepressant effects of ketamine. These data have been presented in a new main Figure 7. (lines 330–347).

13. Lines 304-306. I don't follow the logic that ketamine provides insight into the vHC circuitry. While it seems plausible, and maybe even likely, that the NMDA receptors in the vHC play a role in the antidepressive effects of ketamine. While these experiments isolate IL>RE pathways (although RE projecting neurons located in IL likely target many structures), there is no isolation of the RE>vHC pathway.
Author's Response: We understand the reviewer's concern. This point has now been addressed by the new RE→HIPP projection-specific inhibition experiments (Fig. 7) that provide direct functional evidence: silencing RE neurons that project to the vHIPP blocks both LTP restoration (Fig. 7d,e) and ketamine's antidepressant-like effects (Fig. 7h,i). Combined with the IL→RE inhibition data (Figs. 5–6), these results establish the functional necessity of each segment independently. We have revised the manuscript at the indicated lines 330–347.

14. What is the evidence for vHC pathways in the circuit manipulations? The authors show several functional correlations (e.g., IEGs), but not the functional role for the RE>vHC pathway. Instead, I would say the IL-RE pathway modulates vHC. Line 321, also Line 430, 437

Author's Response: We completed this gap with the new experiments specifically inhibiting RE>vHIPP pathway shown in Fig. 7 (lines 330–347).

15. The description of the morphological analysis needs more information about the acquisition hardware (e.g., confocal microscope), how slices were selected, how ROIs were chosen, etc.

Author's Response: We acknowledge that this information was missing from the original manuscript. We added a detailed description of the morphological analysis methodology, including acquisition hardware, slice selection criteria, and ROI delineation procedures, in the Methods section (lines 817–842).

16. The histology is not described in the methods.
Author's Response: We apologize for this omission. A complete description of the histological procedures has now been added to the Methods section (Lines 798–821 and 874–904).

Reviewer #3 (Remarks to the Author):
In this manuscript, Veleanu et al. present novel evidence implicating the mPFC–RE–CA1 circuit in the antidepressant response to chronic stress in mice. Specifically, they demonstrate that overnight chemogenetic activation of hM3Dq-expressing excitatory neurons in the infralimbic cortex (IL) of the mPFC induces antidepressant-like behavioral effects, rescues stress-induced deficits in CA3–CA1 LTP in hippocampal slices, alters calcium dynamics in the ventral CA1 during the tail suspension test (TST), and modifies gene expression and Vglut2-positive puncta density in the ventral hippocampus. Importantly, they show that the nucleus reuniens (RE) is required for the antidepressant effect of IL stimulation and that selective chemogenetic inhibition of the mPFC–RE pathway blocks the antidepressant effect of ketamine.

We thank the reviewer for the careful evaluation of our manuscript and for the constructive and insightful comments. We particularly appreciate the suggestion to experimentally verify the net excitatory effect of IL stimulation, which prompted us to perform additional validation experiments and helped strengthen the robustness of our conclusions. We have carefully addressed the points raised below.

While this study offers a compelling circuit-level framework connecting prefrontal modulation to hippocampal plasticity in depression, several conceptual and technical issues need to be addressed to clarify the mechanistic basis of the findings:

1. Unclear nature of the IL perturbation: The authors claim that activation of IL excitatory neurons produces antidepressant effects, yet the effect of this perturbation on network

activity is uncharacterized. Previous theoretical and experimental studies demonstrate that cortical circuits represent inhibition-stabilized networks, whereas cortical excitation can result in paradoxical decreases in activity, depending on the extent and nature of the perturbation (eg PMID: 32598278). Without direct measurements of IL spiking in response to the perturbation, the interpretation of downstream effects remains speculative.

Author's Response: We thank the reviewer for valuable suggestion. We addressed this important point experimentally; Our new data show that the whole-cell recordings from hM3Dq-expressing IL pyramidal neurons show increased synaptically driven responses and intrinsic excitability following C21 application (Supplementary Fig. 1; lines 141–148).

2. Time course of chemogenetic effects: The manuscript lacks a detailed assessment of the acute versus overnight effects of C21. Acute intraperitoneal injections and chronic delivery via drinking water may differ significantly in their impact. It is unclear whether the behavioral and plasticity effects are due to the direct impact of IL activation or compensatory mechanisms arising from prolonged stimulation.

Author's Response: We thank the reviewer for this important consideration. We have directly compared acute and overnight C21 administration across multiple readouts. Acute C21 (0.75 mg/kg, i.p., 2h) did not produce antidepressant-like effects in the FST or TST (Supplementary Fig. 2k, l) and did not restore aLTP in hippocampal slices (Supplementary Fig. 3i, j, m). In contrast, overnight C21 administration via drinking water was required for both behavioral and plasticity effects. These data are now explicitly highlighted in the revised version of the manuscript (lines 164–166 and 210–212).

3. Fiber photometry results are difficult to interpret: State-dependent modulation of hippocampal Ca^{2+} signals (increased during struggle, decreased during immobility) could reflect changes in motor behavior rather than circuit engagement per se. Without monitoring other behavioral states (e.g., quiet wakefulness, active exploration in the home cage), and ideally without single-unit resolution, it's challenging to attribute the observed changes to antidepressant effects rather than confounds like arousal or movement.

Author's Response: We appreciate this concern and performed additional analyses to dissociate Ca^{2+} signals from motor activity (Supplementary Fig. 4). Ca^{2+} dynamics during spontaneous quiet wakefulness to active exploration transitions and motor activity to struggle onset during the TST were comparable between post-CDM and post-C21 conditions, and Ca^{2+} peak amplitude did not correlate with movement intensity. These analyses indicate that the observed Ca^{2+} changes reflect circuit modulation rather than motor or arousal confounds (lines 232-238).

4. Lack of direct measurements from IL→CA1 or RE→CA1 pathways: The authors propose that IL stimulation modulates hippocampal LTP via the RE. However, the synaptic and cellular mechanisms remain poorly defined. Electrophysiological recordings from IL–CA1 and RE–CA1 projections would greatly strengthen the mechanistic claims. Recordings from these pathways have been preveriously performed in vivo (e.g. PMID: 9204945, 37919286) and in brain slices (PMID: 32065917). Furthermore, it s unclear whether the chemogenetic manipulation that rescued LTP was performed in vivo (i.e., prior to slice preparation) or ex vivo (i.e., in the slice).

Author's Response: We thank the reviewer for this valuable suggestion and have extended the manuscript to further address circuit-level mechanisms linking IL activity to hippocampal plasticity. In addition to projection-specific inhibition of RE→vHIPP neurons, which abolished both IL-induced restoration of aLTP and ketamine's behavioral effects (Fig. 7d–i; lines 330–347), we now include LFP recordings from IL, RE, and hippocampus. These data (Supplementary Fig. 9; lines 348–355 and 473–478) show that CDM disrupts inter-regional coherence and theta–gamma phase–amplitude coupling across the circuit, both of which are partially restored by IL activation.

While direct projection-specific electrophysiological recordings from IL→CA1 and RE→CA1 pathways would provide additional mechanistic resolution, these experiments fall beyond the scope of the present study. The combined circuit-level recordings and pathway-specific manipulations nevertheless support a role for RE-mediated coordination of hippocampal activity.

We also clarified that all chemogenetic manipulations were performed in vivo prior to slice preparation (C21 administered overnight via drinking water; line 207).

5. Effect of mPFC–RE inhibition on functional connectivity: The strong behavioral effects of mPFC–RE inhibition are compelling. However, the physiological consequence of this manipulation is not addressed. fEPSP recordings from the mPFC–RE pathway could reveal whether synaptic strength or short-term plasticity is altered by this intervention, and how it relates to behavior.

Author's Response: We thank the reviewer for this thoughtful suggestion and agree that assessing the physiological consequences of mPFC–RE inhibition would further refine the mechanistic interpretation. While direct electrophysiological recordings from the mPFC→RE synapse were not included within the scope of the present study, the functional properties of this pathway have been characterized previously (PMID: 30107061). Newly generated LFP recordings (Supplementary Fig. 9) show oscillatory coupling deficits in the mPFC→RE pathway, counteracted by IL stimulation, suggesting a role of the functional connectivity of this connection in the studied mechanisms. We

have incorporated a brief discussion of these findings to better contextualize our results (lines 434–436).

6. Circuit dysfunction in depression is not well characterized: The authors argue that the IL–RE–vHIP circuit is involved in antidepressant responses, yet provide limited data on how this circuit is dysregulated in the CDM depression model. Although hippocampal LTP is shown to be abolished by chronic stress, baseline activity or synchrony in the IL, RE, or CA1 is not assessed. This gap limits the interpretability of how the circuit is pathologically altered and how IL stimulation restores function.
Author's Response: We thank the reviewer for the suggestion. To address this, we performed LFP recordings to assess circuit-level activity and synchrony in the IL, RE and HIPP. The new data presented in Supplementary Fig. 9 (lines 348–355 and 473–478) demonstrate that CDM disrupted inter-regional coherence and theta–gamma phase-amplitude coupling across the circuit. IL activation reversed part of these deficits.

7. Broader role of RE in cognitive function and disease: Chemogenetic and electrical manipulations of the RE have been shown to affect working memory and contribute to hyperexcitability in Alzheimer's models. It would be valuable for the authors to briefly discuss these findings in light of their results, particularly regarding potential off-target or pleiotropic effects of RE inhibition.
Author's Response: We appreciate this important point. The broader role of the RE in hippocampal–cortical network function and disease is now addressed in the revised Discussion to better contextualize our findings (Lines 433–445).

REVIEWER COMMENTS

Reviewer #1 (Remarks to the Author):

The authors have addressed the concerns satisfactorily. I have a few remaining minor observations for your consideration

Author's Response: We thank the reviewer for their positive assessment and for acknowledging that the major concerns have been satisfactorily addressed. We appreciate the additional minor observations and have carefully responded to each of them to further improve the clarity and quality of the manuscript.

1) Line 602 "Behavior was recorded for 10 min, and total distance traveled and time spent in the center area were analyzed."
How was it analyzed?

Author's Response: We thank the reviewer for pointing out this omission. As for the other behavioral tests (NOE, 3ChT), video recordings of the OFT were analyzed post hoc using Ethovision XT (Noldus, Netherlands). The Methods section has been updated consequently. (Line 603)

2) Line 639-645 Which session included the quantification of sucrose licking behavior?
Author's Response: We apologize for the lack of clarity. Sucrose licking was quantified continuously across all drinking sessions of the nose-poke paradigm. We added this information in the methods. (Lines 645-646)

3) Line 140 (Fig. 1a-b; Supplementary Fig. 7a-c).
Author's Response: We agree with the reviewer. We have renumbered the Supplementary Figures so that their numerical order matches their order of appearance. Supplementary Fig. 7 (histological validation) has been moved to Supplementary Fig. 1, and subsequent Supplementary Figures renumbered accordingly.

4) Line 656-7 "Reverse transcription was performed with 1 mg of total RNA using M-MLV reverse transcriptase (Promega)."
Author's Response: We thank the reviewer for noticing this apparent mistake and apologize for it. The reverse transcription was performed with 1 μg (microgram) of total RNA. This has been corrected in the text (line 658).

5) In Figure 3D, shouldn't the results at -4 s and -2 s be the same as those shown in Figure 3C at 2 s and 4 s (specifically the higher struggle/delta F/F levels post-C21)?
Author's Response: Figures 3c and 3d are aligned to two distinct behavioral events: 3c to struggle onsets (time 0 = transition from immobility to struggle), and 3d to immobility onset (time 0 = transition from struggle to immobility). Because struggle bouts can last much longer than 4 s, the +2/+4 s window in 3C (early struggle, captured from onset) and the −4/−2 s window in 3D (late struggle, captured before offset) do not fall within the same bouts and are not expected to show identical values. The two panels therefore convey complementary state-dependent effects: how IL activation scales the response at struggle initiation versus how it further suppresses activity as the animal disengages, rather than mirrored views of the same event.

6) Lines 239-243 "These findings demonstrate that IL activation bidirectionally modulates HIPP

activity during behavioral transition in the TST, but not homecage exploration, increasing excitatory neuron responses during struggle and further suppressing these responses during immobility, with concurrent changes in inhibitory neuron dynamics. This increased state-dependent dynamics suggests that IL activation improves hippocampal "gain" in stress-related behaviour, potentially contributing to its antidepressant effects."
This is an interesting finding; how might this translate to a human social context?

Author's Response:
We thank the reviewer for raising this important point. While the question of translation to human social contexts is highly relevant, we believe that any direct inference from the current data would be premature. Our experiments were specifically designed to probe state-dependent hippocampal dynamics during stress-related behavioral transitions in mice, and do not directly address the processing of complex social stimuli.
That said, our findings point toward a modulation of hippocampal gain that is dependent on behavioral state, which may more generally influence how salient information is encoded and processed. Determining whether such gain modulation extends to socially relevant stimuli (and how this might relate to altered social processing in depression) will require dedicated experimental paradigms. We are currently pursuing this direction in follow-up work, with a focus on more precisely characterizing the valence and salience of stimuli affected by hippocampal gain modulation.

7) Line 250 Could you please define 'IEGs'?
Author's Response: We apologize for this omission; we modified the text consequently.

8) Figure 5e, What is the interpretation for the null effect of mPFC activation on OFT performance? One would expect the results to be consistent with those shown in Figure 1h
Author's Response:This experiment was designed to isolate the contribution of the RE to the IL stimulation-induced effect. The interpretable comparison here is between the hM3Dq mPFC + mCherry RE and the hM3Dq mPFC + hM4Di RE groups, the latter showing a lower time spent in the center, suggesting abolition of the mPFC stimulation effect. The hM4Di RE alone group is not a control for IL activation, as it remains unclear whether manipulation of this nucleus alone would affect the OFT readout. Furthermore, direct numerical comparison between Fig. 5e and Fig. 1h is not warranted, as absolute OFT center-time values are known to be highly sensitive to cohort and setup-specific conditions, and are therefore typically interpreted within rather than across experiments.

9) Figure 6f, In this experiment, the RE inhibits the IL, which in turn prevents the IL from activating the RE (rather than the mPFC-mediated inhibition of the RE). Wouldn't it be more precise for Figure 6f to explicitly show this—perhaps by indicating the experimental inhibition of the mPFC and the resulting reduction in RE activity?
Author's Response: We agree that the scheme in Figure 6f was misleading and apologize for the confusion. In this experiment, the retroAAV-Cre construct, injected in the RE, is retrogradely transported from axonal terminals to the somas of RE-projecting IL neurons, allowing Cre-dependent expression of hM4D(Gi) specifically in mPFC→RE projection neurons. We have updated the scheme by adding a cross directly on the mPFC→RE projection arrows, to clarify that the manipulation selectively blocks this pathway, rather than suggesting an mPFC-driven inhibition of the RE itself.

10) Figure 7f A similar observation was made: despite mPFC activation, when the RE is inhibited,

there is no Hippocampal activation (LTP). Instead of an inhibition of the Hippocampus. This follows the same concept seen in Figure 7i: RE inhibition does not directly inhibit the HIPP; rather, RE activity is required to activate the HIPP.
Author's Response: The reviewer is again right. We have applied the same correction to Fig. 7f and Fig. 7i: a cross has been added on the RE→vHIPP projection arrows to indicate the selective blockade of this pathway.

11) Why was LTP analyzed in one experiment, while the FST was used as the output in the second?"
Author's Response: The convergence of C21 and ketamine on both aLTP and FST was already established in Figs. 5 and 6, where each intervention was tested on both readouts. For the final RE→vHIPP segment (Fig. 7), we adopted a complementary design : C21 paired with aLTP and ketamine with FST, to test circuit necessity at both levels without duplicating all readouts and thus according to 3Rs (reduction rule) in animal ethics using less animals.

12) Line 402 "The mPFC does not send direct excitatory projections to the HIPP; its primary excitatory route is relayed through the RE, which innervates preferentially intermediate and ventral CA1 and the subiculum with comparatively sparse projections to the dHIPP" This sentence is a little confusing I suggest as follows "The mPFC does not send direct excitatory projections to the HIPP; its primary excitatory route is relayed through the RE, which preferentially innervates the intermediate and ventral CA1 and the subiculum, with comparatively sparse projections to the dHIPP"
Author's Response: We thank the reviewer for this suggestion, and modified the text consequently.

13) Figure 8: While the model is functional, would it be more elegant to show sagittal sections instead of coronal ones?
Author's Response: We thank the reviewer for this suggestion. We replaced the coronal sections by a sagittal representation.

14) Line 417 "We provide compelling evidence that chronic stress-induced impairments in hippocampal LTP can be fully reversed by stimulation of the IL"
Please refer to the figures
Author's Response: As suggested by the reviewer, we have added the according reference to the figure.

15) Line 424 "between active and passive coping behavioral states"
Please refer to the figures
Author's Response: As requested, we have added the corresponding reference.

16) Line 436 "modulate RE firing patterns"
Please refer to the figures
Author's Response: We apologize for this omission. Since this sentence referred to the literature, we have added the missing reference.
17) Line 511 "DTI-based tractography in SCC DBS patients has identified three critical white matter bundles"
18) Diffusion tensor imaging (DTI)
Author's Response: We apologize for this acronym omission; we modified the text consequently.

Reviewer #2 (Remarks to the Author):
The authors have addressed all my concerns.

Author’s Response: We sincerely thank the reviewer for the careful evaluation of our revised manuscript and for confirming that all concerns have been addressed.

Reviewer #3 (Remarks to the Author):
The authors have comprehensively addressed my main previous concerns. The additional experiments and data analyses significantly strengthen the manuscript's primary claims. From my perspective, the manuscript is now ready for publication.

Author’s Response: We greatly appreciate the reviewer encouraging assessment, particularly the recognition that the additional experiments and analyses have strengthened the manuscript and that it is now suitable for publication.

We thank all 3 reviewers for their constructive comments throughout the review process, which have helped us substantially improve the quality and clarity of our work.
