# Supplementary material for: The mPFC-reuniens-hippocampus pathway links brain circuitry and neural plasticity in antidepressant response
Source: Nat Commun. 2026 Sep 1;17:9361. doi: 10.1038/s41467-026-77248-y (PMC13534506; doi:10.1038/s41467-026-77248-y)
Supplement: Supplementary file 3 [file 41467_2026_77248_MOESM3_ESM.pdf]

nature portfolio

Corresponding author(s): Stefan Vestring
Last updated by author(s): 02.07.2026

Reporting Summary

Nature Portfolio wishes to improve the reproducibility of the work that we publish. This form provides structure for consistency and transparency in reporting. For further information on Nature Portfolio policies, see our Editorial Policies and the Editorial Policy Checklist.

Statistics

For all statistical analyses, confirm that the following items are present in the figure legend, table legend, main text, or Methods section.

n/a	Confirmed	
☐	☒	The exact sample size (n) for each experimental group/condition, given as a discrete number and unit of measurement
☐	☒	A statement on whether measurements were taken from distinct samples or whether the same sample was measured repeatedly
☐	☒	The statistical test(s) used AND whether they are one- or two-sided<br>*Only common tests should be described solely by name; describe more complex techniques in the Methods section.*
☐	☒	A description of all covariates tested
☐	☒	A description of any assumptions or corrections, such as tests of normality and adjustment for multiple comparisons
☐	☒	A full description of the statistical parameters including central tendency (e.g. means) or other basic estimates (e.g. regression coefficient) AND variation (e.g. standard deviation) or associated estimates of uncertainty (e.g. confidence intervals)
☐	☒	For null hypothesis testing, the test statistic (e.g. F, t, r) with confidence intervals, effect sizes, degrees of freedom and P value noted<br>*Give P values as exact values whenever suitable.*
☒	☐	For Bayesian analysis, information on the choice of priors and Markov chain Monte Carlo settings
☒	☐	For hierarchical and complex designs, identification of the appropriate level for tests and full reporting of outcomes
☒	☐	Estimates of effect sizes (e.g. Cohen's d, Pearson's r), indicating how they were calculated

Our web collection on statistics for biologists contains articles on many of the points above.

Software and code

Policy information about availability of computer code

Data collection	The behavioural experiments were recorded with IC Capture 4.0 (The Imaging Source). IntelliCage data were acquired with IntelliCage Plus v3.6.2.0 (TSE Systems). qRT-PCR data were obtained with 7300 System SDS v1.3.1 (Applied Biosystems). Electrophysiological data were obtained using Patchmaster NEXT 1.2 (HEKA). Microscopy images were acquired using ZEN Blue 3.4 (Cell Discoverer 7), both Carl Zeiss. Fiber photometry data were collected using LabVIEW 2024 (National Instruments). LFP data were collected using the Open Ephys GUI v1.0.1.
Data analysis	OFT, NOE and 3ChT data were analyzed with EthoVision XT 14 (Noldus); behaviour during fiber photometry was tracked using DeepLabCut 2.2. IntelliCage data were analyzed with IntelliCage Plus v3.6.2.0 (TSE Systems). Immunofluorescence images were analysed with Fiji (ImageJ 1.54, 2024), and Ilastik 1.4.0. Fiber photometry and LFP data were analyzed with custom scripts written in MATLAB R2022b (MathWorks) and Python 3.10. All statistical analyses were performed in GraphPad Prism 10.0. Custom MATLAB and Python code is available at https://github.com/mveleanu/veleanu_et_al (archived on Zenodo, DOI: 10.5281/zenodo.21111701).

For manuscripts utilizing custom algorithms or software that are central to the research but not yet described in published literature, software must be made available to editors and reviewers. We strongly encourage code deposition in a community repository (e.g. GitHub). See the Nature Portfolio guidelines for submitting code & software for further information.

Data

Policy information about availability of data

All manuscripts must include a data availability statement. This statement should provide the following information, where applicable:

- Accession codes, unique identifiers, or web links for publicly available datasets
- A description of any restrictions on data availability
- For clinical datasets or third party data, please ensure that the statement adheres to our policy

The data supporting the findings of this study are available within the paper, its Supplementary Information and the associated Source Data file. Source data are provided with this paper. The custom code used for data analysis is publicly available on GitHub (https://github.com/mveleanu/veleanu_et_al) and archived on Zenodo (https://doi.org/10.5281/zenodo.21111701). No datasets are under restricted access; materials are available from the corresponding author on request.

Research involving human participants, their data, or biological material

Policy information about studies with human participants or human data. See also policy information about sex, gender (identity/presentation), and sexual orientation and race, ethnicity and racism.

Reporting on sex and gender	NA
Reporting on race, ethnicity, or other socially relevant groupings	NA
Population characteristics	NA
Recruitment	NA
Ethics oversight	NA

Note that full information on the approval of the study protocol must also be provided in the manuscript.

Field-specific reporting

Please select the one below that is the best fit for your research. If you are not sure, read the appropriate sections before making your selection.

[x] Life sciences [ ] Behavioural & social sciences [ ] Ecological, evolutionary & environmental sciences

For a reference copy of the document with all sections, see nature.com/documents/nr-reporting-summary-flat.pdf

Life sciences study design

All studies must disclose on these points even when the disclosure is negative.

Sample size	Group size estimates were based on previous experience with the experimental design and the mouse models. For behavioral experiments, a formal power calculation was done with the Institute of Medical Biometry and Medical Informatics, University of Freiburg (n=10 per group for most experimental series). For RT-PCR, IHC we aimed for a group size of 6-8, for electrophysiological experiments 7-10, for fiberphotometry 5-7.
Data exclusions	Animals were excluded in case of health problems. For fiberphotometry, animals were excluded on the basis of implant problems, misplaced fiber, or in case of absence of calcium indicator signal. For LFP, electrodes were excluded when not in the expected area. Statistical outliers were excluded using the ROUT method with Q = 1%, as described in the Methods.
Replication	Key behavioural, molecular and electrophysiological findings were confirmed across independent cohorts of animals wherever applicable; where an experiment was performed in a single cohort, this is indicated in the Source Data file. Data from independent replicates were pooled and are presented in the figures. No findings failed to replicate.
Randomization	For all behavioral, molecular, fiberphotometry and electrophysiological studies, mice were randomly assigned to groups, and animals were of the same strain, age range and housing conditions.
Blinding	In all applicable experiments, blinding was implemented to minimize bias during both data collection and analysis. In behavioural studies, experiments were either videotaped or automatically tracked, and behavioural scoring was conducted offline by researchers blinded to the treatment conditions, using coded videos without identifying information. For qRT-PCR and immunohistochemistry/synaptic-density experiments, samples were labelled with anonymized codes prior to processing, and all quantification and analysis were performed by researchers blinded to the group identities (immunolabelling quantification was additionally automated using Ilastik). In electrophysiological experiments, experimenters were blinded to the treatment condition/experimental condition.

Reporting for specific materials, systems and methods

We require information from authors about some types of materials, experimental systems and methods used in many studies. Here, indicate whether each material, system or method listed is relevant to your study. If you are not sure if a list item applies to your research, read the appropriate section before selecting a response.

Materials & experimental systems

n/a	Involved in the study
☐	☒ Antibodies
☒	☐ Eukaryotic cell lines
☒	☐ Palaeontology and archaeology
☐	☒ Animals and other organisms
☒	☐ Clinical data
☒	☐ Dual use research of concern
☒	☐ Plants

Methods

n/a	Involved in the study
☒	☐ ChIP-seq
☒	☐ Flow cytometry
☒	☐ MRI-based neuroimaging

Antibodies

Antibodies used

Anti-VGLUT1 (Guinea Pig polyclonal) - Millipore
Cat. Number: AB5905
lot: 4141952
RRID: AB_2301751
dilution:1:1000

Anti-VGLUT2 (Guinea Pig polyclonal) - Millipore
Cat. Number: AB2251-I
lot: 4137617
RRID: AB_1587626
dilution:1:1000

Anti-GAD65 [GAD6] (Mouse monoclonal) - Abcam
Cat Number: ab26113
lot: GR3308495-2
RRID: AB_448989
dilution:1:500

Secondary antibody goat polyclonal anti-guinea pig Alexa Fluor 594, Invitrogen
Cat Number: A-11076
lot: 2540867
RRID: AB_2534120
dilution:1:1000
Secondary antibody goat polyclonal anti-mouse Alexa Fluor 488, Invitrogen
Cat Number: A-32723
lot: XA336883
RRID: AB_2633275
dilution:1:1000

Validation

Anti VGLUT1 (guinea pig polyclonal): Validated by Millipore and used in multiple studies as per company's website (ie. Turner et al., 2015, Nature; Brigidi et al., 2015, Nat Commun).
Anti-VGLUT2 (guinea pig polyclonal): Validated by Milipore and used in multiple studies as per company's website (ie. Modol et al., 2019, Neuron; Veleanu et al., 2022, PNAS; Jullié et al., 2019, Neuron).
Anti-GAD65 (mouse): Validated by abcam and used in multiple studies as per company's website (ie. Chai G et al., 2021, Neuron; Kang W et al., 2022, Nat. communications).
Secondary antibody anti-guinea pig Alexa Fluor 594: Validated by Invitrogen and used in multiple studies as per company's website in Stillman et al., 2023, Nat. Communications; Chen et al., 2023, IScience).
Secondary antibody goat polyclonal anti-mouse Alexa Fluor 488: Validated by Invitrogen and used in multiple studies as per company's website in Monteil et al., 2023, Nat. Communications; Zheng et al., 2023, Nature Structural Molecular Biology).

Animals and other research organisms

Policy information about studies involving animals; ARRIVE guidelines recommended for reporting animal research, and Sex and Gender in Research

Laboratory animals

Male and female wild type mice C57BL/6J (Mus musculus; RRID: IMSR_JAX:000664) were obtained from Charles River or Janvier (Le

	Genest-Saint-Isle, France) . Mice were at least 8 weeks old at the start of the experiment and housed in a (21 ± 2 °C) and humidity (55 ± 10 %) controlled environment with ad libitum access to food and water, maintained on a 12h light-dark cycle.
Wild animals	The study did not involve wild animals.
Reporting on sex	Male and female mice were included with equal distribution across experiments. Exploratory analyses of potential sex effects were performed, and no relevant differences were observed; however, the experiments were not adequately powered to reliably detect sex differences.
Field-collected samples	The study did not involve samples collected in the field.
Ethics oversight	All procedures were approved by animal review boards in Germany (Regierungspräsidium Freiburg, licenses G-19/44, G-20/141, G-22/116, G-24/033 and G-25/032) or France (CREMEAS, APAFIS n°2020042818477700). The Chronobiotron facility is registered for animal experimentation (Agreement A67-2018-38). Animal care and use protocols followed national and international standards.

Note that full information on the approval of the study protocol must also be provided in the manuscript.

Plants

Seed stocks	*Report on the source of all seed stocks or other plant material used. If applicable, state the seed stock centre and catalogue number. If plant specimens were collected from the field, describe the collection location, date and sampling procedures.*
Novel plant genotypes	*Describe the methods by which all novel plant genotypes were produced. This includes those generated by transgenic approaches, gene editing, chemical/radiation-based mutagenesis and hybridization. For transgenic lines, describe the transformation method, the number of independent lines analyzed and the generation upon which experiments were performed. For gene-edited lines, describe the editor used, the endogenous sequence targeted for editing, the targeting guide RNA sequence (if applicable) and how the editor was applied.*
Authentication	*Describe any authentication procedures for each seed stock used or novel genotype generated. Describe any experiments used to assess the effect of a mutation and, where applicable, how potential secondary effects (e.g. second site T-DNA insertions, mosiacism, off-target gene editing) were examined.*
